# Comparative Investigation of pH–Dependent Availability of Pancreatic Enzyme Preparations In Vitro

**DOI:** 10.3390/ph17050552

**Published:** 2024-04-25

**Authors:** Amy Todd, Emma Bennett-Huntley, Jonas Rosendahl, Jürgen Schnekenburger, Waldemar Uhl

**Affiliations:** 1 Viatris/Mylan Pharma UK Ltd., Sandwich CT13 9ND, UK; 2 Department of Internal Medicine I, University Hospital Halle (Saale), 06120 Halle, Germany; 3 Biomedizinisches Technologiezentrum (BMTZ), Faculty of Medicine, University of Münster, 48149 Münster, Germany; 4 Department of General and Visceral Surgery, St.-Josef-Hospital, Clinic of Ruhr-University Bochum, 44791 Bochum, Germany

**Keywords:** digestion, duodenum, enzyme replacement therapy, pancreatic digestive enzymes, pancreatic insufficiency

## Abstract

This study aimed to compare different pancreatic enzyme preparations (PEPs) available in Germany regarding particle geometry and size, and to evaluate enzyme activity under physiologically relevant conditions in vitro. Pancreatic endocrine insufficiency is characterized by deficiency of pancreatic enzymes resulting in maldigestion. It is orally treated by pancreatic enzyme replacement therapy. The formulations differ in their physical properties and enzyme release behavior, potentially resulting in inconsistent dosages and poor interchangeability of products. A total of 25 products were analyzed for particle size and number of particles per capsule. Enzyme activities of lipase, amylase, and protease were measured by digestion of olive oil emulsion, starch, and casein, respectively. To analyze enzyme release, gastric environments were simulated by incubating PEPs at pH 1, 4, or 5. Duodenal conditions were simulated by subsequent incubation at pH 6. Regarding physical properties and enzyme release kinetics, considerable differences between different PEPs were found. Furthermore, compared to the label claim, excess lipase activity was observed for most products, reaching up to 148%. These in vitro results suggest poor interchangeability of PEPs, potentially explained by physical and release characteristics. Physicians and patients should be aware of the potential gap between label claims and the real-life performance of different PEPs.

## 1. Introduction

PEI (pancreatic exocrine insufficiency) is caused by inactivation, inadequate production, and/or insufficient secretion of pancreatic enzymes, such as amylase, lipase, and proteases, and can lead to malnutrition [1,2]. Underlying causes are most frequently chronic pancreatitis, often accompanied by pancreatic fibrosis and cystic fibrosis [3,4]. Furthermore, PEI is prevalent in patients with type I or type II diabetes [5]. Moreover, pancreatic cancer often leads to impairment in pancreatic exocrine secretion. Surgical procedures removing the affected tissue can lead to further digestive alterations, which may in turn contribute to PEI [6]. Furthermore, bariatric surgery can cause acute pancreatitis [7].

Pancreatic enzymes facilitate the digestion of macronutrients. Thus, patients with untreated PEI typically have difficulties in digestion, especially that of fat, and suffer symptoms of maldigestion and malnutrition, such as abdominal cramps, bloating, steatorrhea, and weight loss [8]. Furthermore, the depletion of nutrients leads to a reduced quality of life, as well as increased morbidity and mortality rates [1,9].

In order to treat PEI, exogenous PEPs (pancreatic enzyme preparations) are orally administered [10]. PERT (pancreatic enzyme replacement therapy) facilitates the improvement of the nutritional status by counteracting the cause of malnutrition and thereby improves the quality of life in PEI patients [1]. The HaPanEU (Harmonizing diagnosis and treatment of chronic pancreatitis across Europe) working group has defined several properties for an ideal PEP in order to mimic the natural process of digestion [9]. One is the resistance towards gastric acid and a rapid release of enzymes in the duodenum [9,11]. Furthermore, PEPs should mix well with meals, undergo gastric emptying together with meals, and mix with the duodenal chyme [9]. To meet these criteria, it has been suggested that a PEP particle should be below 2 mm or even 1.7 mm in diameter [12,13]. In the past, PEPs were often overfilled to ensure that the labeled activity is present at the end of shelf life. However, for safety and standardization reasons, EMA (European Medicines Agency) guidelines state that any PEP should be formulated to 100% of the claimed lipase activity [13]. A PEP fulfilling the above-mentioned criteria can be regarded as effective pancreatic enzyme replacement therapy.

Although the bioequivalence has not been shown, PEP products are often substituted by another product without monitoring [8]. For comparing different products and strengths, it is necessary to analyze relevant parameters of these PEPs, since the respective products could be marketed without required efficacy testing [8]. Due to differences in the actual enzyme activities in the duodenum, the substitution of products may lead to under- or overdosing [8]. Previous studies concluded that there were substantial differences in dissolution profile, particle size, and actual enzyme content between enzyme preparations of different products and, in some cases, different batches of the same product [12,14,15,16]. These properties define the available enzyme activity. However, no such study was conducted specifically on the enzyme preparations available in Germany after the HaPanEU guideline has been published in 2017. To investigate potential differences of PEP formulations, a more precise analysis with products available on the German market was conducted. A new parameter assessed in this study was the determination of particle number per capsule, which might define the ability of the PEP to mix homogenously with the meal and the chyme.

In summary, the objective of the current study was to analyze and compare the physical properties of particle size, particle number, and pH-dependent enzyme activities and the compliance with the label claim for various enzyme preparations available on the German market to determine the interchangeability/differences of the respective products. We found that the physical properties and enzyme release kinetics varied considerably between different PEPs. Additionally, excess lipase activity compared to the label claim was observed for most products. Altogether, our in vitro results suggest poor interchangeability of PEPs available on the German market.

## 2. Results

### 2.1. Physical Characterization

#### 2.1.1. Particle Imaging

**Figure 1:**
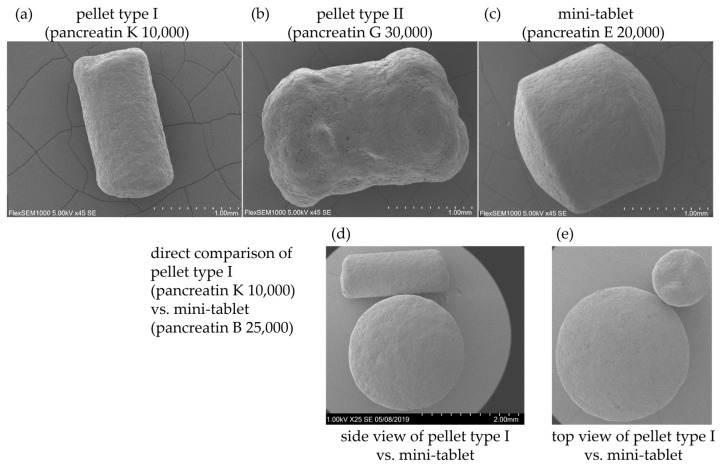
SEM micrographs of examples of the three identified presentations (**a**) pellet type I, (**b**) pellet type II, and (**c**) mini-tablets. Scale bar: 1.00 mm. Direct comparison of examples for pellet type I and mini-tablet in (**d**) side view and (**e**) top view of pellet type I.

Three distinct sample presentations were identified during the scanning electron microscope (SEM) analysis of all samples, which have been characterized as pellets (type I and II) and mini-tablets (MT) as presented in Figure 1a–c and Table 1.

Type I pellets (Figure 1a) were cylindrical in shape, approximately 0.9 mm in diameter and varying in length between 1 mm and 2.5 mm. Type I pellets were unique to pancreatin K products. Type II pellets (Figure 1b) were shaped similarly to type I pellets but generally larger in all orientations. Type II pellets were cylindrical, approximately 1.2 mm to 1.5 mm in diameter and varying in length between 1.2 mm and 3.0 mm. Examples of type II pellets have been observed for both mid and high strength products, although more commonly in higher strength products. MTs (Figure 1c) were the largest of the three types, shaped like a cylinder with a domed top and bottom. They were approximately 2.2 mm in their longest orientation (diagonally, top corner to bottom opposite corner, across the cylindrical center) and marginally smaller end to end and in diameter. Examples of MTs have been observed for both low and mid strength products, with a more frequent occurrence in mid strength products. Pancreatin A 20,000 and pancreatin J 10,000 were single tablet formulations with particle sizes of 11.39 mm and 10.31 mm, respectively.

#### 2.1.2. Particle Size

Obtained FERET Max D[v, 0.5] values measured for all products as well as their respective presentations are summarized in Table 2. When considering FERET Max D[v, 0.5] among type I pellets, pancreatin K 5000 had the smallest particle size at 1.45 mm and pancreatin K 25,000 had the largest size, up to 1.61 mm.

Of the type II pellets investigated, pancreatin E 40,000 had the smallest FERET Max D[v, 0.5] at 2.52 mm and pancreatin B 40,000 the largest at 2.58 mm.

Among the MTs, pancreatin C 20,000, pancreatin D 10,000, and pancreatin D 25,000 had the smallest FERET Max D[v, 0.5] at 2.61 mm. The largest FERET Max D[v, 0.5] was found in pancreatin A 10,000 at 2.63 mm.

#### 2.1.3. Particle Counting

Analysis of images taken for particle counting showed that capsules containing type I pellets had the largest number of individual particles and capsules containing MTs had the lowest. Particle count range for each of the preparations and strengths can be found in Table 3.

### 2.2. Enzymatic Analysis

#### 2.2.1. Enzyme Activities

The enzyme activities of lipase, amylase, and protease are available in Table 4. The actual lipase activity at pH 9 ranged from 99.1% (pancreatin K 25,000) to 148.1% (pancreatin B 20,000) of the label claim. Different strengths of the same product vary in the actual lipase activity compared to the label claim.

**Figure 2:**
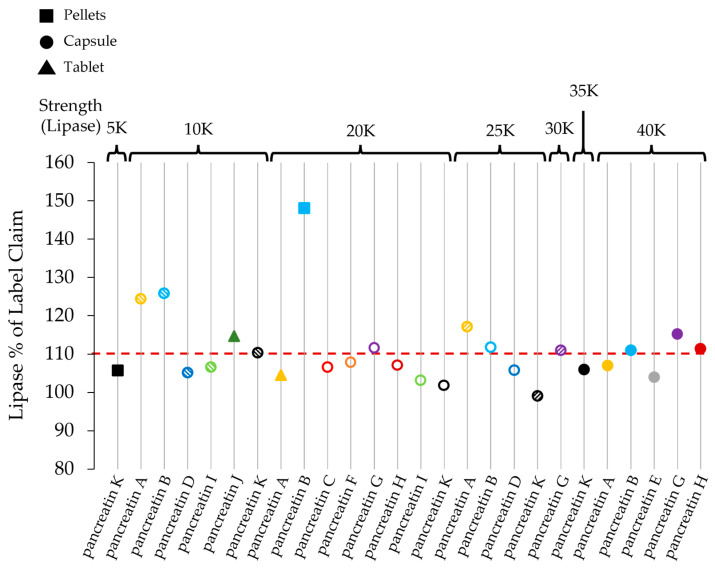
Lipase activity relative to the label claim (in %). Red dashed line represents a lipase activity of 110% of the label claim. The symbol (square, circle, triangle) refers to the type of presentation (pellets, capsule, tablet). Pellets: PEPs with free pellets which are dosed with a measuring scoop; Capsule: PEPs where individual pellets are contained in a capsule; Tablet: PEP consists of a single tablet. Each product (potentially with various strengths) has an individual color. The filling of the symbols was chosen so that the individual PEPs can be differentiated in the following Figure 3.

Large excess was found for pancreatin B (111.0% (40,000) to 148.1% (20,000)) followed by pancreatin A (104.5% (20,000) to 124.4% (10,000)) (Figure 2). The amylase activity measured was higher than the label claim for all of the products, ranging from 115.9% (pancreatin E 40,000) to 160.4% (pancreatin A 20,000). Similarly, the protease activity was higher than the label claim for all of the products, ranging from 107.3% (pancreatin D 25,000) to 165.0% (pancreatin K 5000).

#### 2.2.2. Enzyme Release

**Figure 3:**
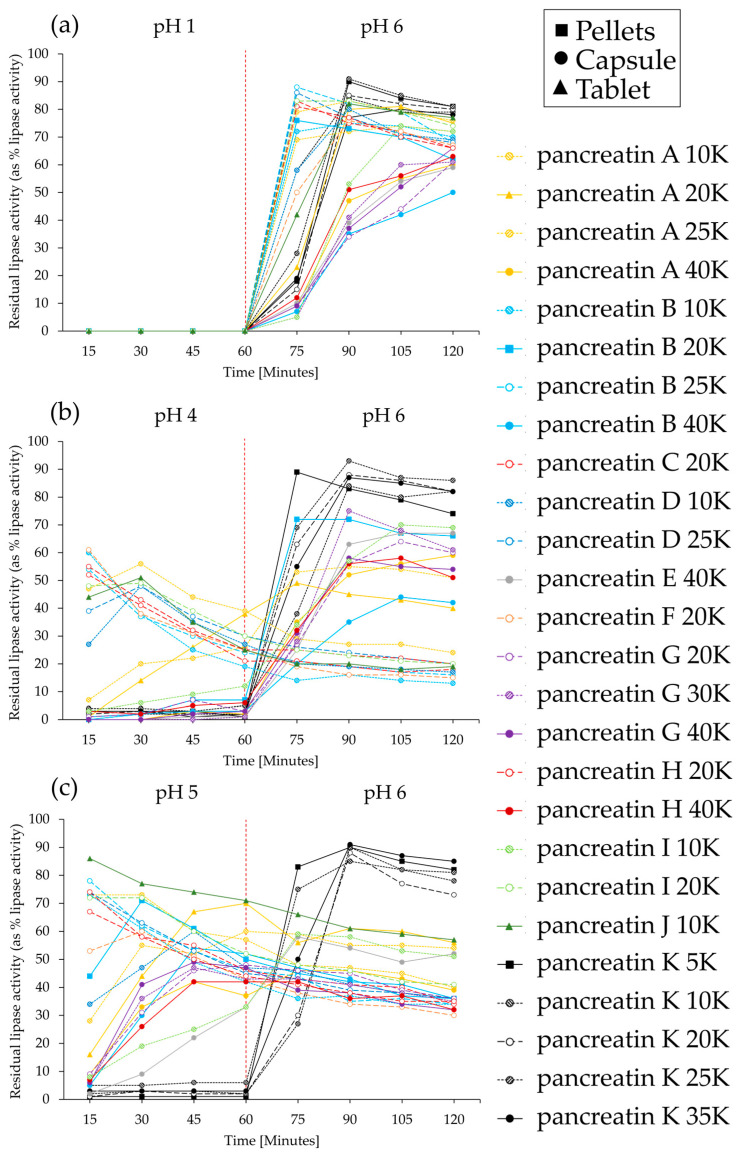
Lipase activity (as % lipase activity) vs. time (minutes). pH test for three value areas (**a**) 1 to 6, (**b**) 4 to 6, and (**c**) 5 to 6. After 60 min, the pH was increased from 1 (**a**), 4 (**b**), or 5 (**c**) to 6. Pellets: PEPs with free pellets which are dosed with a measuring scoop; Capsule: PEPs where individual pellets are contained in a capsule; Tablet: PEP consists of a single tablet. The red line indicates the change of pH at 60 min.

In experiments simulating the transition from stomach (pH 1) to small intestine (pH 6), very low (less than 6%) or no release of lipase activity was noticed for pancreatin K preparations during the first 60 min at simulated stomach conditions (34.2 mM sodium chloride, 0.1 M hydrochloric acid, pH 1). Comparable observations were made at different stomach-simulating pH values mirroring the physiological situation, such as pH 1, 4, and 5 (Figure 3a–c).

Other preparations, such as pancreatin B 10,000 (60%), pancreatin B 25,000 (54%), pancreatin A 25,000 (47%), and pancreatin F 20,000 (61%) at pH 4, as well as pancreatin B 10,000 (73%), pancreatin B 25,000 (78%), pancreatin A 25,000 (73%), pancreatin I 20,000 (72%), and pancreatin D 25,000 (74%) at pH 5, exhibited substantial release of lipase activity within 15 min (Figure 3b,c).

For pancreatin K preparations, release of lipase activity was rapid after pellets had entered the simulated small intestine (pH 6). After 30 and 60 min at pH 6 (90 and 120 min overall treatment, respectively), pancreatin K preparations exhibited the highest activity compared to other preparations, which was the case in all three described settings. Here, it should be mentioned that these numbers are relative to the actual lipase activity of the respective product. Therefore, a product with large excess activity as defined above might provide a strong activity although the relative activity compared to its maximal activity is low under a given condition.

**Figure 4:**
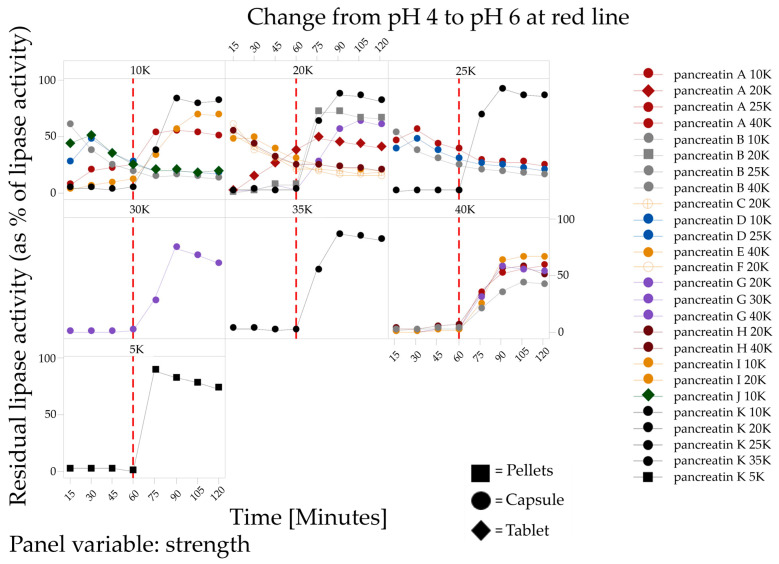
Lipase activity (% of actual maximal activity of the specific PEP) after the indicated time at pH 4 and upon shift to pH 6, represented for individual strengths. The red line indicates the change of pH at 60 min.

At 90 min, pancreatin K 25,000 had 90% of its actual maximum activity within the three measured pH areas. The release from other pancreatin products was comparatively low upon change to pH 6. For example, 30 min after pH adjustment from 5 to 6, pancreatin F 20,000 had 34% activity, pancreatin B 25,000 had 35% activity, pancreatin D 25,000 had 39% activity, and pancreatin A 40,000 had 46% activity. In addition, there are considerable variations in the enzyme release between different strengths of the same brand; for example, between pancreatin H 20,000 and 40,000 in the setting from pH 4 to 6 (Figure 4).

## 3. Discussion

In this in vitro study, multiple formulations of pancreatic enzyme preparations that are available on the German market were assessed and compared for their physical properties, such as particle size, enzyme activity, and enzyme release behavior, to determine their interchangeability.

There is no clinical head-to-head trial with the endpoint clinical efficacy of PERT available. Still, there are indications that the particle size of the pancreatic enzyme preparations might have implications on their efficacy [15,16]. A study conducted in healthy subjects came to the conclusion that spheres with 1.4 ± 0.3 mm in diameter empty at the same rate as a test meal and may therefore be advantageous [17]. Furthermore, 1 mm spheres emptied faster than 2.4 mm or 3.2 mm spheres [17]. Moreover, a study with PEI patients found that lipolytic activity was delayed with microspheres with diameters of 1.8 mm–2.0 mm compared to smaller ones with diameters of 1.0 mm–1.2 mm [18]. Consequently, in a previous study comparing PERT preparations, a particle size of less than 1.7 mm was proposed to be beneficial [12]. The relevance of the particle size was discussed in two letters to the editor in response to the study by Löhr and colleagues [19,20]. However, since several studies have demonstrated a pivotal role of a particle size of 2 mm or less, at this point it seems reasonable to regard a small particle size as beneficial [12].

Furthermore, previous studies have shown that particles bigger than 2.0 mm are retained in the stomach for more than 2 h, potentially resulting in limited efficacy [21]. Thus, products with small particle size exhibit high lipolytic activity and rapid onset of action [15]. In this study, only pancreatin K preparations of all strengths exhibited a FERET Max D[v, 0.5] of less than 1.7 mm, whereas all other PEPs showed a FERET Max D[v, 0.5] of more than 2.5 mm (Table 2). These results confirm previous studies which have shown that pancreatin K preparations are comparatively small [12].

In addition to the particle size, the particle count is an important and likely beneficial property of PEPs, as the distribution of pancreatic enzymes across a large number of particles facilitates efficient and homogenous mixing with the chyme [9]. In the current study, the particle counting revealed that in PEPs with a specific strength, preparations with type I pellets had the largest number of particles (257–314 particles in strength 10,000), followed by type II pellets (74–81 particles in strength 10,000) (Table 3). The least number of particles were found in preparations with mini-tablets (19–30 in strength 10,000) (Table 3). The same overall results were obtained in preparations with a strength of 20,000. In general, as expected, the number of particles increased with strength; however, for type II pellets, this increase was comparably low.

Although rare, it has been shown that high doses of PEPs have been correlated with the development of fibrosing colonopathy [13,22]. Consequently, in 2009 the European Medicines Agency (EMA) recommended to remain below a dose of 10,000 IU lipase/kg body weight in the guidelines on the safety of PEPs [13]. Therefore, all marketed enzyme preparation formulations should at best exhibit 100% label-claimed lipase activity [13]. Historically, several products had a high enzyme excess in order to ensure enzyme activity at the end of shelf life and some products still seem to not comply with the recommendations [13]. It is important that this concern is only relevant to the excess of lipase activity. Additionally, precise compliance with the label claim is a prerequisite for a safe substitution of products. This assessment is particularly important when patients intend to reduce or increase the dose. In the current study, lipase activity was more than 100% of the label claim for all enzyme preparations except for pancreatin K 25,000 (99.1%) (Figure 2). Some preparations (pancreatin B 10,000 and 20,000; pancreatin A 10,000) had more than 120% of label claimed activity. The activities of protease and amylase were higher than the label claim (more than 105%) for all products (Table 4).

An ideal pancreatic enzyme preparation disintegrates immediately once it reaches duodenum (pH 6–7) [12]. Since the pancreatic lipase is acid labile, preparations should not release the enzyme at low pH, which is usually present in the stomach. Therefore, PEPs need to be enterically coated [12]. pH in the gastric region is usually between 1 and 2; however, due to buffering capacity of ingested food, after a meal the pH increases to an average value of around 5 [12,15,23,24]. Thus, an ideal PEP should not release the enzyme within a pH range of 1 to 5.

In our study, no lipase activity was measured for any of the enzyme preparations when incubated at pH 1. Upon increase of the pH to 6, the products generally released active lipase (Figure 3a). This suggests that at pH 1, the enzymes were retained and protected in the pellets or mini-tablets. After incubation at pH 4 and 5, there was minimal lipase activity observed in the case of pancreatin K preparations (<5%) (Figure 3b,c). In contrast, other PEPs showed substantial lipase activity at pH 4. For example, pancreatin B 10,000 and 25,000, pancreatin A 25,000, and pancreatin C 20,000 exhibited lipase activity of >50% at pH 4 (Figure 3b). Consistently, these PEPs, along with others, show strong lipase activity at pH 5, which, transferred to the real world, would mean that enzymes are released before the duodenum is reached. This might be a problem in situations where the gastric pH drops again, since in pH below 4, released pancreatic enzymes become irreversibly inactivated [15]. Correspondingly, PEPs with elevated lipase activity at pH 4 or 5 generally have lower lipase activities at pH 6, compared to PEPs where the enzymes were retained before pH 6 was reached (Figure 3b,c).

Food reaches the jejunum usually after 60 min [12]. Before that, after the chyme has entered the duodenum, the enzymes should become active as soon as possible. In this study, all pancreatin K preparations showed 80% of their maximal lipase activity within 30 min after incubating at pH 6, regardless of the previous condition (Figure 3a–c). As noted before, other preparations, many of which exhibited substantial lipase activity at pH 4 or 5, showed lower lipase activities at pH 6, between 14% and 72% (Figure 3b,c). Further investigation of the underlying reasons for the different enzyme release behavior of the various PEPs was not in the scope of this study. However, it is noticeable that pancreatin K, which is the PEP with the smallest pellet size, has a distinct enzyme release behavior at the change from pH 5 to pH 6, meaning that it retains most of the enzyme activity at pH 5, followed by a fast release at pH 6. In addition to pellet size, different compositions of polymers used in the formulation of the capsule might play a role in the enzyme release behavior of different PEPs. Corresponding investigations might be of interest in future studies.

The results obtained in this study indicating variations in the enzyme release behavior of different PEPs may give an explanation why switching PEPs can be challenging in patients [25].

In response to the study by Löhr and colleagues, it was argued that in patients with PEI, lipase release at pH 5 could be advantageous due to possibly impaired bicarbonate secretion in these patients [20]. In principle, this statement was supported by Löhr in his response to this argument. However, since pancreatic lipase activity is only 10–20% between pH 5 and pH 6, it was concluded that the properties of this enzyme do not allow major lipase activity below pH 6 [20,26]. More recently, lipases from the ciliate *Tetrahymena thermophila* have been characterized, which are highly active within a pH range from pH 2.0 to pH 9.0, highly active in the presence of bile salts, and more stable after incubation in human gastric juice compared to porcine lipase or rizolipase. These enzymes are therefore promising candidates for an improvement in PERT [27]. However, these newly characterized enzymes have not been applied for treatment of human PEI so far. Therefore, in this study, we have compared the porcine preparations available on the German market. The importance of acid-protection or acid-stability of the enzymes has been revealed by the observation that, for a reduction in fecal fat excretion, the respective dosage could be reduced by ¾ when compared to a non-protected preparation [28].

Our study results are in line with previous reports that showed considerable differences between various pancreatic enzyme preparations in terms of their physical properties and enzyme release kinetics [12,15,16]. Importantly, all these in vitro studies have been conducted in environments mimicking the gastroduodenal conditions. Hence, caution is required when extrapolating the results into clinical practice. Although this study is not the first of its kind in comparing different PEPs, it is the most comprehensive one so far with 27 products, and the first one to systematically analyze the particle count of the tested PEPs.

In conclusion, this in vitro study reveals considerable differences in particle sizes, enzyme activities, and enzyme release behaviors (at pH 4, 5 and 6) of the various PEPs on the German market. The differences implicate potential difficulties regarding the interchangeability of these preparations, which may have clinical implications for PEI patients. Importantly, the results obtained during this study were generated in vitro. To assess the response of patients to PERT, it would certainly be interesting to study health parameters in response to treatment with the different PERT preparations tested in this study. A retrospective study of PERT has been conducted with cats, and such an approach might be feasible in human patients as well [29]. A comparison of the two products Kreon and Zenpep, the latter of which is not available in Germany, in a randomized, double-blind, crossover study has shown comparable efficacy in terms of fat absorption for both products [30]. A similar approach for the PERT preparations from the German market, as characterized in this study, would be beneficial.

## 4. Materials and Methods

In this study, the following pancreatic enzyme preparations were analyzed for physical properties and enzyme activity: Pangrol 10,000, 20,000, 25,000, and 40,000 (pancreatin A; Berlin-Chemie, Berlin, Germany); Panzytrat 10,000, 20,000, 25,000, and 40,000 (pancreatin B; Allergan, Dublin, Ireland); Pankreatin Stada 20,000 (pancreatin C; Nordmark, Uetersen, Germany), Pankreatan 10,000, 20,000, and 25,000 (pancreatin D; Nordmark, Uetersen, Germany); Pankreatin 40,000 (pancreatin E; Nordmark, Uetersen, Germany); Pankreatin Mikro 20,000 (pancreatin F; Ratiopharm, Ulm, Germany); Cotazym 20,000, 30,000, and 40,000 (pancreatin G; UCB, Brussels, Belgium or Cheplapharm Arzneimittel, Greifswald, Germany); Ozym 20,000 and 40,000 (pancreatin H; Trommsdorff, Alsdorf, Germany); Pankreatin Laves Micro 10,000 and 20,000 (pancreatin I; Nordmark, Uetersen, Germany); Mezym 10,000 (pancreatin J; Berlin-Chemie, Berlin, Germany); Kreon 5000, 10,000, 20,000, 25,000, and 35,000 (pancreatin K; Abbott Laboratories, North Chicago, IL, USA). Respective batch numbers can be found in Table A1. For products where multiple batches were analyzed, batch 2 was used unless stated otherwise.

### 4.1. Physical Properties

#### 4.1.1. Particle Imaging

Scanning electron microscopy (SEM) was performed on samples from each of the batches to assess their morphology using a Hitachi FlexSEM 1000 (Hitachi, Tokyo, Japan). Samples were mounted on sample stubs (G301P, Agar Scientific, Stansted, UK) using 12 mm carbon tabs (G3347N, Agar Scientific, Stansted, UK). The mounted samples were gold coated (5 nm layer) using a Quorum Q150RS sputter coater (Quorum Technologies, Laughton, UK). Depending on the sample, rising voltages between 5 and 15 kV were used to achieve optimal surface detail. Images were taken with magnifications ×37–×350.

#### 4.1.2. Particle Size Distribution

Particle size analysis was performed using a Sympatec QICPIC (Dynamic Image Analyser), with GRADIS Disperser (Sympatec, Clausthal-Zellerfeld, Germany) and M8 lens. For the analysis of the particle size, the Feret diameter was measured. The Feret diameter is the distance between two parallel tangents of the particle at an arbitrary angle. The Feret diameters for a set of 20 different angles were calculated, and the maximum diameter was selected for FERET Max, which in turn was used for the particle size analysis. Particle sizes were represented at 50th percentiles (FERET Max D[v, 0.5]), at which 10% or 50% of the material is smaller than this. For each batch, three measurements were made (*n* = 3). Results of individual replicates can be found in the Supplementary Materials.

#### 4.1.3. Particle Counting

The contents of all investigated product capsules were imaged using a high resolution Optimax EvoCam (Optimax, Leicestershire, UK) with a field of view of 10 cm^2^. A capsule was emptied onto a clean sheet of paper and gently agitated to disperse the contents. Where the product was not contained within a capsule, particles were dispensed according to dosing instruction using the provided spoons/spatulas. Three samples were evaluated for each batch. These images were analyzed using the image analysis function of the ZenCore software V2.5 (Carl Zeiss, Jena, Germany) to count the capsule contents.

### 4.2. Enzyme Activity and Enzyme Release Kinetics

Enzymatic analysis was performed by measuring the activities of amylase, lipase, and protease for each sample. Furthermore, actual activity was compared against the label claims. In addition, the enzyme preparations were exposed to different pH levels and subsequently, the activity of released amylase, protease, and lipase were measured. The activities for each sample were determined according to the procedures specified by the European Pharmacopoeia (Ph. Eur.) and Löhr et al., 2009 [12]. For these measurements, 20 capsules (or a minimum of 5 g in case of free pellets) were used for testing. Two titrations for the measurement of amylase and lipase and three titrations for the measurement of protease were performed.

#### 4.2.1. Enzyme Activities

Amylase activity was measured using a starch solution as a substrate. The rate of starch hydrolysis with each enzyme preparation was measured and compared to a standard enzyme solution with known activity. For this purpose, amylase reference standard provided by Ph. Eur. was applied. The analysis is based on the reaction of reducing groups, resulting from the hydrolysis, with iodine in alkaline solution and the titration of excess iodine with thiosulfate. The analysis was carried out at 25.0 °C ± 0.1 °C and pH 6.8 in the presence of 0.2 M sodium chloride. The temperature was controlled using a water bath set to 25 °C. The solution temperatures were individually confirmed to be within the allowed range before starting the test, and the bath temperature was monitored throughout, using an Omega Dual Input Digital Thermometer (HH801B, Omega, Deckenpfronn, Germany). The reaction was quenched with 1 M hydrochloric acid, then 0.05 M (0.1 N) iodine solution was added and the samples were alkalized with 0.1 M sodium hydroxide. The samples were left in the dark for >15 min for the iodine reaction to complete. The samples were then acidified with 20% sulfuric acid and the excess iodine was manually titrated using 0.1 M sodium thiosulfate. The readout for the analysis was the volume of sodium thiosulfate used to obtain a color change from purple to colorless.

Lipase activity was assessed using olive oil emulsion as a substrate. The rate of olive oil hydrolysis with each enzyme preparation was measured and compared to a standard enzyme solution with known activity. For this purpose, lipase reference standard provided by Ph. Eur. was applied. The analysis was carried out at 37.0 °C ± 0.5 °C and the pH was maintained at 9.0 using NaOH (0.1 N) for pH-stat titration using the Mettler Toledo T9 Excellence titrator (Mettler Toledo, Greifensee, Switzerland). The olive oil emulsion (OOE) was prepared by combining 10% acacia solution (*w*/*v*) with olive oil and ice. The OOE globule diameter for each olive oil/acacia batch was assessed by microscope and was required to meet the Ph. Eur. criteria of at least 90% of globules < 3 µm and up to 10% with a diameter of 3–10 µm. To run the test, the OOE was combined with a reagent solution (50 mL 8% sodium taurocholate solution, 200 mL tris(hydroxymethyl)aminomethane sodium chloride buffer, and 240 mL water). The test was then performed by adding 1 mL of sample to the substrate mixture and proceeding with a pH-stat titration.

Protease activity was assessed using casein solution as a substrate. The protease digests casein and liberates tyrosine and other amino acids. The amount of tyrosine liberated with each sample solution was determined by UV-Vis spectrometry, measuring absorbance of the filtered solutions at 275 nm wavelength. For this purpose, a Mettler Toledo UV7 UV/Vis spectrophotometer was used. The results were compared to the amount of tyrosine liberated with standard protease solution. The analysis was carried out at 35.0 °C ± 0.5 °C and pH 7.5.

#### 4.2.2. Lipase Activity after Enzyme Release

For assessment of the potential enzyme release from different preparations, the lipase activity upon exposure to defined pH was measured. For this purpose, an in vitro model was used, based on the European Pharmacopoeia disintegration tester, in order to simulate the conditions at the gastro–duodenal transition [12,31]. Comparable enzyme release kinetics experiments on pancreatin products, using a disintegration apparatus, have previously been performed in publications [12,31,32]. The equipment used in this study was in line with Ph. Eur. 2.9.1 with the lipase assay from Pancreas Powder Ph. Eur. Monograph (0350) [33]. The test itself is not specified in the Ph. Eur. For the measurement of enzyme release, an appropriate number of capsules were used to prepare a bulk sample with 80–100 K lipase units on the day of analysis. In phase 1, contents from the sample products were placed in the disintegration apparatus DTG 200i-IS with heater and basket mesh size 2.0 mm (Copley Scientific, Nottingham, UK) and agitated for 60 min at 37.0 °C ± 1 °C, at pH 1, 4, or 5. In phase 2, the pH was adjusted to 6. For adjustment from pH 1 to pH 6, the undissolved portion was separated from the media by suction filtration through a 0.45 µm pore size PES (polyethersulfone) filter. The undissolved portion was then added to 600 mL of sample medium with pH 6 for the phase 2 test. For increasing pH 4 or pH 5 to pH 6, the pH was adjusted with a 4 M sodium hydroxide solution. After adjustment of the pH, the contents were agitated for another 60 min. Four test samples were collected, one every 15 min, to measure the lipase activity. The lipase activity at each time point was measured and presented as percentage of the actual lipase activity of the respective product. The pH was monitored and maintained at the designated pH ± 0.1. pH adjustment was only required for some products where the enteric coating was coming off or a tablet was in the process of disintegrating. These samples went through a short period of pH change and then did not need any further adjustment. Volumes required were usually <500 µL HCl (4 M) or NaOH (4 M). The lipase activity at each timepoint was analyzed using the lipase assay pH-stat method. The ideal conditions for the release of pancreatic enzymes mimicking the gastroduodenal conditions involve no enzyme release during the first 60 min at pH 1, 4, or 5, whereas at pH 6, the enzymes should be released immediately and as entirely as possible.

## 5. Conclusions

Pancreatic enzyme preparations (PEPs) are essential for the survival of patients suffering from pancreatic enzyme insufficiency. The products available in Germany show different size and pH profiles which may influence the intestinal activity, the overall efficacy, and the products’ interchangeability. In this in vitro study, we have systematically analyzed PEP physical properties and pH-dependent activities. Considerable differences between PEPs on the German market were observed, such as variations in particle sizes, particle number, total and declared enzyme activities, and enzyme release behaviors. The duodenal PEP activity is influenced by pH and size-dependent release kinetics, and may be different from the label claim units. These differences suggest limited interchangeability of these preparations, which in turn may have implications for patients with PEI. Importantly, these results were generated in vitro, and it would be interesting to determine the properties of the tested PEPs in humans.

## Figures and Tables

**Table 1:** Distribution of products regarding the three observed presentations.

Presentation	Products
pellet type I	pancreatin K 5000, pancreatin K 10,000, pancreatin K 20,000, pancreatin K 25,000, pancreatin K 35,000
pellet type II	pancreatin A 40,000, pancreatin B 40,000, pancreatin E 40,000, pancreatin G 20,000, pancreatin G 30,000, pancreatin G 40,000, pancreatin H 40,000, pancreatin I 10,000
mini-tablets	pancreatin A 10,000, pancreatin A 25,000, pancreatin B 10,000, pancreatin B 20,000, pancreatin B 25,000, pancreatin C 20,000, pancreatin D 10,000, pancreatin D 20,000, pancreatin D 25,000, pancreatin F 20,000, pancreatin H 20,000, pancreatin I 20,000
tablet	pancreatin A 20,000, pancreatin J 10,000

**Table 2:** Mean Feret Max diameter (*n* = 3) and respective presentation of pancreatic enzyme preparations.

Product	D[v, 0.5]/µm	Presentation
pancreatin A 10,000 batch 1	2632	Mini-Tablet
pancreatin A 10,000 batch 2	2616	Mini-Tablet
pancreatin A 20,000	N/A *	Tablet
pancreatin A 25,000 batch 1	2615	Mini-Tablet
pancreatin A 25,000 batch 2	2617	Mini-Tablet
pancreatin A 40,000	2529	Pellet (type II)
pancreatin B 10,000	2615	Mini-Tablet
pancreatin B 20,000	2615	Mini-Tablet
pancreatin B 25,000 batch 1	2614	Mini-Tablet
pancreatin B 25,000 batch 2	2614	Mini-Tablet
pancreatin B 40,000	2579	Pellet (type II)
pancreatin C 20,000	2612	Mini-Tablet
pancreatin D 10,000	2612	Mini-Tablet
pancreatin D 20,000	2614	Mini-Tablet
pancreatin D 25,000	2612	Mini-Tablet
pancreatin E 40,000	2519	Pellet (type II)
pancreatin F 20,000	2618	Mini-Tablet
pancreatin G 20,000	2601	Pellet (type II)
pancreatin G 30,000	2547	Pellet (type II)
pancreatin G 40,000	2468	Pellet (type II)
pancreatin H 20,000	2616	Mini-Tablet
pancreatin H 40,000	2528	Pellet (type II)
pancreatin I 10,000	1979	Pellet (type II)
pancreatin I 20,000	2611	Mini-Tablet
pancreatin J 10,000	N/A **	Tablet
pancreatin K 5000	1449	Pellet (type I)
pancreatin K 10,000 batch 1	1541	Pellet (type I)
pancreatin K 10,000 batch 2	1566	Pellet (type I)
pancreatin K 10,000 batch 3	1537	Pellet (type I)
pancreatin K 20,000	1608	Pellet (type I)
pancreatin K 25,000 batch 1	1588	Pellet (type I)
pancreatin K 25,000 batch 2	1608	Pellet (type I)
pancreatin K 25,000 batch 3	1531	Pellet (type I)
pancreatin K 35,000	1542	Pellet (type I)

* tablet size = 11.39 mm, measured using digital calipers. ** tablet size = 10.31 mm, measured using digital calipers.

**Table 3:** Minimum and maximum of counted particles per unit dose sorted by strength and presentation.

		Strength						
		5K	10K	20K	25K	30K	35K	40K
**Quantity**	Type I pellets	124–132	257–314	387–414	384–608	-	726–765	-
	Type II pellets	-	74–81	60–62	-	84–93	-	92–145
	Mini-tablets	-	19–30	42–50	43–61	-	-	-

**Table 4:** Comparison of activities of lipase, amylase, and protease as labelled, measured per unit dose, and calculated percentage of the label claim.

	Lipase Activity			Amylase Activity			Protease Activity		
Product	Labelled Lipase Activity/Dose Unit	Lipase Assay/Unit Dose	Lipase % of Label Claim	Labelled Amylase Activity/Dose Unit	Amylase Assay/Unit Dose	Amylase % of Label Claim	Labelled Protease Activity/Dose Unit	Protease Assay/Unit Dose	Protease % of Label Claim
pancreatin A 10,000	10,000	12,444	**124.4**	9000	11,728	**130.3**	500	633	**126.6**
pancreatin A 20,000	20,000	20,900	**104.5**	12,000	19,244	**160.4**	900	1290	**143.3**
pancreatin A 25,000	25,000	29,268	**117.1**	22,500	28,363	**126.1**	1250	1529	**122.3**
pancreatin A 40,000	40,000	42,784	**107.0**	25,000	33,285	**133.1**	1500	1735	**115.7**
pancreatin B 10,000	10,000	12,583	**125.8**	9000	11,016	**122.4**	500	599	**119.7**
pancreatin B 20,000	20,000	29,615	**148.1**	18,000	21,172	**117.6**	1000	1109	**110.9**
pancreatin B 25,000	25,000	27,929	**111.7**	15,000	20,922	**139.5**	800	1012	**126.5**
pancreatin B 40,000	40,000	44,413	**111.0**	25,000	30,131	**120.5**	1500	1761	**117.4**
pancreatin C 20,000	20,000	21,299	**106.5**	15,000	18,362	**122.4**	900	1057	**117.5**
pancreatin D 10,000	10,000	10,516	**105.2**	7500	10,030	**133.7**	450	504	**112.0**
pancreatin D 25,000	25,000	26,442	**105.8**	18,750	22,667	**120.9**	1125	1207	**107.3**
pancreatin E 40,000	40,000	41,555	**103.9**	25,000	28,979	**115.9**	1500	1742	**116.1**
pancreatin F 20,000	20,000	21,572	**107.9**	15,000	20,545	**137.0**	900	997	**110.8**
pancreatin G 20,000	20,000	22,327	**111.6**	14,500	20,292	**139.9**	850	1041	**122.5**
pancreatin G 30,000	30,000	33,283	**110.9**	21,750	30,033	**138.1**	1275	1717	**134.6**
pancreatin G 40,000	40,000	46,081	**115.2**	25,000	29,655	**118.6**	1500	1601	**106.7**
pancreatin H 20,000	20,000	21,428	**107.1**	15,000	20,609	**137.4**	900	1060	**117.8**
pancreatin H 40,000	40,000	44,575	**111.4**	25,000	33,539	**134.2**	1500	1667	**111.1**
pancreatin I 10,000	10,000	10,648	**106.5**	7250	9665	**133.3**	425	567	**133.5**
pancreatin I 20,000	20,000	20,634	**103.2**	15,000	19,216	**128.1**	900	987	**109.7**
pancreatin J 10,000	10,000	11,466	**114.7**	7500	9213	**122.8**	375	506	**135.0**
pancreatin K 5000	5000	5287	**105.7**	3600	5649	**156.9**	200	330	**165.0**
pancreatin K 10,000	10,000	11,037	**110.4**	8000	12,137	**151.7**	600	790	**131.7**
pancreatin K 20,000	20,000	20,368	**101.8**	16,000	23,967	**149.8**	1200	1556	**129.7**
pancreatin K 25,000	25,000	24,785	**99.1**	18,000	25,975	**144.3**	1000	1477	**147.7**
pancreatin K 35,000	35,000	37,101	**106.0**	25,200	35,906	**142.5**	1400	2090	**149.2**

## Data Availability

Data is contained within the article and Supplementary Materials.

## Supplementary Materials

The following supporting information can be downloaded at: https://www.mdpi.com/article/10.3390/ph17050552/s1.

## Appendix A

**Table A1:** Batch numbers of investigated pancreatic enzyme preparations.

Product	Marketed Name	Supplier	Batch Number
pancreatin A 10,000	Pangrol	Berlin-Chemie	Batch 1: 83147D Batch 2: 94166E
pancreatin A 20,000	Pangrol	Berlin-Chemie	92027A
pancreatin A 25,000	Pangrol	Berlin-Chemie	Batch 1: 84231E Batch 2: 93255H
pancreatin A 40,000	Pangrol	Berlin-Chemie	92019
pancreatin B 10,000	Panzytrat	Allergan	337801
pancreatin B 20,000	Panzytrat	Allergan	358001
pancreatin B 25,000	Panzytrat	Allergan	Batch 1: 412801 Batch 2: 413201
pancreatin B 40,000	Panzytrat	Allergan	670501
pancreatin C 20,000	Pankreatin Stada	Nordmark Pharma	92238
pancreatin D 10,000	Pankreatan	Nordmark Pharma	321301
pancreatin D 20,000	Pankreatan	Nordmark Pharma	501201
pancreatin D 25,000	Pankreatan	Nordmark Pharma	323101
pancreatin E 40,000	Pankreatin	Nordmark Pharma	672401
pancreatin F 20,000	Pankreatin Mikro	Ratiopharm	321401
pancreatin G 20,000	Cotazym	UCB o. Cheplapharm	507401
pancreatin G 30,000	Cotazym	UCB o. Cheplapharm	507701
pancreatin G 40,000	Cotazym	UCB o. Cheplapharm	659101
pancreatin H 20,000	Ozym	Trommsdorff	N001
pancreatin H 40,000	Ozym	Trommsdorff	N002
pancreatin I 10,000	Pankreatin Laves Mikro	Nordmark Pharma	012501
pancreatin I 20,000	Pankreatin Laves Mikro	Nordmark Pharma	319301
pancreatin J 10,000	Mezym	Berlin-Chemie	98013
pancreatin K 5000	Kreon	Abbott Laboratories	59042
pancreatin K 10,000	Kreon	Abbott Laboratories	Batch 1: 57797 Batch 2: 58519 Batch 3: 57234
pancreatin K 20,000	Kreon	Abbott Laboratories	58845
pancreatin K 25,000	Kreon	Abbott Laboratories	Batch 1: 58259 Batch 2: 58888 Batch 3: 57467
pancreatin K 35,000	Kreon	Abbott Laboratories	59016
