# Supplementary material for: Comparative Investigation of pH–Dependent Availability of Pancreatic Enzyme Preparations In Vitro
Source: Pharmaceuticals (Basel). 2024 Apr 25;17(5):552. doi: 10.3390/ph17050552 (PMC11123882; doi:10.3390/ph17050552)
Supplement: Supplementary file 1 [file pharmaceuticals-17-00552-s001.zip › pharmaceuticals-2908685-supplementary.pdf]

	MYLAN GLOBAL RESPIRATORY GROUP
	**Part 1 - Physical characterisation of Kreon microspheres and its German competitor products**
	**Version:** 1.0

TABLE OF CONTENTS

Table of Contents 1
Introduction 2
Equipment and Experimental procedure 2
Batches and Materials 3
Results 4
1.1 Sample Presentation 4
1.2 Particle Size 6
1.3 Particle Counting 19
Conclusions 23
Appendix 24

Disclaimer: Confidential, personal or proprietary information and proprietary images have been redacted from the original report.

Effective Date: 3/15/2021 5:13:06 AM

Effective ver 1.0

INTRODUCTION

Creon® (Kreon) is a pancreatic enzyme replacement therapy prescribed in response to endocrine pancreatic insufficiency (EPI). This condition is typically a result of cystic fibrosis, pancreatitis, a pancreatectomy or other pancreatic function impacting conditions. The therapy replaces protease, amylase and lipase, enzymes that would otherwise be produced by a healthy pancreas.

The active ingredient in the formulation, pancreatin, is derived from healthy porcine pancreases. These are formulated into micro pellets, cylindrical in shape, approximately 1mm in diameter and 2mm in length. These pellets are enteric coated so that the enzymes are released in the duodenum. The final stage of the pellet formulation is to encapsulate them in a gelatine capsule.

Particle size analysis on the pellets of Creon® and its German competitor products was performed to compare their particle size distribution to support ongoing marketing and educational initiatives and legal disputes. In addition, scanning electron microscopy (SEM) was performed on samples from each of the batches to assess their morphology.

EQUIPMENT AND EXPERIMENTAL PROCEDURE

QICPIC Methodology
Analysis was performed using a Sympatec QICPIC (Dynamic Image Analyser), with GRADIS Disperser and M8 lens. The QICPIC, used to collect particle size and shape data, disperses samples in a fluid stream and individual 2D particle images are captured by high speed photography. The QICPIC uses rear illumination with a visible pulsed light source that has an exposure time of 1ns to minimise motion blur.
The diameter of a circle of equal projection area (EQPC), Feret Min and Feret Max were selected for the evaluation modes. EQPC is the diameter of a circle that has the same area as the projection area of the particle. It is widely used for the evaluation of particles sizes from the projection area A of a non-spherical particle. Feret Diameter is defined as the distance between two parallel tangents of the particle at an arbitrary angle. The Feret diameters for a sufficient number of angles are calculated, and for Feret Max the maximum angle is selected and for Feret Min the minimum angle is selected.
If a particle has an irregular shape, the Feret diameter varies more than with regularly shaped particles. The Feret Min diameter is always smaller, and the Feret Max diameter is always larger, than the diameter of the equivalent circle (EQPC).

SEM Methodology
Samples were mounted on samples stubs (G301P, Agar Scientific) using 12mm carbon tabs (G3347N, 170301, Agar Scientific). The mounted samples were gold coated (5nm layer) using Quorum Q150RS sputter coater. Depending on the sample, accelerating voltages between 5 and 15 kV were used to achieve optimal surface detail. Auto-focus and auto-contrast will be performed prior to the collection of each image, with manual optimisation performed as required. Images were collected at magnifications x37 – x350.

Effective Date: 3/15/2021 5:13:06 AM

Effective ver 1.0

Analysis was performed using Hitachi FlexSEM (Scanning Electron Microscope) and Quorum sputter coater.

BATCHES AND MATERIALS

The following batches were analysed:

Supplier	Material	Batch	Strength
**Abbott Laboratories GmbH**	**Kreon**	59042	5k
		57797	10K
		58519	10K
		57234	10K
		58845	20K
		58259	25K
		58888	25K
		57467	25K
		59016	35K
**Allergan**	**Panzytrat**	337801	10K
		358001	20K
		412801	25K
		413201	25K
		670501	40K
**Berlin-Chemie AG**	**Pangrol**	83147D	10K
		94166E	10K
		92027A	20K
		84231E	25K
		93255H	25K
		92019	40K
**Berlin-Chemie AG**	**Mezym**	98013	10K
**Nordmark**	**PANKREAT.STADA ALI**	92238	20K
	**PANKREATAN (G-O)**	321301	10K
		501201	20K
		323101	25K
	**PANKREATIN LVS**	012501	10K
		319301	20K
	**PANKREATIN**	672401	40K
**Ratiopharm**	**PANKREAT MIKRO**	321401	20K
**Trommsdorff GmbH & Co. KG**	**OZYM**	N001	20K
		N002	40K
**UCB**	**COTAZYM**	507401	20K
		507701	30K
		659101	40K

*Batches are in tablet form, imaged using light microscope and particle size measured using digital callipers.

Effective Date: 3/15/2021 5:13:06 AM

Effective ver 1.0

RESULTS

1.1 SAMPLE PRESENTATION

Three distinct sample presentations were identified during the SEM analysis of the products. These have been labelled as pellets (type I and II) and mini-tablets.

Type I pellets are the smallest of the three presentations, cylindrical in shape, approximately 0.9mm in diameter and varying in length between 1mm and 2.5mm. The diameter tends to be very regular, however the length tends to be more variable. The surface of the type I pellets is generally smooth with very straight sides, with a flat top or bottom, although a significant fraction of type 1 pellets has been observed with slightly convex ends or angled. Type I pellets are unique to Kreon products.

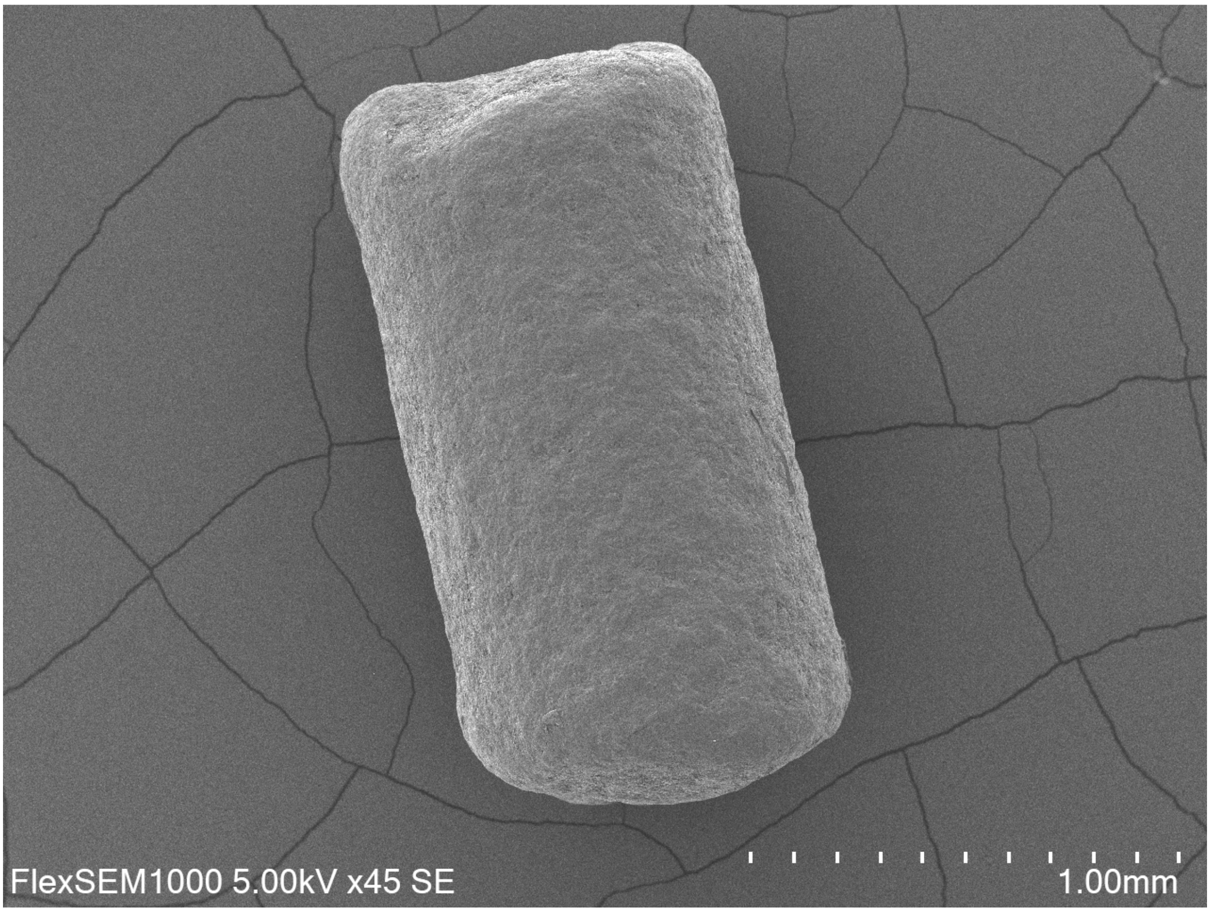

Figure S1: Example of Kreon type I pellets, Batch 58519, 10K Strength

Type II pellets are similar in shape to type I pellets, however generally larger in all orientations and less regular in size and shape. They are also cylindrical, approximately 1.2mm to 1.5mm in diameter and varying in length between 1.2mm and 3.0mm. They tend to have a less regular surface, sometimes with protruding “nodules”. Examples of type II pellets have been observed in batches from multiple vendors, for both mid and high strength products, although more commonly in higher strength products (e.g. 40K).

Effective Date: 3/15/2021 5:13:06 AM

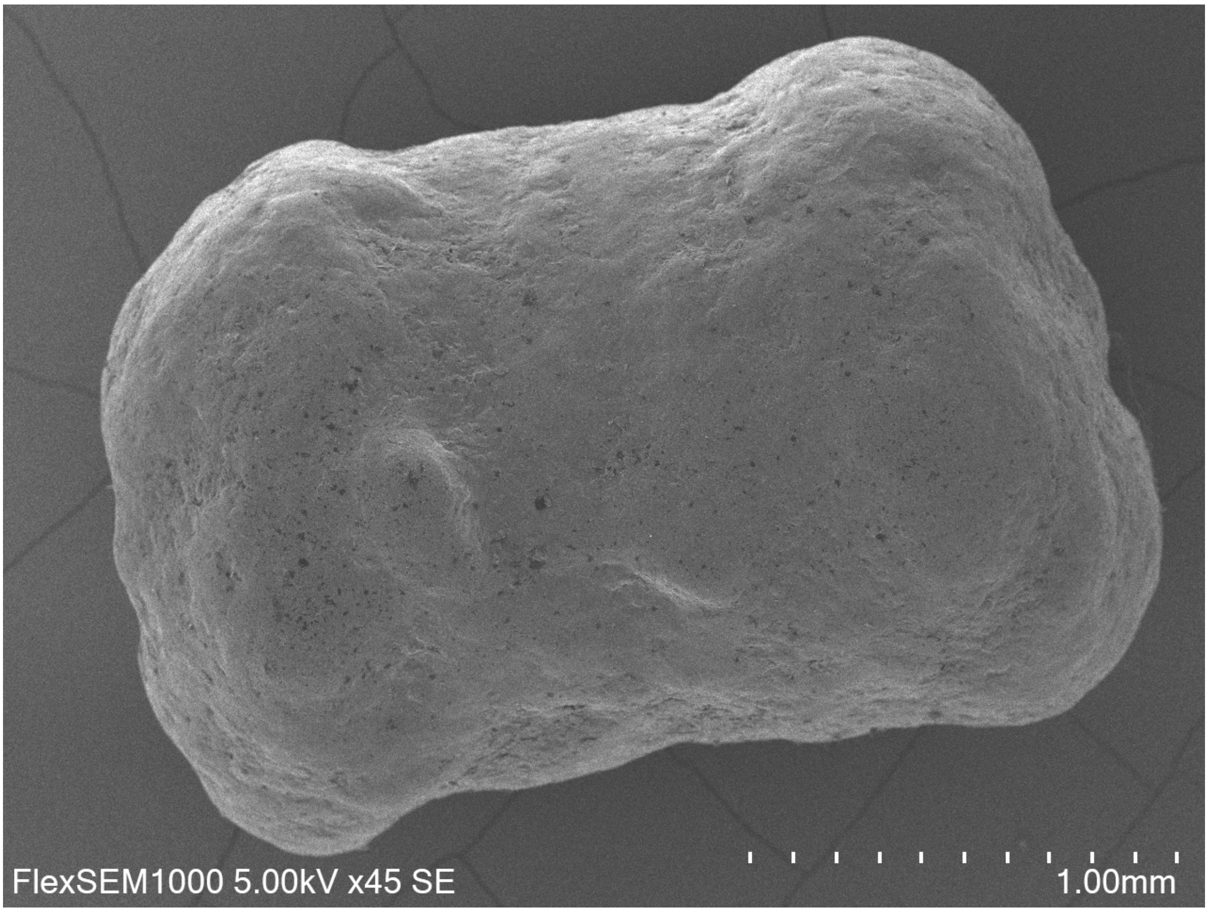

Figure S2: Example of type II pellet Presentation, Cotazym, Batch 507701, 30K Strength

Mini-Tablets (MT) are the largest of the three presentations, shaped like a cylinder with a domed top and bottom. They are approximately 2.2mm in their longest orientation (diagonally, top corner to bottom opposite corner, across the cylindrical centre) and marginally smaller end to end (domed peak to domed peak) and in diameter. They have a smooth surface and are very regular in size and shape. Examples of MT have been observed in batches from multiple vendors, for both low and mid strength products, although that are more commonly in mid strength products.

Effective Date: 3/15/2021 5:13:06 AM

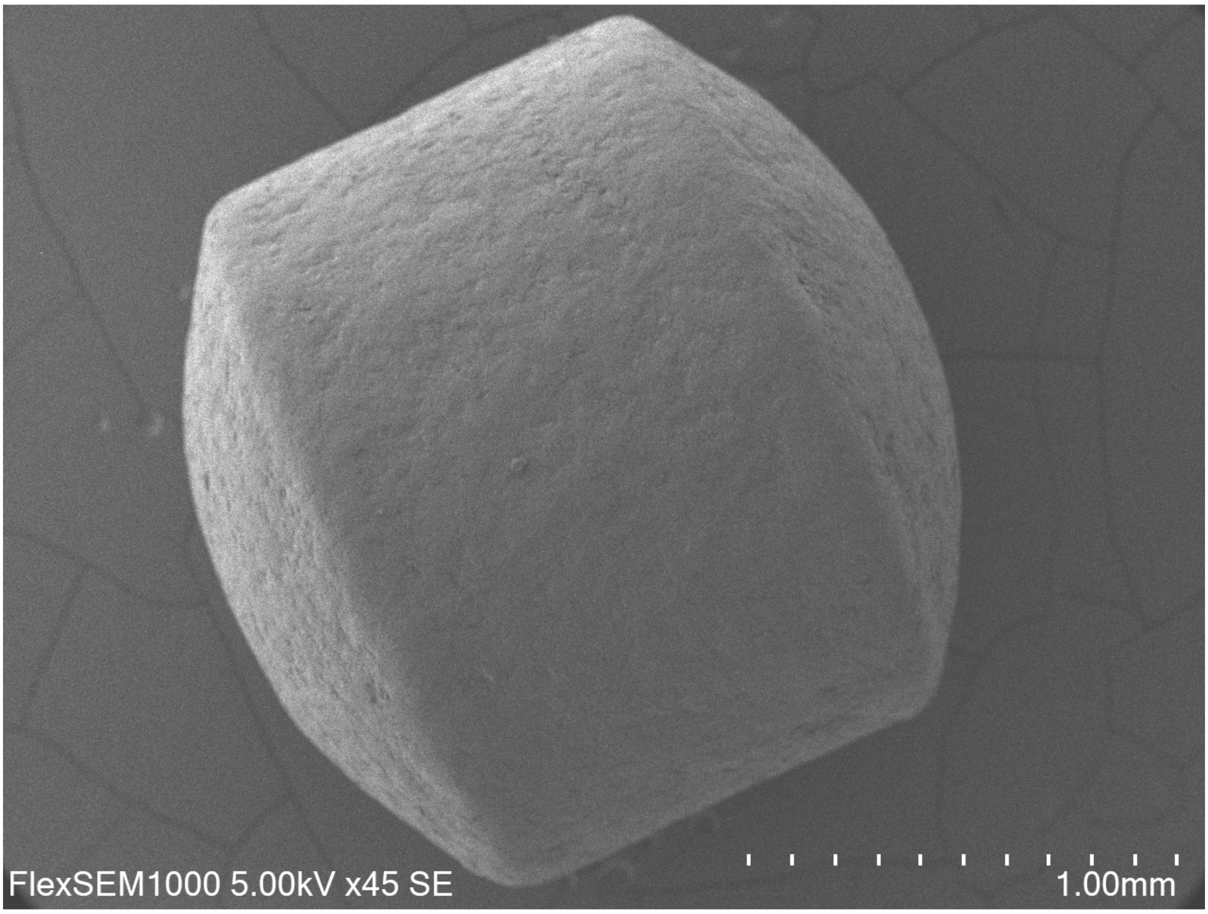

Figure S3: Example of Mini-Tablet presentation. Pankreatin, Batch 319301, 20K Strength.

A summary of batches observed with specific presentations can be found in Table S1, Table S2 and Table S3.

1.2 PARTICLE SIZE

All products were analysed as per MGR-METH-859063 v1 (validated internal method). Mean results are reported in Table S1 – Table S3 below for all batches, using the Feret Max, Feret Min and EQPC evaluation modes.

Table S1. Mean (n=3) Feret Max

Product	Batch	Strength	D[v,0.1] µm	D[v,0.5] µm	D[v,0.9] µm	Presentation
Cotazym	507401	20k	1952	2601	3525	Pellet (type II)
Cotazym	507701	30k	1925	2547	3422	Pellet (type II)
Cotazym	659101	40k	1886	2468	3360	Pellet (type II)

Effective Date: 3/15/2021 5:13:06 AM

Creon	57467	25k	1173	1588	2246	Pellet (type I)
Creon	57797	10k	1131	1566	2225	Pellet (type I)
Creon	58845	20k	1179	1608	2222	Pellet (type I)
Creon	58519	10k	1100	1537	2219	Pellet (type I)
Creon	57234	10k	1116	1541	2191	Pellet (type I)
Creon	58888	25k	1179	1608	2170	Pellet (type I)
Creon	59016	35k	1131	1542	2167	Pellet (type I)
Creon	58259	25k	1122	1531	2161	Pellet (type I)
Creon	59042	5K	1097	1449	2014	Pellet (type I)
Ozym	N002	40k	1917	2528	3402	Pellet (type II)
Ozym	N001	20k	2345	2616	2886	Mini-Tablet
Mezym	98013	10k	*N/A	*N/A	*N/A	Tablet
Pangrol	92019	40k	1917	2529	3405	Pellet (type II)
Pangrol	94166E	10k	2354	2632	2910	Mini-Tablet
Pangrol	84231E	25k	2338	2615	2892	Mini-Tablet
Pangrol	83147D	10k	2350	2616	2883	Mini-Tablet
Pangrol	93255H	25k	2352	2617	2883	Mini-Tablet
Pangrol	92027A	20k	**N/A	**N/A	**N/A	Tablet
Pankreatan	321301	10k	2343	2612	2882	Mini-Tablet
Pankreatan	501201	20k	2345	2614	2882	Mini-Tablet
Pankreatan	323101	25k	2343	2612	2882	Mini-Tablet
Pankreatin	672401	40k	1909	2519	3415	Pellet (type II)
Pankreatin Laves Mikro	319301	20k	2340	2611	2882	Mini-Tablet
Pankreatin Laves Mikro	012501	10k	1467	1979	2667	Pellet (type II)
Pankreatin Mikro	321401	20k	2343	2618	2892	Mini-Tablet
Pankreatin Stada	92238	20k	2342	2612	2882	Mini-Tablet
Panzytrat	670501	40k	1940	2579	3476	Pellet (type II)
Panzytrat	413201	25k	2341	2614	2886	Mini-Tablet
Panzytrat	337801	10k	2343	2615	2887	Mini-Tablet
Panzytrat	358001	20k	2343	2615	2886	Mini-Tablet
Panzytrat	412801	25k	2345	2614	2882	Mini-Tablet

*Tablet size = 10.31 mm

**Tablet size = 11.39 mm

Effective Date: 3/15/2021 5:13:06 AM

Effective ver 1.0

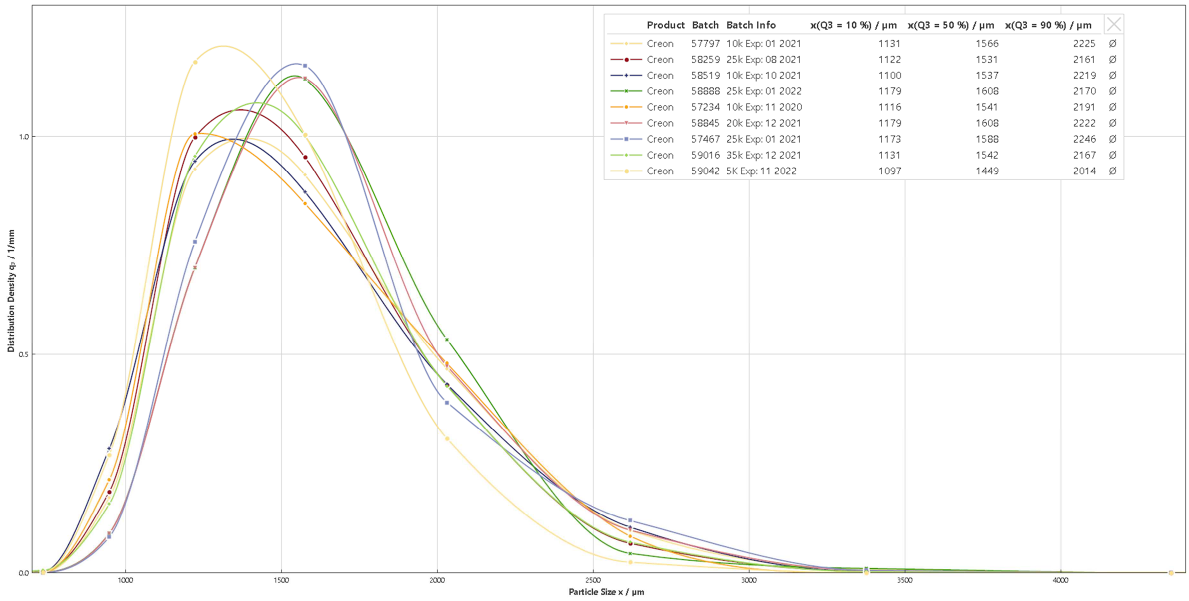

Figure S1. Pellet (type I) – Feret Max

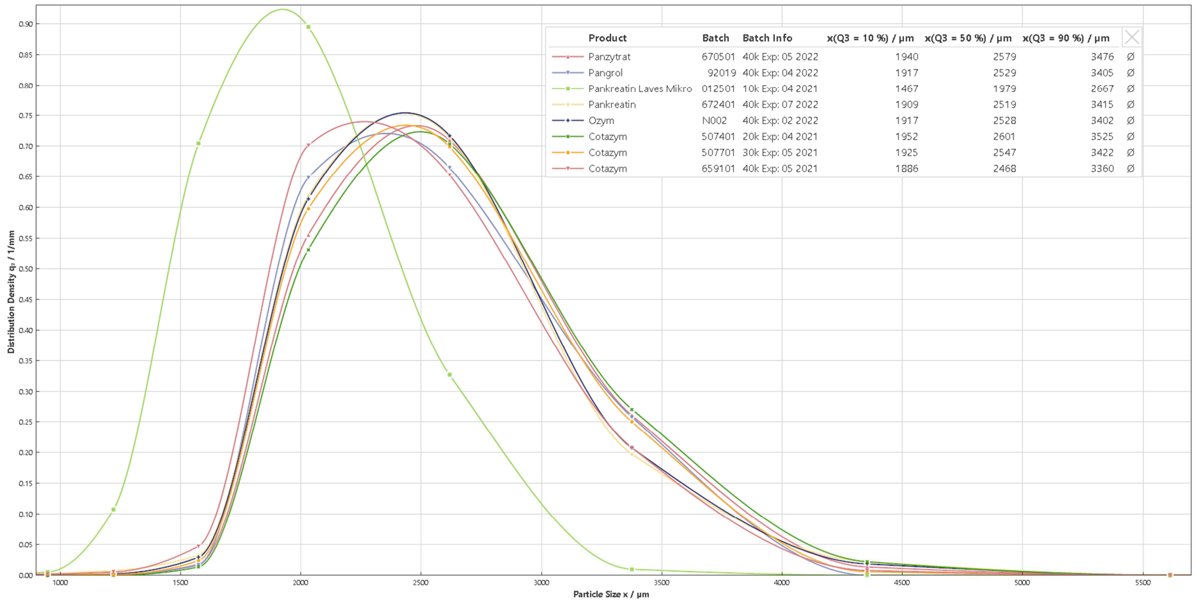

Figure S2. Pellet (type II) – Feret Max

Effective Date: 3/15/2021 5:13:06 AM

Effective ver 1.0

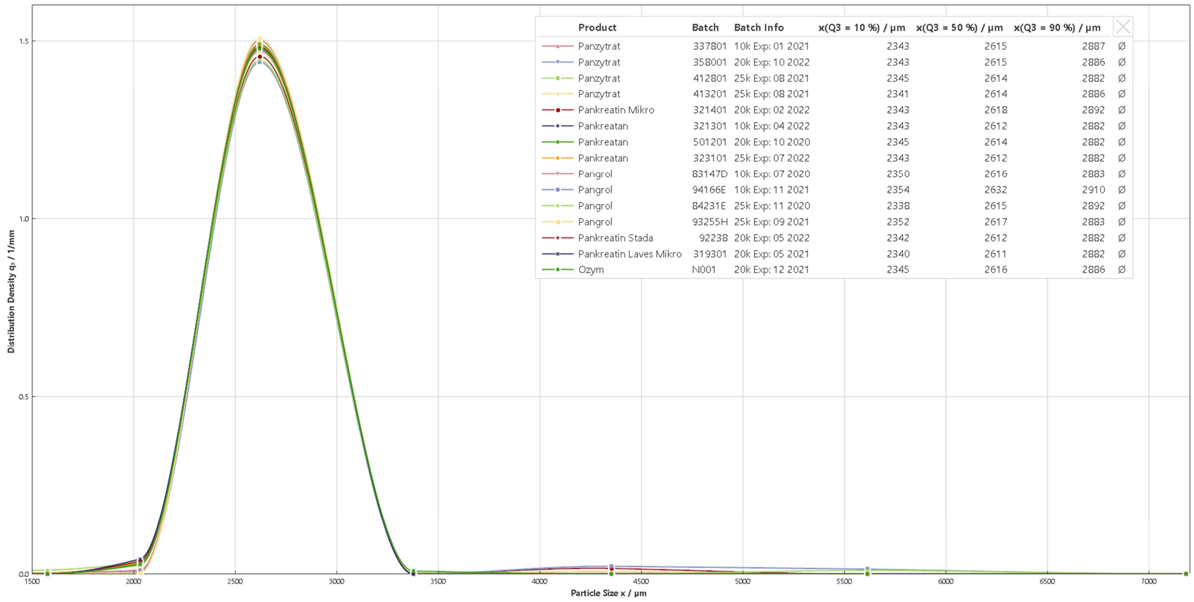

Figure S3. Mini-tablet – Feret Max

Effective Date: 3/15/2021 5:13:06 AM

Effective ver 1.0

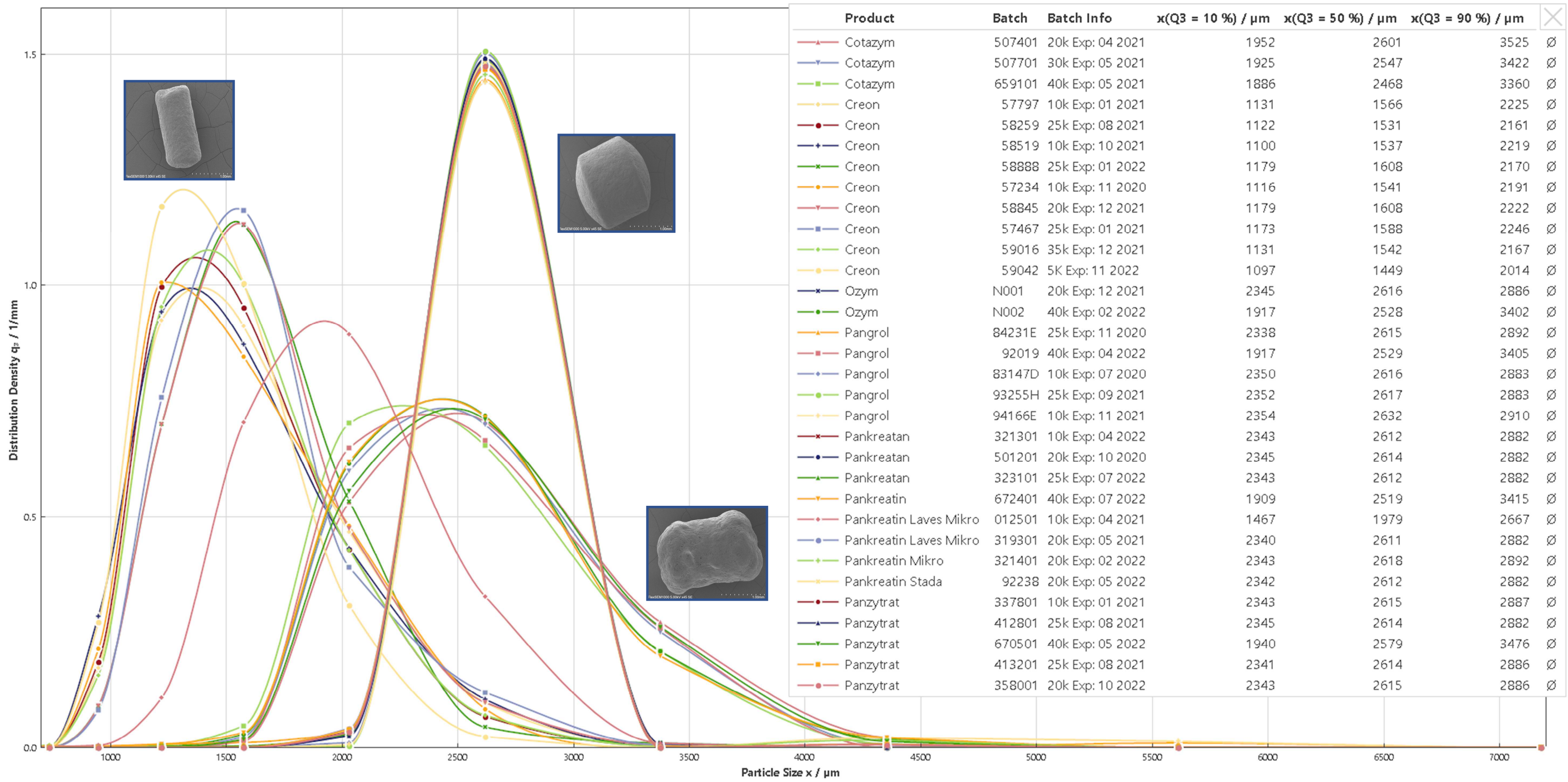

Product	Batch	Batch Info	x(Q3 = 10 %) / µm	x(Q3 = 50 %) / µm	x(Q3 = 90 %) / µm	
Cotazym	507401	20k Exp: 04 2021	1952	2601	3525	Ø
Cotazym	507701	30k Exp: 05 2021	1925	2547	3422	Ø
Cotazym	659101	40k Exp: 05 2021	1886	2468	3360	Ø
Creon	57797	10k Exp: 01 2021	1131	1566	2225	Ø
Creon	58259	25k Exp: 08 2021	1122	1531	2161	Ø
Creon	58519	10k Exp: 10 2021	1100	1537	2219	Ø
Creon	58888	25k Exp: 01 2022	1179	1608	2170	Ø
Creon	57234	10k Exp: 11 2020	1116	1541	2191	Ø
Creon	58845	20k Exp: 12 2021	1179	1608	2222	Ø
Creon	57467	25k Exp: 01 2021	1173	1588	2246	Ø
Creon	59016	35k Exp: 12 2021	1131	1542	2167	Ø
Creon	59042	5K Exp: 11 2022	1097	1449	2014	Ø
Ozym	N001	20k Exp: 12 2021	2345	2616	2886	Ø
Ozym	N002	40k Exp: 02 2022	1917	2528	3402	Ø
Pangrol	84231E	25k Exp: 11 2020	2338	2615	2892	Ø
Pangrol	92019	40k Exp: 04 2022	1917	2529	3405	Ø
Pangrol	83147D	10k Exp: 07 2020	2350	2616	2883	Ø
Pangrol	93255H	25k Exp: 09 2021	2352	2617	2883	Ø
Pangrol	94166E	10k Exp: 11 2021	2354	2632	2910	Ø
Pankreatan	321301	10k Exp: 04 2022	2343	2612	2882	Ø
Pankreatan	501201	20k Exp: 10 2020	2345	2614	2882	Ø
Pankreatan	323101	25k Exp: 07 2022	2343	2612	2882	Ø
Pankreatin	672401	40k Exp: 07 2022	1909	2519	3415	Ø
Pankreatin Laves Mikro	012501	10k Exp: 04 2021	1467	1979	2667	Ø
Pankreatin Laves Mikro	319301	20k Exp: 05 2021	2340	2611	2882	Ø
Pankreatin Mikro	321401	20k Exp: 02 2022	2343	2618	2892	Ø
Pankreatin Stada	92238	20k Exp: 05 2022	2342	2612	2882	Ø
Panzytrat	337801	10k Exp: 01 2021	2343	2615	2887	Ø
Panzytrat	412801	25k Exp: 08 2021	2345	2614	2882	Ø
Panzytrat	670501	40k Exp: 05 2022	1940	2579	3476	Ø
Panzytrat	413201	25k Exp: 08 2021	2341	2614	2886	Ø
Panzytrat	358001	20k Exp: 10 2022	2343	2615	2886	Ø

Figure S4. All Batches – Feret Max

Effective Date: 3/15/2021 5:13:06 AM

Effective ver 1.0

Table S2. Mean (n=3) Feret Min

Product	Batch	Strength	D[v,0.1] µm	D[v,0.5] µm	D[v,0.9] µm	Presentation
Cotazym	507401	20k	1453	1771	2187	Pellet (type II)
Cotazym	507701	30k	1441	1721	2169	Pellet (type II)
Cotazym	659101	40k	1425	1656	2116	Pellet (type II)
Creon	57797	10k	863	1042	1448	Pellet (type I)
Creon	58259	25k	857	995	1388	Pellet (type I)
Creon	58519	10k	849	1043	1374	Pellet (type I)
Creon	58888	25k	894	1152	1356	Pellet (type I)
Creon	57234	10k	856	994	1438	Pellet (type I)
Creon	58845	20k	908	1176	1363	Pellet (type I)
Creon	57467	25k	928	1196	1392	Pellet (type I)
Creon	59016	35k	866	1041	1357	Pellet (type I)
Creon	59042	5K	846	948	1049	Pellet (type I)
Ozym	N001	20k	1825	2031	2237	Mini-Tablet
Ozym	N002	40k	1446	1748	2179	Pellet (Type II)
Mezym	98013	10k	*N/A	*N/A	*N/A	Tablet
Pangrol	84231E	25k	1820	2030	2241	Mini-Tablet
Pangrol	92019	40k	1437	1697	2153	Pellet (type II)
Pangrol	83147D	10k	1825	2031	2236	Mini-Tablet
Pangrol	93255H	25k	1824	2031	2237	Mini-Tablet
Pangrol	94166E	10k	1826	2041	2255	Mini-Tablet
Pangrol	92027A	20k	**N/A	**N/A	**N/A	Tablet
Pankreatan	321301	10k	1822	2029	2236	Mini-Tablet
Pankreatan	501201	20k	1825	2030	2236	Mini-Tablet
Pankreatan	323101	25k	1824	2030	2236	Mini-Tablet
Pankreatin	672401	40k	1430	1680	2150	Pellet (type II)
Pankreatin Laves Mikro	012501	10k	1097	1253	1571	Pellet (type II)
Pankreatin Laves Mikro	319301	20k	1825	2030	2236	Mini-Tablet
Pankreatin Mikro	321401	20k	1825	2031	2238	Mini-Tablet
Pankreatin Stada	92238	20k	1823	2030	2236	Mini-Tablet
Panzytrat	337801	10k	1825	2031	2237	Mini-Tablet
Panzytrat	412801	25k	1825	2030	2236	Mini-Tablet
Panzytrat	670501	40k	1441	1719	2171	Pellet (type II)
Panzytrat	413201	25k	1824	2031	2237	Mini-Tablet
Panzytrat	358001	20k	1825	2032	2238	Mini-Tablet

*Tablet size = 10.31 mm
**Tablet size = 11.39 mm

Effective Date: 3/15/2021 5:13:06 AM

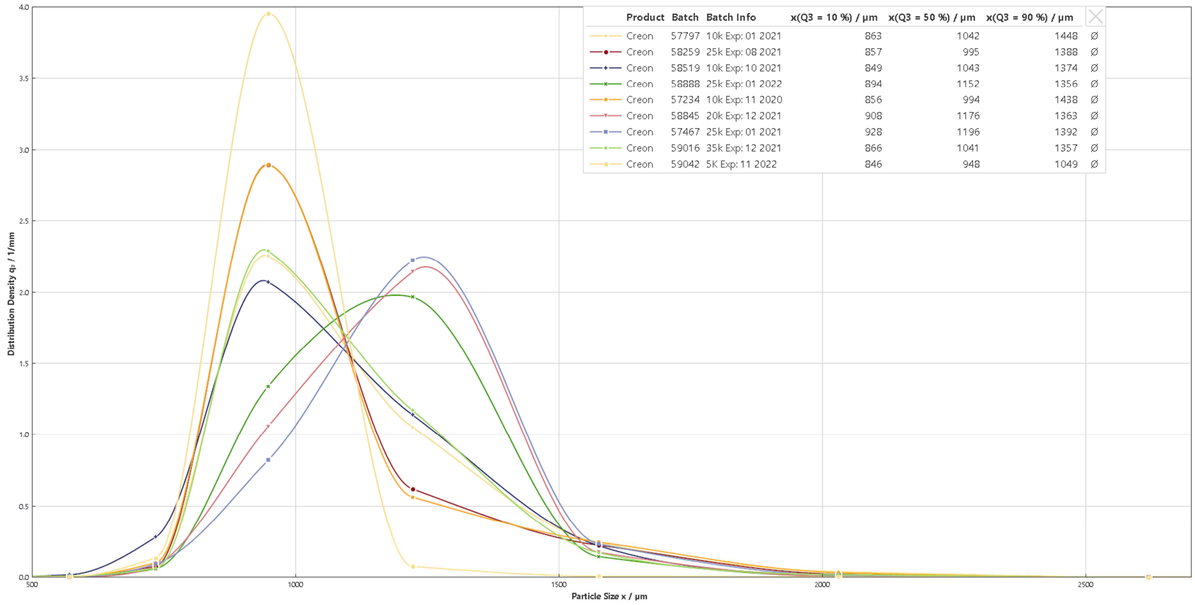

Figure S5. Pellet (type I) - Feret min

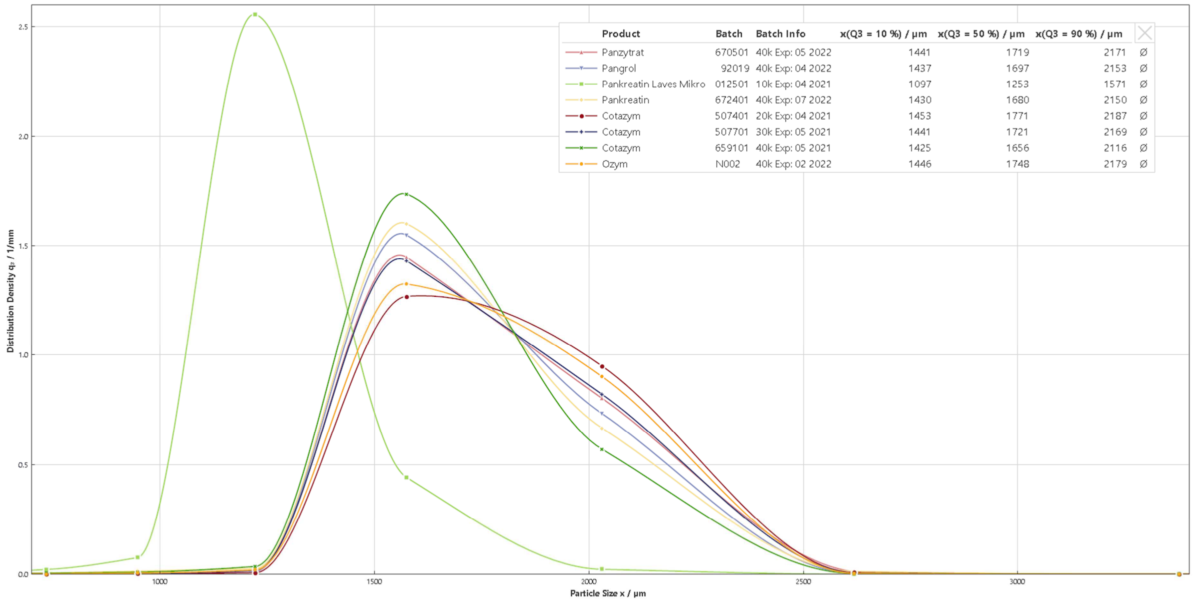

Figure S6. Pellet (type II) - Feret Min

Effective Date: 3/15/2021 5:13:06 AM

Effective ver 1.0

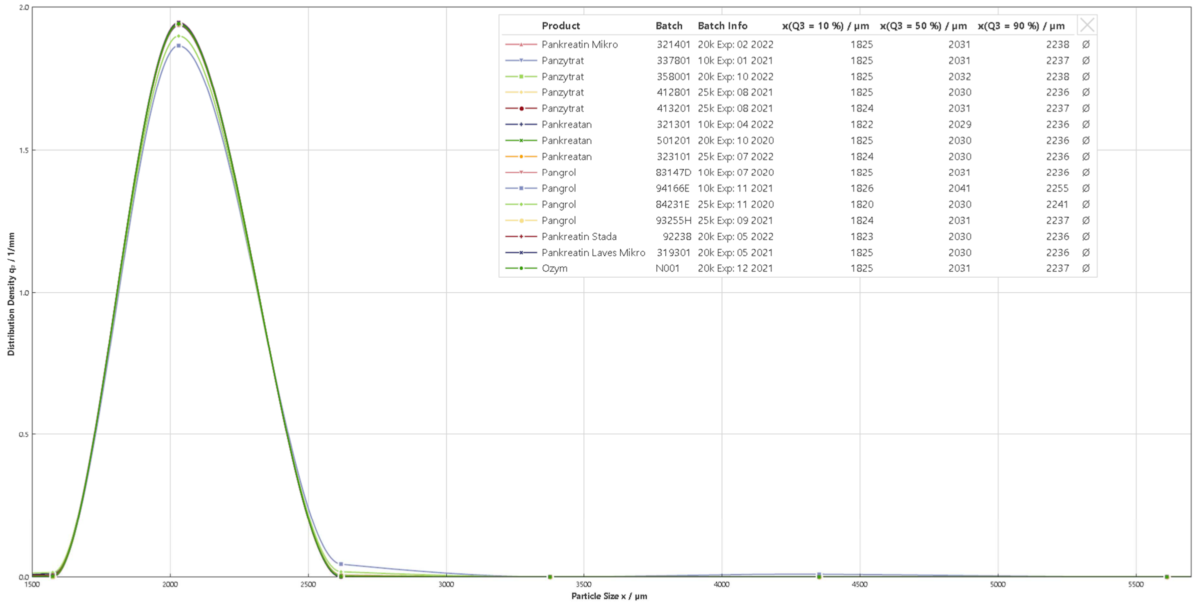

Figure S7. Mini-Tablet - Feret Min

Effective Date: 3/15/2021 5:13:06 AM

Effective ver 1.0

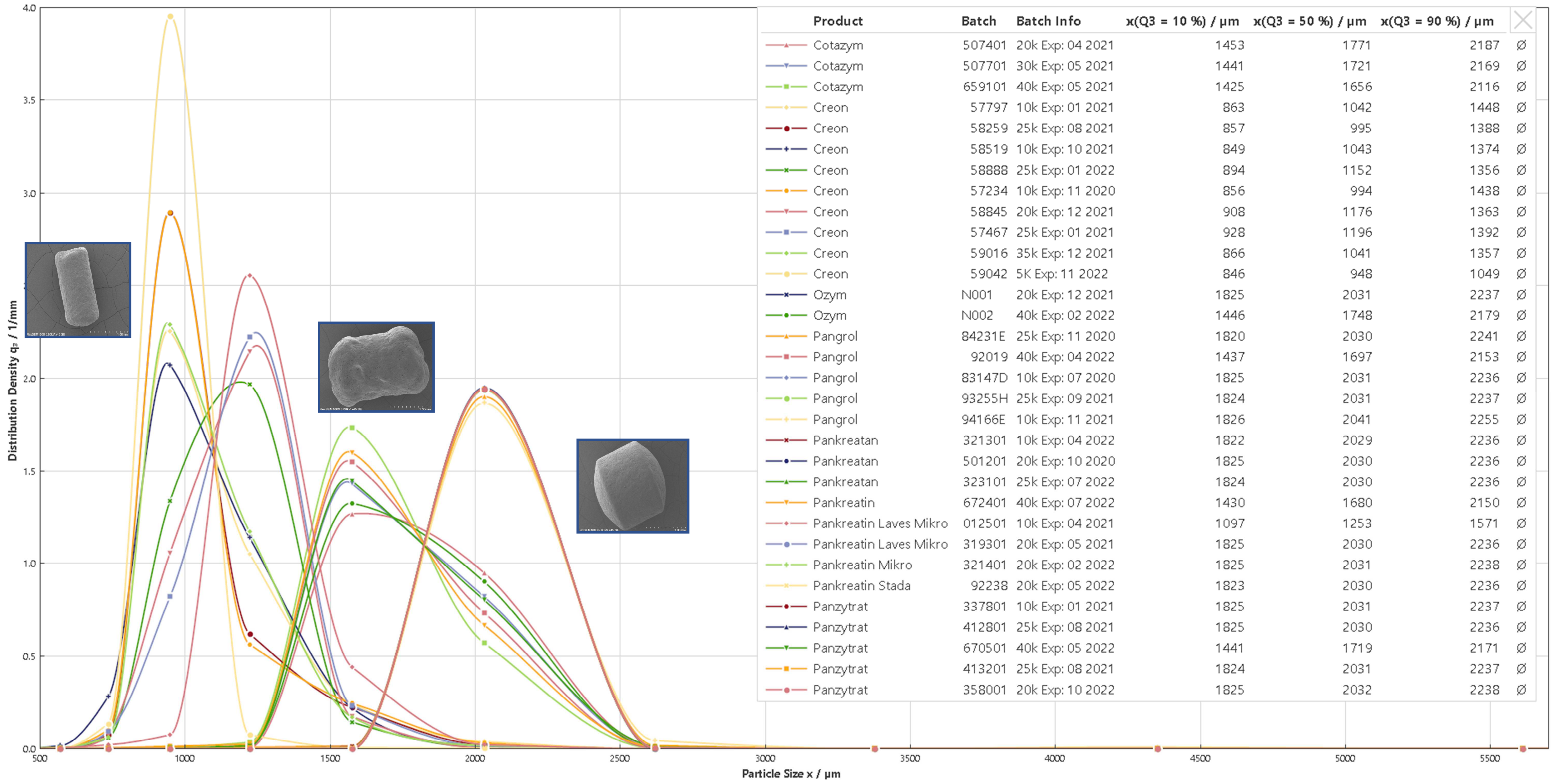

Product	Batch	Batch Info	x(Q3 = 10 %) / µm	x(Q3 = 50 %) / µm	x(Q3 = 90 %) / µm
Cotazym	507401	20k Exp: 04 2021	1453	1771	2187
Cotazym	507701	30k Exp: 05 2021	1441	1721	2169
Cotazym	659101	40k Exp: 05 2021	1425	1656	2116
Creon	57797	10k Exp: 01 2021	863	1042	1448
Creon	58259	25k Exp: 08 2021	857	995	1388
Creon	58519	10k Exp: 10 2021	849	1043	1374
Creon	58888	25k Exp: 01 2022	894	1152	1356
Creon	57234	10k Exp: 11 2020	856	994	1438
Creon	58845	20k Exp: 12 2021	908	1176	1363
Creon	57467	25k Exp: 01 2021	928	1196	1392
Creon	59016	35k Exp: 12 2021	866	1041	1357
Creon	59042	5K Exp: 11 2022	846	948	1049
Ozym	N001	20k Exp: 12 2021	1825	2031	2237
Ozym	N002	40k Exp: 02 2022	1446	1748	2179
Pangrol	84231E	25k Exp: 11 2020	1820	2030	2241
Pangrol	92019	40k Exp: 04 2022	1437	1697	2153
Pangrol	83147D	10k Exp: 07 2020	1825	2031	2236
Pangrol	93255H	25k Exp: 09 2021	1824	2031	2237
Pangrol	94166E	10k Exp: 11 2021	1826	2041	2255
Pankreatan	321301	10k Exp: 04 2022	1822	2029	2236
Pankreatan	501201	20k Exp: 10 2020	1825	2030	2236
Pankreatan	323101	25k Exp: 07 2022	1824	2030	2236
Pankreatin	672401	40k Exp: 07 2022	1430	1680	2150
Pankreatin Laves Mikro	012501	10k Exp: 04 2021	1097	1253	1571
Pankreatin Laves Mikro	319301	20k Exp: 05 2021	1825	2030	2236
Pankreatin Mikro	321401	20k Exp: 02 2022	1825	2031	2238
Pankreatin Stada	92238	20k Exp: 05 2022	1823	2030	2236
Panzytrat	337801	10k Exp: 01 2021	1825	2031	2237
Panzytrat	412801	25k Exp: 08 2021	1825	2030	2236
Panzytrat	670501	40k Exp: 05 2022	1441	1719	2171
Panzytrat	413201	25k Exp: 08 2021	1824	2031	2237
Panzytrat	358001	20k Exp: 10 2022	1825	2032	2238

Figure S8. Representative batches of each presentation - Feret Min

Effective Date: 3/15/2021 5:13:06 AM

Effective ver 1.0

Table S3. Mean (n=3) EQPC

Product	Batch	Strength	D[v,0.1] µm	D[v,0.5] µm	D[v,0.9] µm	Presentation
Cotazym	507401	20k	1788	2093	2680	Pellet (type II)
Cotazym	507701	30k	1779	2081	2646	Pellet (type II)
Cotazym	659101	40k	1659	2040	2548	Pellet (type II)
Creon	57797	10k	961	1260	1700	Pellet (type I)
Creon	58259	25k	945	1230	1636	Pellet (type I)
Creon	58519	10k	925	1235	1686	Pellet (type I)
Creon	58888	25k	1059	1355	1708	Pellet (type I)
Creon	57234	10k	932	1227	1644	Pellet (type I)
Creon	58845	20k	1068	1340	1716	Pellet (type I)
Creon	57467	25k	1071	1350	1737	Pellet (type I)
Creon	59016	35k	976	1259	1676	Pellet (type I)
Creon	59042	5K	912	1177	1384	Pellet (type I)
Ozym	N001	20k	1864	2228	2796	Mini-Tablet
Ozym	N002	40k	1772	2068	2615	Pellet (type II)
Mezym	98013	10k	*N/A	*N/A	*N/A	Tablet
Pangrol	84231E	25k	2330	2607	2884	Mini-Tablet
Pangrol	92019	40k	1761	2078	2639	Pellet (type II)
Pangrol	83147D	10k	2342	2612	2882	Mini-Tablet
Pangrol	93255H	25k	2344	2613	2882	Mini-Tablet
Pangrol	94166E	10k	2346	2622	2898	Mini-Tablet
Pangrol	92027A	20k	**N/A	**N/A	**N/A	Tablet
Pankreatan	321301	10k	1872	2277	2814	Mini-Tablet
Pankreatan	501201	20k	1866	2238	2800	Mini-Tablet
Pankreatan	323101	25k	1858	2204	2784	Mini-Tablet
Pankreatin	672401	40k	1719	2050	2545	Pellet (type II)
Pankreatin Laves Mikro	012501	10k	1255	1579	1985	Pellet (type II)
Pankreatin Laves Mikro	319301	20k	1847	2145	2733	Mini-Tablet
Pankreatin Mikro	321401	20k	1871	2269	2819	Mini-Tablet
Pankreatin Stada	92238	20k	1864	2242	2803	Mini-Tablet
Panzytrat	337801	10k	1864	2230	2802	Mini-Tablet
Panzytrat	412801	25k	1861	2214	2790	Mini-Tablet
Panzytrat	670501	40k	1789	2088	2672	Pellet (type II)
Panzytrat	413201	25k	1860	2213	2795	Mini-Tablet
Panzytrat	358001	20k	1862	2214	2794	Mini-Tablet

*Tablet size = 10.31 mm

**Tablet size = 11.39 mm

Effective Date: 3/15/2021 5:13:06 AM

Effective ver 1.0

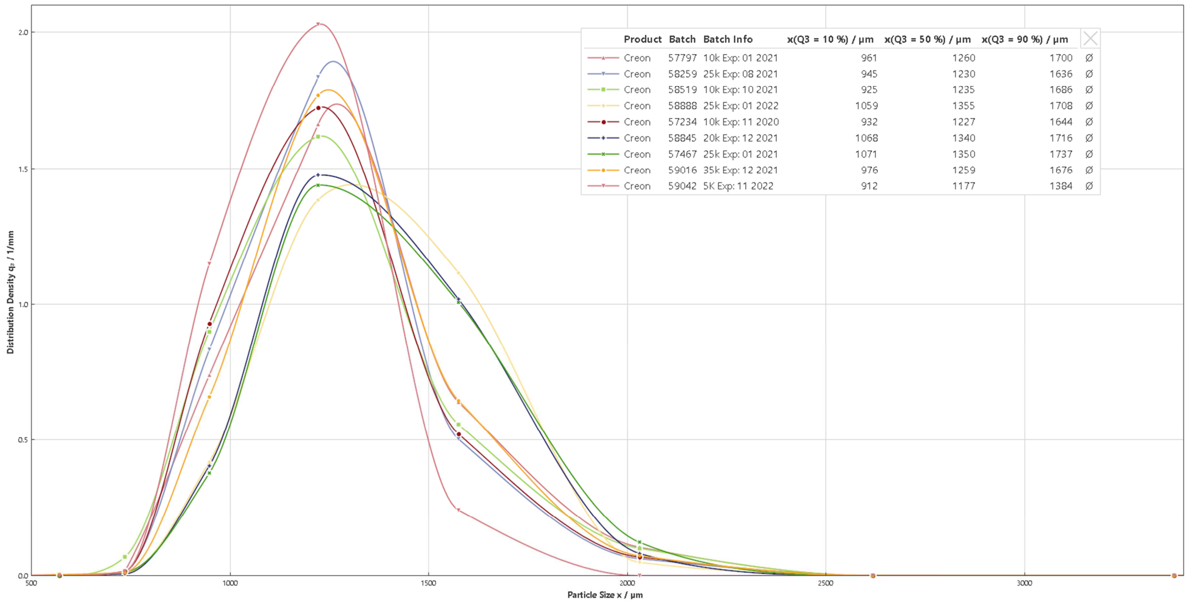

Figure S9. Pellet (type I) – EQPC

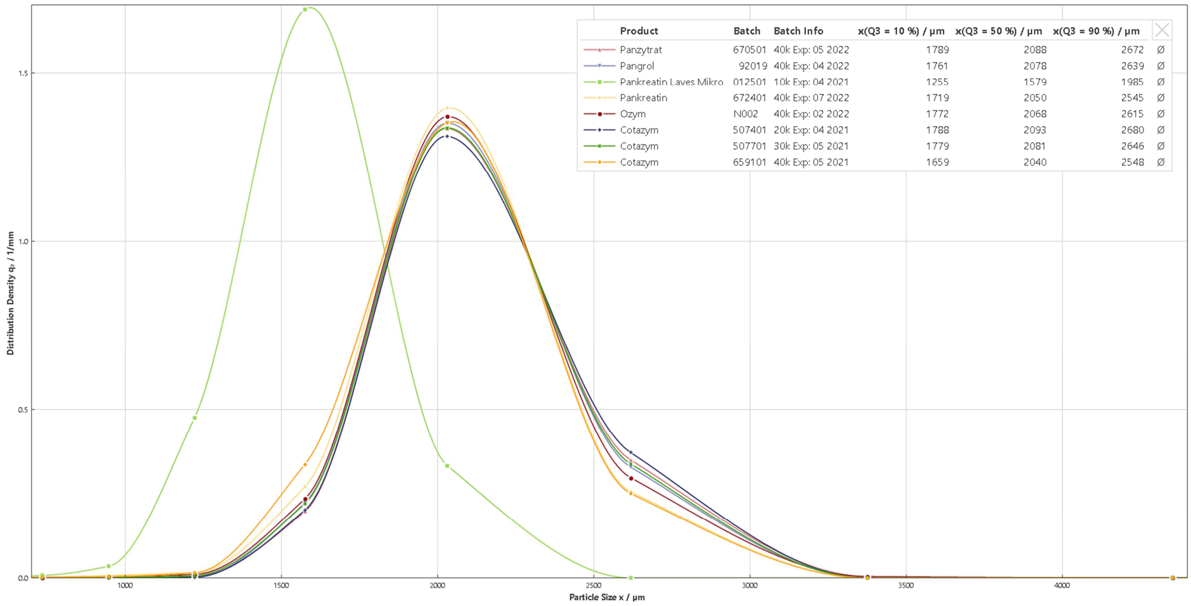

Figure S10. Pellet (type II) – EQPC

Effective Date: 3/15/2021 5:13:06 AM

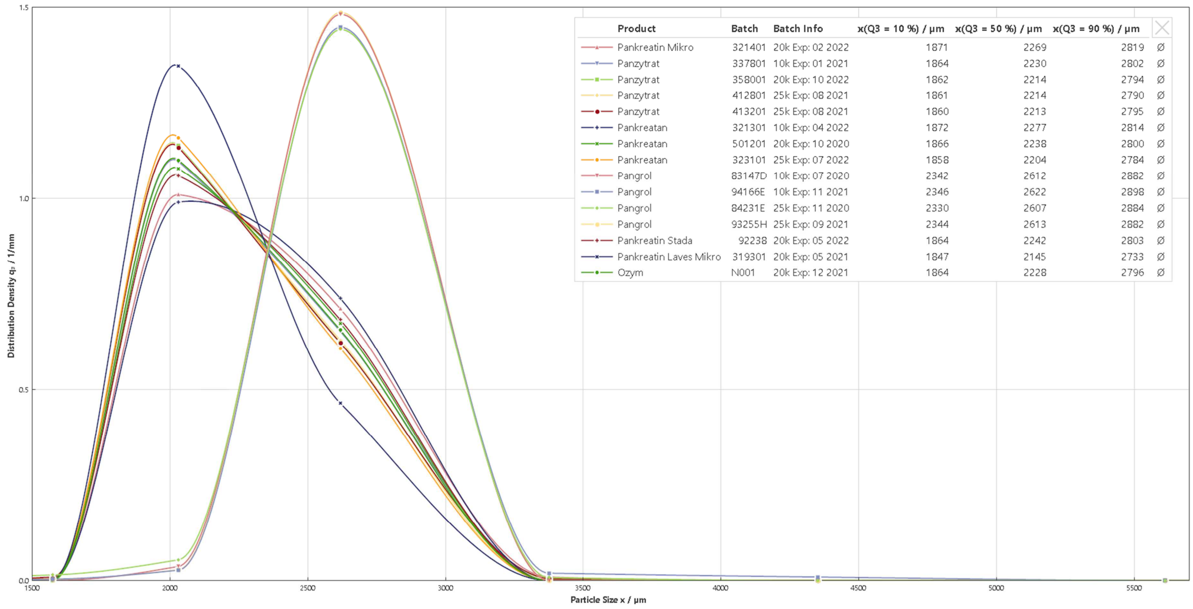

Figure S11. Mini-tablet – EQPC

Effective Date: 3/15/2021 5:13:06 AM

Effective ver 1.0

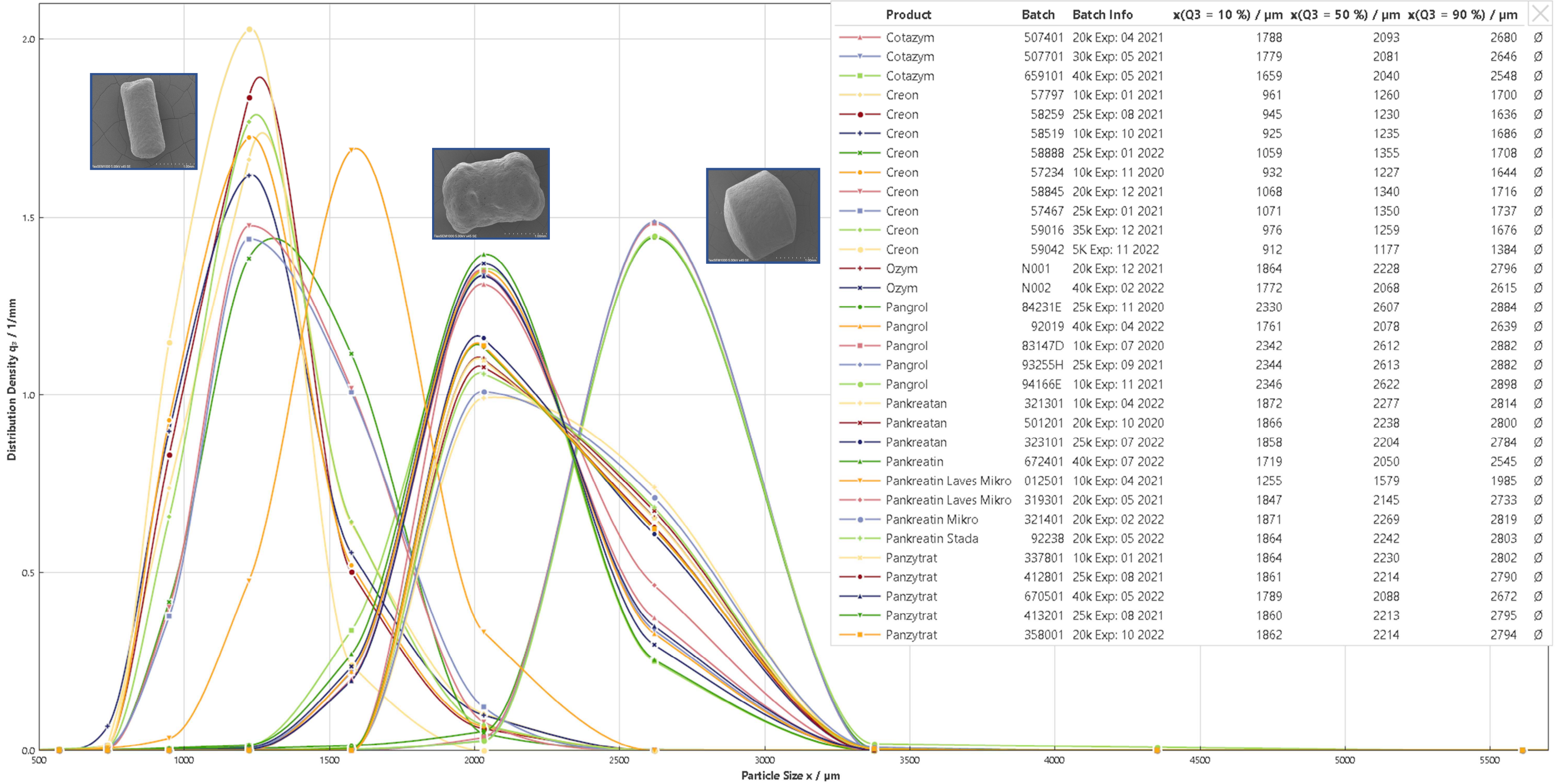

Product	Batch	Batch Info	x(Q3 = 10 %) / µm	x(Q3 = 50 %) / µm	x(Q3 = 90 %) / µm
Cotazym	507401	20k Exp: 04 2021	1788	2093	2680
Cotazym	507701	30k Exp: 05 2021	1779	2081	2646
Cotazym	659101	40k Exp: 05 2021	1659	2040	2548
Creon	57797	10k Exp: 01 2021	961	1260	1700
Creon	58259	25k Exp: 08 2021	945	1230	1636
Creon	58519	10k Exp: 10 2021	925	1235	1686
Creon	58888	25k Exp: 01 2022	1059	1355	1708
Creon	57234	10k Exp: 11 2020	932	1227	1644
Creon	58845	20k Exp: 12 2021	1068	1340	1716
Creon	57467	25k Exp: 01 2021	1071	1350	1737
Creon	59016	35k Exp: 12 2021	976	1259	1676
Creon	59042	5K Exp: 11 2022	912	1177	1384
Ozym	N001	20k Exp: 12 2021	1864	2228	2796
Ozym	N002	40k Exp: 02 2022	1772	2068	2615
Pangrol	84231E	25k Exp: 11 2020	2330	2607	2884
Pangrol	92019	40k Exp: 04 2022	1761	2078	2639
Pangrol	83147D	10k Exp: 07 2020	2342	2612	2882
Pangrol	93255H	25k Exp: 09 2021	2344	2613	2882
Pangrol	94166E	10k Exp: 11 2021	2346	2622	2898
Pankreatan	321301	10k Exp: 04 2022	1872	2277	2814
Pankreatan	501201	20k Exp: 10 2020	1866	2238	2800
Pankreatan	323101	25k Exp: 07 2022	1858	2204	2784
Pankreatin	672401	40k Exp: 07 2022	1719	2050	2545
Pankreatin Laves Mikro	012501	10k Exp: 04 2021	1255	1579	1985
Pankreatin Laves Mikro	319301	20k Exp: 05 2021	1847	2145	2733
Pankreatin Mikro	321401	20k Exp: 02 2022	1871	2269	2819
Pankreatin Stada	92238	20k Exp: 05 2022	1864	2242	2803
Panzytrat	337801	10k Exp: 01 2021	1864	2230	2802
Panzytrat	412801	25k Exp: 08 2021	1861	2214	2790
Panzytrat	670501	40k Exp: 05 2022	1789	2088	2672
Panzytrat	413201	25k Exp: 08 2021	1860	2213	2795
Panzytrat	358001	20k Exp: 10 2022	1862	2214	2794

Figure S12. Representative batches of each presentation - EQPC

Effective Date: 3/15/2021 5:13:06 AM

1.3 PARTICLE COUNTING

The contents of Creon capsules, and those of its competitor products, were imaged using a high resolution Optimax EvoCam. A capsule was emptied onto a clean sheet of paper and gently agitated to disperse the contents (where the product is not contained within a capsule, a representative amount of sample was dispensed onto the paper using the provided spoons/ spatulas). This was repeated in triplicate for each batch.

These images were analyzed using the image analysis function of the ZenCore software (Zeiss Axio Imager light microscope software) to count the capsule contents. Some manual detection of particles was necessary to aid automatic detection where the detection settings were not able to differentiate between particles.

Due to large number of particles present in type II pellet batches, 2 images were taken for each capsule whereby the contents were roughly split in half to improve dispersion of the sample and to minimize touching particles. Images were analyzed as part 1 and part 2 for each replicate.

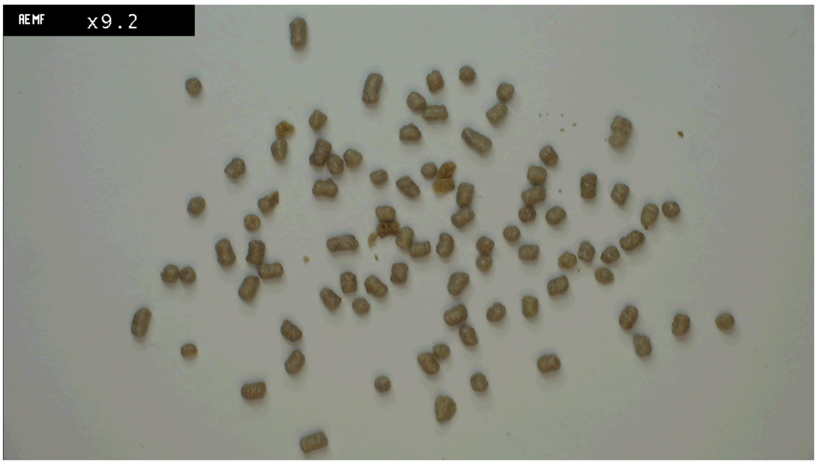

Figure S13. Example of image before analysis (Cotazym 30k 507701 Image 1)

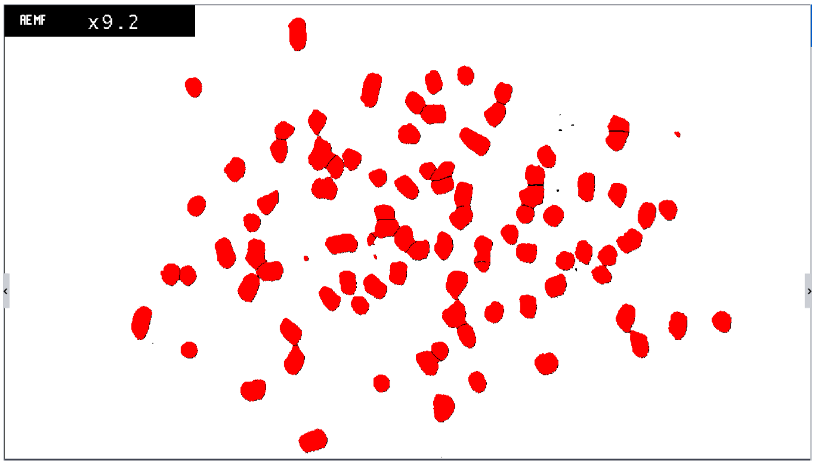

Figure S14. Example of analysed image (Cotazym 30k 507701 Image 1)

Table S4. Particle count results

Product	Batch	Strength	Presentation	Replicate	Total Particle count	Manually Detected Particles
Cotazym	507401	20k	Pellet (type II)	1	62	0
				2	60	0
				3	60	3
Cotazym	507701	30k	Pellet (type II)	1	92	8
				2	93	4
				3	84	6
Cotazym	659101	40k	Pellet (type II)	1	101	4
				2	97	8
				3	145	12
Creon	57797	10k	Pellet (type I)	1	268 (119 + 149)	10 (6 + 4)
				2	257 (104 + 153)	19 (6 + 13)
				3	260 (96 + 164)	17 (2 + 15)
Creon	58259	25k	Pellet (type I)	1	592 (314 + 278)	35 (13 + 22)
				2	608 (337 + 271)	26 (22 + 4)
				3	575 (285 + 290)	23 (17 + 6)
Creon	58519	10k	Pellet (type I)	1	279 (140 + 139)	17 (9 + 8)
				2	292 (140 + 152)	18 (3 + 15)
				3	313 (163 + 150)	20 (7 + 13)
Creon	58888	25k	Pellet (type I)	1	388 (175 + 213)	8 (4 + 4)
				2	412 (239 + 173)	3 (0 + 3)
				3	418 (185 + 233)	8 (3 + 5)
Creon	57234	10k	Pellet (type I)	1	314 (150 + 164)	23 (13 + 10)
				2	301 (142 + 159)	25 (9 + 16)
				3	291 (156 + 135)	32 (17 + 15)
Creon	58845	20k	Pellet (type I)	1	414 (204 + 210)	7 (7 + 0)

Effective Date: 3/15/2021 5:13:06 AM

Effective ver 1.0

				2	387 (193 + 194)	4 (0 + 4)
				3	402 (229 + 173)	3 (1 + 2)
Creon	57467	25k	Pellet (type I)	1	391 (221 + 170)	9 (5 + 4)
				2	384 (215 + 169)	13 (11 + 2)
				3	388 (192 + 196)	11 (8 + 3)
Creon	59016	35k	Pellet (type I)	1	726 (336 + 390)	15 (4 + 11)
				2	765 (296 + 469)	23 (8 + 15)
				3	735 (374 + 361)	36 (15 + 21)
Creon	59042	5k	Pellet (type I)	1	128	2
				2	132	0
				3	124	4
Ozym	N001	20k	Mini-Tablet	1	46	0
				2	43	0
				3	48	0
Ozym	N002	40k	Pellet (type II)	1	94	4
				2	95	3
				3	92	0
Pangrol	84231E	25k	Mini-Tablet	1	48	0
				2	61	0
				3	47	0
Pangrol	92019	40k	Pellet (type II)	1	102	12
				2	96	3
				3	94	9
Pangrol	83147D	10k	Mini-Tablet	1	19	0
				2	20	0
				3	20	0
Pangrol	93255H	25k	Mini-Tablet	1	43	0
				2	45	0
				3	48	0
Pangrol	94166E	10k	Mini-Tablet	1	19	0
				2	20	0
				3	21	0
Pankreatan	321301	10k	Mini-Tablet	1	30	0
				2	22	0
				3	23	0
Pankreatan	501201	20k	Mini-Tablet	1	48	0

Effective Date: 3/15/2021 5:13:06 AM

Effective ver 1.0

				2	45	0
				3	45	0
Pankreatan	323101	25k	Mini-Tablet	1	58	0
				2	60	0
				3	54	0
Pankreatin	672401	40k	Pellet (type II)	1	97	6
				2	108	10
				3	100	11
Pankreatin Laves Mikro	319301	20k	Mini-Tablet	1	43	0
				2	44	0
				3	42	0
Pankreatin Laves Mikro	012501	10k	Pellet (type II)	1	78	6
				2	74	2
				3	81	6
Pankreatin Mikro	321401	20k	Mini-Tablet	1	50	0
				2	49	0
				3	48	0
Pankreatin Stada	92238	20k	Mini-Tablet	1	46	0
				2	49	0
				3	46	0
Panzytrat	337801	10k	Mini-Tablet	1	28	0
				2	26	0
				3	25	0
Panzytrat	412801	25k	Mini-Tablet	1	49	0
				2	51	0
				3	50	0
Panzytrat	670501	40k	Pellet (type II)	1	99	10
				2	96	6
				3	100	8
Panzytrat	413201	25k	Mini-Tablet	1	49	0
				2	48	2
				3	44	0
*Panzytrat	358001	20k	Mini-Tablet	1	50	0

*Only one replicate performed as material is supplied in bottles of 20g and patients use dosing spoons to sample a fixed number of particles (50 or 13).

Effective Date: 3/15/2021 5:13:06 AM

Effective ver 1.0

CONCLUSIONS

SEM

SEM analysis of all Creon and competitor products showed three distinct sample presentations subsequently labelled as type I pellets, type II pellets and Mini-tablets. All Creon batches presented as type I pellets, all Cotazym and other high strength products presented as type II pellets and all other low and mid strength products presented as mini-tablets.

Particle Size

Particle size was evaluated by Feret max, Feret min and EQPC.

Feret max results fall into three populations consistent with the SEM analysis observations, with mini-tablets being the largest (mean D90 = 2886 µm) and type I pellets being the smallest (mean D90 = 2179 µm) of the three presentations.

Feret min and EQPC results fall into four populations. Feret min type I pellets and EQPC type II pellets were divided into two populations, this is likely to be due to the orientation of the pellet as its passes the detector. Particle size of the presentations for feret min and EQPC showed the same trend as feret max results.

Pankreatin Laves Micro batch: 012501 was identified as a type II pellet but showed a smaller particle size and did not sit within the expected population for feret max, feret min or EQPC.

Pangrol 92027A and Mezym 98013 are tablet formulations and consequently were not analysed by QICPIC and instead the particle size was measured using callipers, giving 11.39 mm and 10.31 mm respectively.

Particle Counting

Analysis of images taken for particle counting showed that capsules containing type I pellets had the largest number of individual particles and capsules containing mini-tablets had the least. Particle count range for each of the particle presentations and strengths can be found in Table S5.

Table S5. Particle counting summary

Strength	5k	10k	20k	25k	30k	35k	40k
Type I pellets	124 – 132	257 – 314	387 – 414	384 – 608	-	726 – 765	-
Type II pellets	-	74 – 81	60 – 62	-	84 – 93	-	92 – 145
Mini-tablets	-	19 – 30	42 – 50	43 – 61	-	-	-

Effective Date: 3/15/2021 5:13:06 AM

Effective ver 1.0

APPENDIX

Table S6. Replicate data for Feret Max

Product	Batch	Replicate	Strength	D[v,0.1] µm	D[v,0.5] µm	D[v,0.9] µm
Cotazym	507401	1	20k	1938.48	2582.74	3479.88
Cotazym	507401	2	20k	1943.96	2593.93	3492.38
Cotazym	507401	3	20k	1973.11	2626.72	3602.27
Cotazym	507701	1	30k	1946.91	2591.54	3482.83
Cotazym	507701	2	30k	1926.47	2566.99	3449.75
Cotazym	507701	3	30k	1902.90	2482.12	3333.04
Cotazym	659101	1	40k	1888.00	2457.47	3334.56
Cotazym	659101	2	40k	1873.60	2444.80	3354.96
Cotazym	659101	3	40k	1895.26	2501.78	3390.30
Creon	57797	1	10k	1131.68	1577.71	2245.92
Creon	57797	2	10k	1121.66	1533.57	2159.12
Creon	57797	3	10k	1139.80	1585.27	2268.77
Creon	58259	1	25k	1120.24	1524.74	2173.69
Creon	58259	2	25k	1118.39	1502.11	2112.96
Creon	58259	3	25k	1128.79	1566.65	2196.54
Creon	58519	1	10k	1098.54	1511.97	2191.76
Creon	58519	2	10k	1102.36	1555.69	2226.78
Creon	58519	3	10k	1100.48	1543.27	2239.90
Creon	58888	1	25k	1185.01	1614.22	2173.45
Creon	58888	2	25k	1169.84	1593.87	2153.34
Creon	58888	3	25k	1181.89	1615.12	2184.58
Creon	57234	1	10k	1103.96	1496.47	2145.82
Creon	57234	2	10k	1123.25	1554.72	2221.10
Creon	57234	3	10k	1120.61	1570.64	2205.86
Creon	58845	1	20k	1180.73	1604.49	2197.95
Creon	58845	2	20k	1181.43	1602.85	2221.32
Creon	58845	3	20k	1175.89	1617.02	2245.77
Creon	57467	1	25k	1174.19	1585.73	2241.35
Creon	57467	2	25k	1179.66	1603.12	2295.98
Creon	57467	3	25k	1165.61	1575.28	2201.67
Creon	59016	1	35k	1133.97	1543.67	2169.46

Effective Date: 3/15/2021 5:13:06 AM

Effective ver 1.0

Product	Batch	Replicate	Strength	D[v,0.1] µm	D[v,0.5] µm	D[v,0.9] µm
Creon	59016	2	35k	1126.07	1537.23	2167.18
Creon	59016	3	35k	1133.57	1546.05	2163.79
Creon	59042	1	5K	1102.89	1468.21	2042.28
Creon	59042	2	5K	1093.14	1434.94	1991.63
Creon	59042	3	5K	1095.01	1442.99	2007.77
Lipancrea	011119	1	8k	1555.56	2142.07	2795.42
Lipancrea	011119	2	8k	1545.60	2131.81	2848.60
Lipancrea	011119	3	8k	1525.05	2086.80	2799.32
Ozym	N001	1	20k	2348.25	2621.77	2895.29
Ozym	N001	2	20k	2343.82	2612.87	2881.92
Ozym	N001	3	20k	2343.07	2612.45	2881.83
Ozym	N002	1	40k	1928.67	2555.09	3487.10
Ozym	N002	2	40k	1920.32	2550.49	3446.26
Ozym	N002	3	40k	1901.77	2478.67	3273.11
Pangrol	84231E	1	25k	2333.11	2609.17	2885.22
Pangrol	84231E	2	25k	2333.47	2607.12	2880.77
Pangrol	84231E	3	25k	2347.91	2628.53	2909.14
Pangrol	92019	1	40k	1924.14	2543.22	3430.75
Pangrol	92019	2	40k	1905.57	2529.84	3469.65
Pangrol	92019	3	40k	1922.52	2513.40	3314.77
Pangrol	83147D	1	10k	2351.22	2616.98	2882.74
Pangrol	83147D	2	10k	2348.38	2615.40	2882.42
Pangrol	83147D	3	10k	2349.02	2615.76	2882.50
Pangrol	93255H	1	25k	2353.47	2618.23	2882.99
Pangrol	93255H	2	25k	2351.98	2617.40	2882.82
Pangrol	93255H	3	25k	2349.93	2616.26	2882.60
Pangrol	94166E	1	10k	2357.39	2646.11	2934.83
Pangrol	94166E	2	10k	2350.69	2625.88	2901.08
Pangrol	94166E	3	10k	2354.73	2624.69	2894.65
Pankreatan	321301	1	10k	2344.23	2613.10	2881.96
Pankreatan	321301	2	10k	2344.79	2613.41	2882.03
Pankreatan	321301	3	10k	2340.08	2610.79	2881.50
Pankreatan	501201	1	20k	2342.08	2611.90	2881.72
Pankreatan	501201	2	20k	2346.50	2614.36	2882.22
Pankreatan	501201	3	20k	2346.81	2614.53	2882.25
Pankreatan	323101	1	25k	2341.48	2611.57	2881.66
Pankreatan	323101	2	25k	2342.26	2612.00	2881.74

Effective Date: 3/15/2021 5:13:06 AM

Effective ver 1.0

Product	Batch	Replicate	Strength	D[v,0.1] µm	D[v,0.5] µm	D[v,0.9] µm
Pankreatan	323101	3	25k	2345.23	2613.65	2882.07
Pankreatin	672401	1	40k	1914.57	2511.86	3351.17
Pankreatin	672401	2	40k	1914.29	2548.95	3450.36
Pankreatin	672401	3	40k	1897.94	2496.49	3443.96
Pankreatin Laves Mikro	012501	1	10k	1460.04	1964.66	2667.34
Pankreatin Laves Mikro	012501	2	10k	1476.47	2004.41	2680.66
Pankreatin Laves Mikro	012501	3	10k	1465.24	1968.65	2654.19
Pankreatin Laves Mikro	319301	1	20k	2341.00	2611.30	2881.60
Pankreatin Laves Mikro	319301	2	20k	2342.56	2612.17	2881.78
Pankreatin Laves Mikro	319301	3	20k	2337.48	2609.35	2881.21
Pankreatin Mikro	321401	1	20k	2348.44	2622.60	2896.76
Pankreatin Mikro	321401	2	20k	2335.92	2608.48	2881.04
Pankreatin Mikro	321401	3	20k	2344.82	2622.13	2899.43
Pankreatin Stada	92238	1	20k	2338.89	2610.13	2881.37
Pankreatin Stada	92238	2	20k	2344.23	2613.10	2881.96
Pankreatin Stada	92238	3	20k	2342.91	2612.36	2881.82
Panzytrat	337801	1	10k	2347.14	2614.71	2882.29
Panzytrat	337801	2	10k	2337.47	2609.34	2881.21
Panzytrat	337801	3	10k	2343.10	2619.83	2896.56
Panzytrat	412801	1	25k	2343.21	2612.53	2881.85
Panzytrat	412801	2	25k	2347.79	2615.07	2882.36
Panzytrat	412801	3	25k	2344.58	2613.29	2882.00
Panzytrat	670501	1	40k	1938.21	2592.85	3588.91
Panzytrat	670501	2	40k	1937.58	2573.55	3425.20
Panzytrat	670501	3	40k	1944.12	2571.90	3412.86
Panzytrat	413201	1	25k	2349.33	2615.93	2882.53
Panzytrat	413201	3	25k	2337.46	2609.33	2881.21
Panzytrat	413201	2	25k	2336.14	2615.57	2895.01
Panzytrat	358001	1	20k	2342.07	2611.90	2881.72
Panzytrat	358001	2	20k	2347.32	2620.84	2894.36
Panzytrat	358001	3	20k	2340.82	2611.20	2881.58

Table S7. Replicate data for Feret Min

Product	Batch	Replicate	Strength	D[v,0.1] µm	D[v,0.5] µm	D[v,0.9] µm
Creon	57797	1	10k	861.98	1035.72	1486.67
Creon	57797	2	10k	860.34	1029.74	1365.63
Creon	57797	3	10k	867.88	1059.07	1491.40

Effective Date: 3/15/2021 5:13:06 AM

Effective ver 1.0

Product	Batch	Replicate	Strength	D[v,0.1] µm	D[v,0.5] µm	D[v,0.9] µm
Creon	58259	1	25k	857.12	996.47	1385.44
Creon	58259	2	25k	855.63	989.74	1348.37
Creon	58259	3	25k	857.95	999.11	1430.75
Panzytrat	337801	1	10k	1824.81	2030.36	2235.91
Panzytrat	337801	2	10k	1825.00	2030.47	2235.94
Panzytrat	337801	3	10k	1825.63	2032.38	2239.14
Panzytrat	412801	1	25k	1824.44	2030.16	2235.87
Panzytrat	412801	2	25k	1824.49	2030.19	2235.88
Panzytrat	412801	3	25k	1824.78	2030.35	2235.91
Panzytrat	670501	1	40k	1438.25	1699.44	2164.04
Panzytrat	670501	2	40k	1444.10	1736.93	2183.64
Panzytrat	670501	3	40k	1441.53	1719.23	2165.41
Pangrol	84231E	1	25k	1815.96	2028.28	2240.60
Pangrol	84231E	2	25k	1817.96	2029.57	2241.18
Pangrol	84231E	3	25k	1825.24	2033.01	2240.78
Pangrol	92019	1	40k	1443.18	1721.27	2173.93
Pangrol	92019	2	40k	1435.20	1677.85	2137.01
Pangrol	92019	3	40k	1433.27	1690.55	2148.68
Pangrol	83147D	1	10k	1825.20	2030.58	2235.96
Pangrol	83147D	2	10k	1825.29	2030.63	2235.97
Pangrol	83147D	3	10k	1824.78	2030.35	2235.91
Pankreatin Mikro	321401	1	20k	1824.92	2031.97	2239.02
Pankreatin Mikro	321401	2	20k	1824.49	2030.18	2235.88
Pankreatin Mikro	321401	3	20k	1824.77	2031.77	2238.76
Cotazym	507401	1	20k	1445.66	1738.72	2180.56
Cotazym	507401	2	20k	1451.10	1759.13	2187.04
Cotazym	507401	3	20k	1461.85	1815.65	2192.97
Ozym	N001	1	20k	1824.51	2031.39	2238.26
Ozym	N001	2	20k	1824.52	2030.20	2235.88
Ozym	N001	3	20k	1824.65	2030.27	2235.90
Lipancrea	011119	1	8k	1114.32	1320.16	1709.64
Lipancrea	011119	2	8k	1111.45	1313.92	1707.51
Lipancrea	011119	3	8k	1113.20	1317.21	1692.43
Creon	58519	1	10k	848.30	1047.31	1369.46
Creon	58519	2	10k	849.11	1032.20	1374.81
Creon	58519	3	10k	850.30	1048.52	1377.53
Creon	58888	1	25k	897.83	1158.57	1356.40

Effective Date: 3/15/2021 5:13:06 AM

Effective ver 1.0

Product	Batch	Replicate	Strength	D[v,0.1] µm	D[v,0.5] µm	D[v,0.9] µm
Creon	58888	2	25k	888.28	1142.88	1354.42
Creon	58888	3	25k	894.52	1155.04	1357.05
Panzytrat	413201	1	25k	1825.29	2030.63	2235.97
Panzytrat	413201	3	25k	1822.74	2029.21	2235.68
Pankreatin Stada	92238	1	20k	1821.14	2028.32	2235.51
Pankreatin Stada	92238	2	20k	1824.39	2030.13	2235.87
Pankreatin Stada	92238	3	20k	1824.30	2030.08	2235.86
Pankreatan	321301	1	10k	1821.23	2028.37	2235.52
Pankreatan	321301	2	10k	1823.75	2029.77	2235.80
Pankreatan	321301	3	10k	1822.47	2029.06	2235.65
Pankreatan	501201	1	20k	1824.22	2030.03	2235.85
Pankreatan	501201	2	20k	1825.02	2030.48	2235.94
Pankreatan	501201	3	20k	1825.29	2030.63	2235.97
Pankreatan	323101	1	25k	1823.47	2029.62	2235.77
Pankreatan	323101	2	25k	1823.46	2029.61	2235.76
Pankreatan	323101	3	25k	1825.08	2030.51	2235.94
Pankreatin Laves Mikro	012501	1	10k	1096.71	1250.54	1552.63
Pankreatin Laves Mikro	012501	2	10k	1097.69	1259.48	1597.66
Pankreatin Laves Mikro	012501	3	10k	1095.94	1249.99	1561.61
Pankreatin Laves Mikro	319301	1	20k	1823.77	2029.79	2235.80
Pankreatin Laves Mikro	319301	2	20k	1825.29	2030.63	2235.97
Pankreatin Laves Mikro	319301	3	20k	1824.84	2030.38	2235.92
Pankreatin	672401	1	40k	1431.17	1676.76	2146.68
Pankreatin	672401	2	40k	1428.04	1680.03	2150.02
Pankreatin	672401	3	40k	1431.66	1684.62	2152.17
Ozym	N002	1	40k	1444.70	1742.58	2175.18
Ozym	N002	2	40k	1443.19	1747.28	2176.88
Ozym	N002	3	40k	1449.07	1753.02	2185.12
Cotazym	507701	1	30k	1439.60	1725.70	2168.62
Cotazym	507701	2	30k	1442.17	1727.98	2169.47
Cotazym	507701	3	30k	1440.89	1708.92	2167.47
Cotazym	659101	1	40k	1427.14	1658.88	2116.96
Cotazym	659101	2	40k	1421.81	1651.16	2107.71
Cotazym	659101	3	40k	1426.07	1657.03	2123.62
Panzytrat	413201	2	25k	1825.32	2032.31	2239.30
Creon	57234	1	10k	851.88	979.34	1316.03
Creon	57234	2	10k	857.62	999.05	1440.88

Effective Date: 3/15/2021 5:13:06 AM

Effective ver 1.0

Product	Batch	Replicate	Strength	D[v,0.1] µm	D[v,0.5] µm	D[v,0.9] µm
Creon	57234	3	10k	857.54	1004.92	1556.47
Creon	58845	1	20k	909.81	1176.49	1360.56
Creon	58845	2	20k	908.68	1176.30	1363.63
Creon	58845	3	20k	906.88	1174.06	1363.32
Creon	57467	1	25k	932.82	1197.04	1374.68
Creon	57467	2	25k	933.24	1201.32	1431.66
Creon	57467	3	25k	918.93	1189.14	1371.04
Creon	59016	1	35k	867.11	1047.99	1359.15
Creon	59016	2	35k	862.21	1027.62	1352.79
Creon	59016	3	35k	867.89	1046.97	1357.79
Pangrol	93255H	1	25k	1825.29	2030.63	2235.97
Pangrol	93255H	2	25k	1823.68	2029.73	2235.79
Pangrol	93255H	3	25k	1824.19	2031.35	2238.51
Pangrol	94166E	1	10k	1827.33	2046.22	2265.11
Pangrol	94166E	2	10k	1824.31	2036.67	2249.04
Pangrol	94166E	3	10k	1826.97	2039.13	2251.30
Panzytrat	358001	1	20k	1825.29	2030.63	2235.97
Panzytrat	358001	2	20k	1825.59	2032.09	2238.59
Panzytrat	358001	3	20k	1825.54	2031.87	2238.19
Creon	59042	1	5K	846.44	948.28	1050.12
Creon	59042	2	5K	846.10	946.50	1046.91
Creon	59042	3	5K	846.53	947.79	1049.05

Table S8. Replicate data for EQPC

Product	Batch	Replicate	Strength	D[v,0.1] µm	D[v,0.5] µm	D[v,0.9] µm
Cotazym	507401	1	20k	1774.54	2082.52	2666.37
Cotazym	507401	2	20k	1791.96	2095.71	2681.96
Cotazym	507401	3	20k	1798.66	2102.17	2690.66
Cotazym	507701	1	30k	1787.86	2095.31	2684.65
Cotazym	507701	2	30k	1783.66	2090.23	2676.65
Cotazym	507701	3	30k	1766.56	2057.66	2576.77
Cotazym	659101	1	40k	1655.52	2035.54	2518.71
Cotazym	659101	2	40k	1605.20	2032.01	2560.25
Cotazym	659101	3	40k	1717.75	2051.20	2565.55
Creon	57797	1	10k	956.20	1259.80	1701.76
Creon	57797	2	10k	946.79	1244.98	1671.44
Creon	57797	3	10k	979.27	1275.35	1725.99

Effective Date: 3/15/2021 5:13:06 AM

Effective ver 1.0

Product	Batch	Replicate	Strength	D[v,0.1] µm	D[v,0.5] µm	D[v,0.9] µm
Creon	58259	1	25k	942.25	1226.25	1652.63
Creon	58259	2	25k	943.28	1221.50	1601.31
Creon	58259	3	25k	948.65	1242.06	1655.40
Creon	58519	1	10k	924.10	1232.91	1677.12
Creon	58519	2	10k	928.04	1234.98	1675.20
Creon	58519	3	10k	922.13	1238.03	1706.46
Creon	58888	1	25k	1071.32	1366.61	1707.25
Creon	58888	2	25k	1038.04	1346.82	1703.92
Creon	58888	3	25k	1068.54	1350.73	1713.46
Creon	57234	1	10k	923.21	1207.19	1583.43
Creon	57234	2	10k	937.82	1232.17	1656.09
Creon	57234	3	10k	935.22	1241.39	1693.37
Creon	58845	1	20k	1069.97	1347.55	1713.67
Creon	58845	2	20k	1068.04	1329.75	1710.29
Creon	58845	3	20k	1067.38	1342.22	1725.23
Creon	57467	1	25k	1072.40	1346.81	1736.97
Creon	57467	2	25k	1077.80	1360.03	1747.07
Creon	57467	3	25k	1064.21	1343.81	1725.93
Creon	59016	1	35k	981.72	1262.39	1680.50
Creon	59016	2	35k	958.12	1247.52	1665.56
Creon	59016	3	35k	989.12	1267.49	1681.69
Creon	59042	1	5K	911.80	1178.83	1410.48
Creon	59042	2	5K	912.41	1174.85	1368.47
Creon	59042	3	5K	911.57	1175.98	1372.30
Lipancrea	011119	1	8k	1378.55	1652.34	2128.03
Lipancrea	011119	2	8k	1379.62	1647.37	2127.54
Lipancrea	011119	3	8k	1361.87	1640.00	2115.91
Ozym	N001	1	20k	1865.02	2233.17	2799.16
Ozym	N001	2	20k	1867.95	2244.15	2803.43
Ozym	N001	3	20k	1858.88	2206.49	2786.56
Ozym	N002	1	40k	1761.75	2070.93	2635.17
Ozym	N002	2	40k	1774.84	2080.25	2659.92
Ozym	N002	3	40k	1780.40	2054.17	2550.84
Pangrol	84231E	1	25k	2321.65	2600.55	2879.45
Pangrol	84231E	2	25k	2325.05	2602.44	2879.83
Pangrol	84231E	3	25k	2341.88	2617.76	2893.64
Pangrol	92019	1	40k	1794.19	2090.46	2666.61

Effective Date: 3/15/2021 5:13:06 AM

Effective ver 1.0

Product	Batch	Replicate	Strength	D[v,0.1] µm	D[v,0.5] µm	D[v,0.9] µm
Pangrol	92019	2	40k	1694.24	2076.99	2672.98
Pangrol	92019	3	40k	1793.48	2067.15	2577.82
Pangrol	83147D	1	10k	2347.00	2614.63	2882.27
Pangrol	83147D	2	10k	2334.06	2607.44	2880.83
Pangrol	83147D	3	10k	2343.94	2612.93	2881.93
Pangrol	93255H	1	25k	2346.56	2614.39	2882.22
Pangrol	93255H	2	25k	2342.14	2611.93	2881.73
Pangrol	93255H	3	25k	2342.53	2612.16	2881.78
Pangrol	94166E	1	10k	2353.20	2632.20	2911.21
Pangrol	94166E	2	10k	2340.34	2617.10	2893.86
Pangrol	94166E	3	10k	2343.53	2616.75	2889.97
Pankreatan	321301	1	10k	1874.02	2290.60	2817.46
Pankreatan	321301	2	10k	1870.95	2267.90	2811.42
Pankreatan	321301	3	10k	1872.29	2271.57	2812.50
Pankreatan	501201	1	20k	1869.85	2260.58	2809.14
Pankreatan	501201	2	20k	1864.55	2230.42	2798.00
Pankreatan	501201	3	20k	1863.56	2222.05	2794.24
Pankreatan	323101	1	25k	1853.12	2181.88	2771.13
Pankreatan	323101	2	25k	1859.65	2214.62	2790.87
Pankreatan	323101	3	25k	1861.79	2215.12	2790.94
Pankreatin	672401	1	40k	1756.36	2046.76	2511.43
Pankreatin	672401	2	40k	1706.55	2067.11	2638.60
Pankreatin	672401	3	40k	1694.89	2037.54	2485.52
Pankreatin Laves Mikro	012501	1	10k	1247.24	1573.18	1966.33
Pankreatin Laves Mikro	012501	2	10k	1267.31	1588.06	2018.81
Pankreatin Laves Mikro	012501	3	10k	1251.74	1575.55	1969.36
Pankreatin Laves Mikro	319301	1	20k	1844.17	2130.98	2714.45
Pankreatin Laves Mikro	319301	2	20k	1850.86	2158.49	2750.13
Pankreatin Laves Mikro	319301	3	20k	1846.93	2144.09	2733.74
Pankreatin Mikro	321401	1	20k	1876.58	2301.05	2832.34
Pankreatin Mikro	321401	2	20k	1864.10	2235.43	2800.15
Pankreatin Mikro	321401	3	20k	1870.98	2269.81	2825.48
Pankreatin Stada	92238	1	20k	1862.05	2255.45	2807.64
Pankreatin Stada	92238	2	20k	1866.25	2238.61	2801.34
Pankreatin Stada	92238	3	20k	1864.71	2232.63	2798.94
Panzytrat	337801	1	10k	1866.24	2241.58	2802.51
Panzytrat	337801	2	10k	1865.88	2237.23	2800.80

Effective Date: 3/15/2021 5:13:06 AM

Effective ver 1.0

Product	Batch	Replicate	Strength	D[v,0.1] µm	D[v,0.5] µm	D[v,0.9] µm
Panzytrat	337801	3	10k	1861.15	2210.12	2804.03
Panzytrat	412801	1	25k	1860.99	2209.19	2787.85
Panzytrat	412801	2	25k	1860.85	2208.49	2787.48
Panzytrat	412801	3	25k	1862.62	2224.03	2795.24
Panzytrat	670501	1	40k	1774.31	2075.58	2664.50
Panzytrat	670501	2	40k	1796.70	2095.69	2677.53
Panzytrat	670501	3	40k	1794.92	2094.02	2675.13
Panzytrat	413201	1	25k	1861.01	2209.26	2787.89
Panzytrat	413201	3	25k	1857.61	2214.32	2790.89
Panzytrat	413201	2	25k	1861.58	2215.70	2806.29
Panzytrat	358001	1	20k	1859.95	2203.91	2784.95
Panzytrat	358001	2	20k	1864.10	2224.68	2808.46
Panzytrat	358001	3	20k	1861.64	2212.39	2789.52

Effective Date: 3/15/2021 5:13:06 AM

Effective ver 1.0

Part 2 - Enzyme Activity and Enzyme Release Kinetics of Kreon and its German Competitor Products

Disclaimer: Confidential, personal or proprietary information and proprietary images have been redacted from the original report.

Effective Date: 3/31/2021 4:45:06 AM

**Part 2 - Enzyme Activity and Enzyme Release Kinetics**

Table of Contents

1. Aim & Introduction 4
2. Activity & Sample Categories 5
3. Methodology 5
4. Procedure 6
5. Acceptance Criteria & Aberrant Data Investigation 7
6. Results 11
7. Version history 52
Appendix 1 – Sample Details 53

Effective Date: 3/31/2021 4:45:06 AM

Effective ver 1.0

**Part 2 - Enzyme Activity and Enzyme Release Kinetics**

List of Tables

Table S1: Methods used for analysis 5
Table S2: Replicates and determinations performed for each method 6
Table S3: Lipase Activity: Analysis Repeated 8
Table S4: Amylase Activity: Analysis Repeated 8
Table S5: Protease Activity: Analysis Repeated 10
Table S6: Enzyme Release Kinetics: Analysis Repeated 10
Table S7: Lipase Activity Reported Results 11
Table S8: Amylase Activity Reported Results 16
Table S9: Total Protease Activity Reported Results 21
Table S10: Enzyme Release Kinetics Reported Results (Residual Lipase Activity / Ph.Eur.-units) 26
Table S11: Enzyme Release Kinetics Reported Results (Residual Lipase Activity as % of Lipase Activity) 36

List of Figures

Figure S1: Lipase Activity as % of Label Claim 15
Figure S2: Amylase Activity as % of Label Claim 20
Figure S3: Protease Activity as % Label Claim 25
Figure S4: Residual Lipase Activity (as % Lipase Activity) vs Time-Point - pH Test = 1 to 6 46
Figure S5: Residual Lipase Activity (as % Lipase Activity) vs Time-Point - pH Test = 4 to 6 47
Figure S6: Residual Lipase Activity (as % Lipase Activity) vs Time-Point - pH Test = 5 to 6 48
Figure S7: Residual Lipase Activity (as % of Lipase Activity) vs Time (mins) - pH Test = 1 to 6 Grouped by Individual Strength 49
Figure S8: Residual Lipase Activity (as % of Lipase Activity) vs Time (mins) - pH Test = 4 to 6 Grouped by Individual Strength 50
Figure S9: Residual Lipase Activity (as % of Lipase Activity) vs Time (mins) - pH Test = 5 to 6 Grouped by Individual Strength 51

Effective Date: 3/31/2021 4:45:06 AM

Effective ver 1.0

**Part 2 - Enzyme Activity and Enzyme Release Kinetics**

1. AIM & INTRODUCTION

Creon® is a pancreatic enzyme replacement therapy prescribed in response to endocrine pancreatic insufficiency (EPI). This condition is typically a result of cystic fibrosis, pancreatitis, a pancreatectomy or other pancreatic function impacting conditions. The therapy replaces protease, amylase and lipase, enzymes that would otherwise be produced by a healthy pancreas.

The active ingredient in the formulation, pancreatin, is derived from healthy porcine pancreases. In the Creon product pancreatin is formulated into micro pellets, cylindrical in shape, approximately 1mm in diameter and 2mm in length. These pellets are enteric-coated so that the enzymes are released in the duodenum. The final stage of the pellet formulation is to encapsulate them in a gelatine capsule.

In this report, Enzyme (Lipase, Amylase and total Protease) activity and the kinetics of enzyme release of Creon and a number of competitor products were determined to compare their performance in support of ongoing marketing initiatives.

Refer to Part 1: “Physical Characterisation” for size and shape analysis of these products.

Effective Date: 3/31/2021 4:45:06 AM

Effective ver 1.0

**Part 2 - Enzyme Activity and Enzyme Release Kinetics**

2. ACTIVITY & SAMPLE CATEGORIES

All samples are of GMP origin and were analysed under an Exploratory Development protocol. Refer to Appendix 1 for sample details.

3. METHODOLOGY

Table S1: Methods used for analysis

Test	Method No.	Title
Lipase activity	MGR-METH-859065*	Determination of the lipase activity of pancreas power containing preparations
Amylase activity	MGR-METH-859066 v1.0	Determination of the amylase activity of pancreas power containing preparations
Total Protease activity	MGR-METH-859075 v1.0	Determination of the total proteolytic activity of pancreas power containing preparations
Enzyme release kinetics	MGR-METH-859076 v1.0	Determination of enzyme release kinetics of pancreas power containing preparations

* = v1.0 used for Days 1 to 8 and v2.0 for Day 9 (Mezym F 10K B/N: 98013 and Pangrol 20K B/N: 92027A only). MGR-METH-859065 was updated to include the following changes:

- Section 3.0: Use of bottle top dispensers changed to Autopipettes.
- Section 6.1: Increased standard and sample solution stability from "analyse immediately" to "analyse within 4 hours of preparation".
- Section 7.1: Added requirement to calibrate the pH probe on the autotitrator to reflect the current practice of calibrating the pH probe daily prior to use.
- Section 7.2: Updated to allow for test sample volume changes to align with Ph.Eur method for Pancreas Powder.
- Section 9.0: Updated to include applicable corrections in lipase activity calculations where test sample volume changes are required.
- Section 11.0: Updated with informal validation reference.

There is no impact on the data for Days 1 to 8 when compared to data for Day 9

Effective Date: 3/31/2021 4:45:06 AM

Effective ver 1.0

**Part 2 - Enzyme Activity and Enzyme Release Kinetics**

4. PROCEDURE

The tests listed in Table S1 were performed in the MGRG ACD laboratory (Sandwich, UK) to generate replicate determinations as listed in Table S2 for each sample listed in Appendix 1.

Test	No. of Capsules or Tablets per replicate*	No. of Replicates	Target Enzyme Activity in Weighing	No. of Determinations
Lipase Activity	20	1	2500	2 titrations/replicate
Amylase Activity	20	1	3000	2 titrations/replicate
Total Protease Activity	20	1	260	3 determinations/replicate
Enzyme Release Kinetics	**	1/condition	15000 (Lipase)	1 titration/timepoint

* = For products in pellet form, a minimum of 5g of pellets were weighed.

** = An appropriate number of capsules were used to prepare a bulk sample with 80-100K Lipase units on day of analysis.

Effective Date: 3/31/2021 4:45:06 AM

Effective ver 1.0

**Part 2 - Enzyme Activity and Enzyme Release Kinetics**

5. ACCEPTANCE CRITERIA & ABERRANT DATA INVESTIGATION

Acceptance Criteria - Method Performance
Acceptance criteria for method performance were listed within each method and was met for all methods except for the enzyme release kinetics method MGR-METH-859076 v1.0 Section 7: criteria to maintain the temperature of the media in the vessels at 37°C ± 1°C.

The USP/Ph Eur requirements for temperature control in Disintegration Apparatus is 37 ± 2°C and is the tolerance defined for the equipment by the equipment manufacturer. During the analyses, the disintegration tester is set to 37.0°C. The temperature readings taken before each sampling timepoint demonstrate that the temperature of the media in the vessels was maintained at the pharmacopeial requirements of 37°C ± 2°C, rather than 37°C ± 1°C. The method criteria have been set tighter than the equipment/pharmacopeial requirements. The Disintegration Apparatus temperature settings were maintained throughout the analyses for this protocol, therefore the application of the 37°C ± 2°C criteria is not considered to have a significant impact on the data and the data are reported.

Acceptance Criteria - Results
Acceptance criteria for all analyses performed on products is report results.

Aberrant Data Investigation
Aberrant data were investigated as per Laboratory Investigations SOP-000551991 for an exploratory development activity on a sample of GMP origin.

Effective Date: 3/31/2021 4:45:06 AM

Effective ver 1.0

**Part 2 - Enzyme Activity and Enzyme Release Kinetics**

Table S3: Lipase Activity: Analysis Repeated

Supplier	Product	Strength (Lipase)	Batch
Abbott	Kreon	5K	59042
Allergan	Panzytrat	10K	337801
Allergan	Panzytrat	20K	358001

[redacted]

Table S4: Amylase Activity: Analysis Repeated

Supplier	Product	Strength (Lipase)	Batch
Abbott	Kreon	10K	58519
Abbott	Kreon	20K	58845
Abbott	Kreon	35K	59016
Berlin-Chemie AG	Pangrol	40K	92019
Allergan	Panzytrat	40K	670501
Abbott	Kreon	5K	59042

Effective Date: 3/31/2021 4:45:06 AM

Effective ver 1.0

Part 2- Enzyme Activity and Enzyme Release Kinetics

Supplier	Product	Strength (Lipase)	Batch
Nordmark	Pankreatin	40K	672401

[redacted]

Effective Date: 3/31/2021 4:45:06 AM

Effective ver 1.0

Part 2 - Enzyme Activity and Enzyme Release Kinetics

Table S5: Protease Activity: Analysis Repeated

Supplier	Product	Strength (Lipase)	Batch
Abbott	Kreon	25K	58888
Trommsdorff	Ozym	40K	N002
Cheplapharm	Cotazym	20K	507401

[redacted]

Table S6: Enzyme Release Kinetics: Analysis Repeated

Supplier	Product	Strength (Lipase)	Batch
Allergan	Panzytrat	25K	413201

[redacted]

**Part 2 - Enzyme Activity and Enzyme Release Kinetics**

6. RESULTS

Results for all tests are reported as described in each method.

Table S7: Lipase Activity Reported Results

Supplier	Product	Strength (Lipase)	Batch	Expiry	Labelled Lipase Activity / Dose Unit	Lipase Assay / Actual Activity per gram	Lipase Assay / Unit Dose	Lipase % of Label Claim
Abbott	Kreon	5K	59042	Nov-22	5000	52867	5287	105.7
Abbott	Kreon	10K	58519	Oct-21	10000	43907	11037	110.4
Abbott	Kreon	20K	58845	Dec-21	20000	42792	20368	101.8
Abbott	Kreon	25K	58888	Jan-22	25000	50795	24785	99.1
Abbott	Kreon	35K	59016	Dec-21	35000	52652	37101	106.0

Effective Date: 3/31/2021 4:45:06 AM

Effective ver 1.0

Part 2 - Enzyme Activity and Enzyme Release Kinetics

Supplier	Product	Strength (Lipase)	Batch	Expiry	Labelled Lipase Activity / Dose Unit	Lipase Assay / Actual Activity per gram	Lipase Assay / Unit Dose	Lipase % of Label Claim
Allergan	Panzytrat	10K	337801	Jan-21	10000	61923	12583	125.8
Allergan	Panzytrat	20K	358001	Oct-22	20000	66708	29615	148.1
Allergan	Panzytrat	25K	413201	Aug-21	25000	75995	27929	111.7
Allergan	Panzytrat	40K	670501	May-22	40000	87936	44413	111.0
Berlin-Chemie AG	Mezym	10K	98013	Mar-22	10000	34640	11466	114.7
Berlin-Chemie AG	Pangrol	10K	94166E	Nov-21	10000	62749	12444	124.4
Berlin-Chemie AG	Pangrol	20K	92027A	May-22	20000	44054	20900	104.5
Berlin-Chemie AG	Pangrol	25K	93255H	Sep-21	25000	66124	29268	117.1

Effective Date: 3/31/2021 4:45:06 AM

Part 2 - Enzyme Activity and Enzyme Release Kinetics

Supplier	Product	Strength (Lipase)	Batch	Expiry	Labelled Lipase Activity / Dose Unit	Lipase Assay / Actual Activity per gram	Lipase Assay / Unit Dose	Lipase % of Label Claim
Berlin-Chemie AG	Pangrol	40K	92019	Apr-22	40000	85626	42784	107.0
Cheplapharm	Cotazym	20K	507401	Apr-21	20000	69243	22327	111.6
Cheplapharm	Cotazym	30K	507701	May-21	30000	72283	33283	110.9
Cheplapharm	Cotazym	40K	659101	May-21	40000	94652	46081	115.2
Nordmark	Pankreatin	10K	012501	Apr-21	10000	60185	10648	106.5
Nordmark	Pankreatin	20K	319301	May-21	20000	60962	20634	103.2
Nordmark	Pankreatin	40K	672401	Jul-22	40000	87876	41555	103.9
Nordmark	Pankreatan	10K	321301	Apr-22	10000	58778	10516	105.2

Effective Date: 3/31/2021 4:45:06 AM

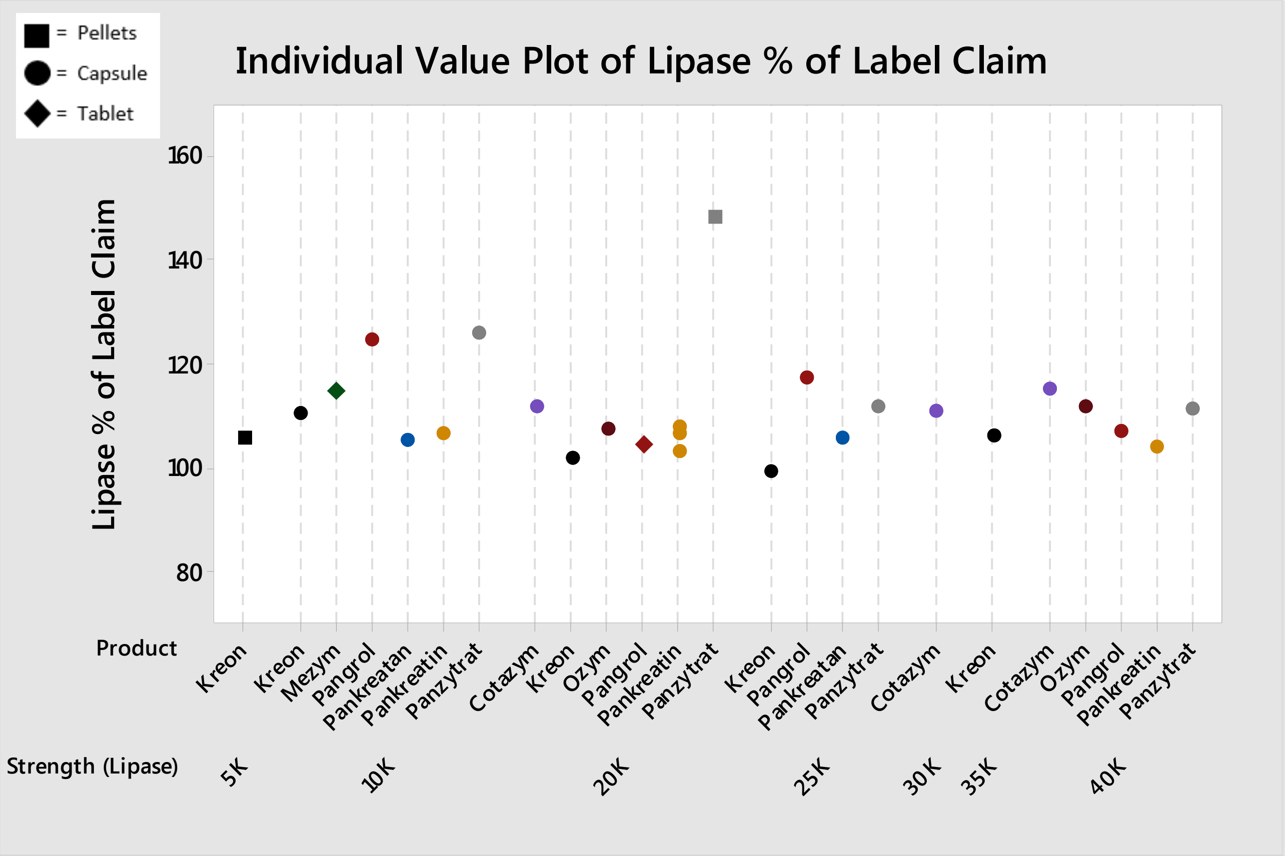

Figure S1: Lipase Activity as % of Label Claim

Effective Date: 3/31/2021 4:45:06 AM

Part 2 - Enzyme Activity and Enzyme Release Kinetics

Table S8: Amylase Activity Reported Results

Supplier	Product	Strength (Lipase)	Batch	Expiry	Labelled Amylase Activity / Dose Unit	Amylase Assay / Actual Activity per gram	Amylase Assay / Unit Dose	Amylase % of Label Claim
Abbott	Kreon	5K	59042	Nov-22	3600	54847	5649	156.9
Abbott	Kreon	10K	58519	Oct-21	8000	48229	12137	151.7
Abbott	Kreon	20K	58845	Dec-21	16000	50525	23967	149.8
Abbott	Kreon	25K	58888	Jan-22	18000	53376	25975	144.3
Abbott	Kreon	35K	59016	Dec-21	25200	51112	35906	142.5
Allergan	Panzytrat	10K	337801	Jan-21	9000	54033	11016	122.4
Allergan	Panzytrat	20K	358001	Oct-22	18000	54797	21172	117.6

Effective Date: 3/31/2021 4:45:06 AM

Effective ver 1.0

Part 2 - Enzyme Activity and Enzyme Release Kinetics

Supplier	Product	Strength (Lipase)	Batch	Expiry	Labelled Amylase Activity / Dose Unit	Amylase Assay / Actual Activity per gram	Amylase Assay / Unit Dose	Amylase % of Label Claim
Allergan	Panzytrat	25K	413201	Aug-21	15000	56597	20922	139.5
Allergan	Panzytrat	40K	670501	May-22	25000	60253	30131	120.5
Berlin-Chemie AG	Mezym	10K	98013	Mar-22	7500	32051	9213	122.8
Berlin-Chemie AG	Pangrol	10K	94166E	Nov-21	9000	60647	11728	130.3
Berlin-Chemie AG	Pangrol	20K	92027A	May-22	12000	46866	19244	160.4
Berlin-Chemie AG	Pangrol	25K	93255H	Sep-21	22500	63588	28363	126.1
Berlin-Chemie AG	Pangrol	40K	92019	Apr-22	25000	66277	33285	133.1
Cheplapharm	Cotazym	20K	507401	Apr-21	14500	63362	20292	139.9

Effective Date: 3/31/2021 4:45:06 AM

Part 2 - Enzyme Activity and Enzyme Release Kinetics

Supplier	Product	Strength (Lipase)	Batch	Expiry	Labelled Amylase Activity / Dose Unit	Amylase Assay / Actual Activity per gram	Amylase Assay / Unit Dose	Amylase % of Label Claim
Cheplapharm	Cotazym	30K	507701	May-21	21750	64716	30033	138.1
Cheplapharm	Cotazym	40K	659101	May-21	25000	60655	29655	118.6
Nordmark	Pankreatin	10K	012501	Apr-21	7250	54248	9665	133.3
Nordmark	Pankreatin	20K	319301	May-21	15000	55647	19216	128.1
Nordmark	Pankreatin	40K	672401	Jul-22	25000	61360	28979	115.9
Nordmark	Pankreatan	10K	321301	Apr-22	7500	57182	10030	133.7
Nordmark	Pankreatan	25K	323101	Jul-22	18750	52208	22667	120.9
Ratiopharm	Pankreatin	20K	321401	Feb-22	15000	57582	20545	137.0

Effective Date: 3/31/2021 4:45:06 AM

Effective ver 1.0

Part 2 - Enzyme Activity and Enzyme Release Kinetics

Supplier	Product	Strength (Lipase)	Batch	Expiry	Labelled Amylase Activity / Dose Unit	Amylase Assay / Actual Activity per gram	Amylase Assay / Unit Dose	Amylase % of Label Claim
StadaPharma/Aliud Pharma	Pankreatin	20K	92238	May-22	15000	51576	18362	122.4
Trommsdorff	Ozym	20K	N001	Dec-21	15000	56503	20609	137.4
Trommsdorff	Ozym	40K	N002	Feb-22	25000	64737	33539	134.2

Effective Date: 3/31/2021 4:45:06 AM

Effective ver 1.0

**Part 2 - Enzyme Activity and Enzyme Release Kinetics**

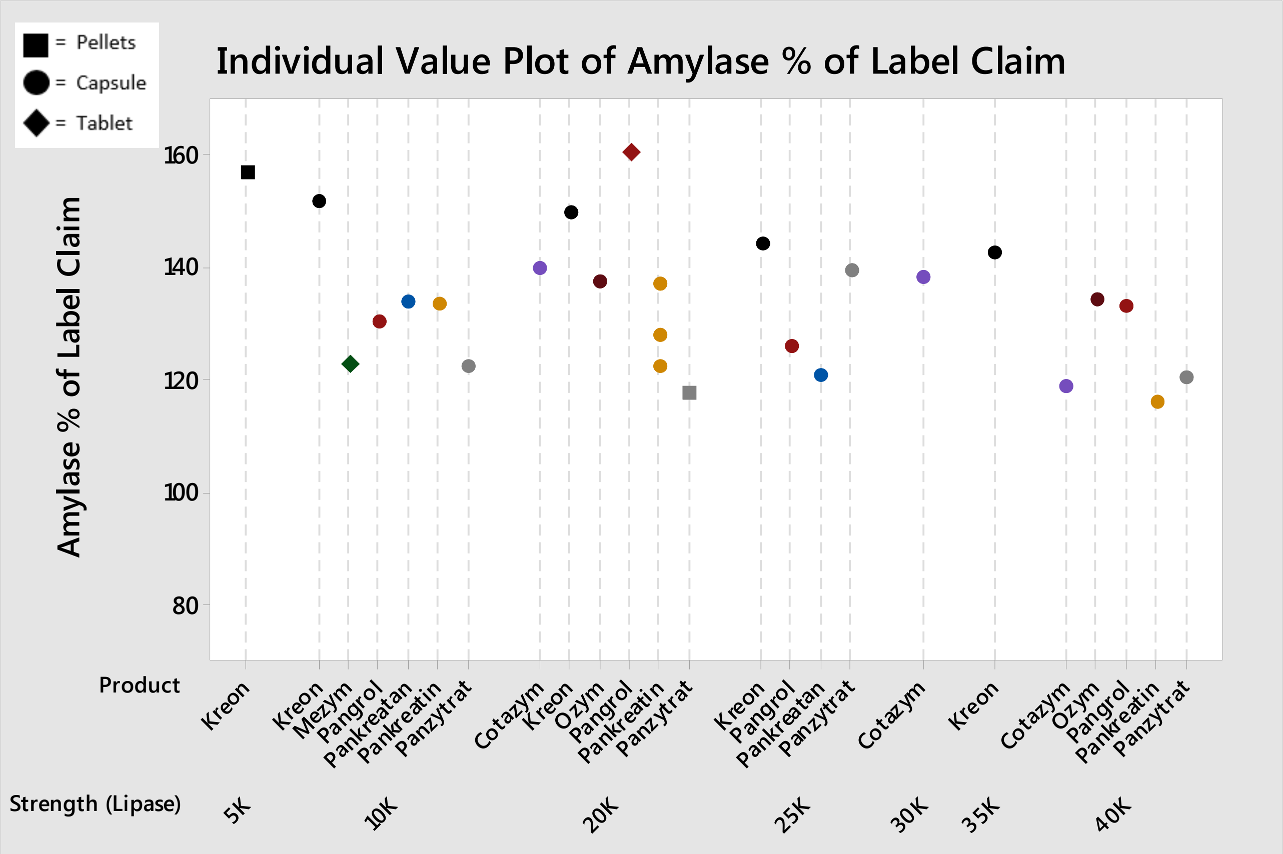

Figure S2: Amylase Activity as % of Label Claim

Effective Date: 3/31/2021 4:45:06 AM

Part 2 - Enzyme Activity and Enzyme Release Kinetics

Table S9: Total Protease Activity Reported Results

Supplier	Product	Strength (Lipase)	Batch	Expiry	Labelled Protease Activity / Dose Unit	Protease Assay / Actual Activity per gram	Protease Assay / Unit Dose	Protease % of Label Claim
Abbott	Kreon	5K	59042	Nov-22	200	3269	330	165.0
Abbott	Kreon	10K	58519	Oct-21	600	3132	790	131.7
Abbott	Kreon	20K	58845	Dec-21	1200	3263	1556	129.7
Abbott	Kreon	25K	58888	Jan-22	1000	3024	1477	147.7
Abbott	Kreon	35K	59016	Dec-21	1400	2961	2090	149.2
Allergan	Panzytrat	10K	337801	Jan-21	500	2889	599	119.7
Allergan	Panzytrat	20K	358001	Oct-22	1000	2863	1109	110.9

Effective Date: 3/31/2021 4:45:06 AM

Effective ver 1.0

Part 2 - Enzyme Activity and Enzyme Release Kinetics

Supplier	Product	Strength (Lipase)	Batch	Expiry	Labelled Protease Activity / Dose Unit	Protease Assay / Actual Activity per gram	Protease Assay / Unit Dose	Protease % of Label Claim
Allergan	Panzytrat	25K	413201	Aug-21	800	2786	1012	126.5
Allergan	Panzytrat	40K	670501	May-22	1500	3509	1761	117.4
Berlin-Chemie AG	Mezym	10K	98013	Mar-22	375	1535	506	135.0
Berlin-Chemie AG	Pangrol	10K	94166E	Nov-21	500	3249	633	126.6
Berlin-Chemie AG	Pangrol	20K	92027A	May-22	900	2714	1290	143.3
Berlin-Chemie AG	Pangrol	25K	93255H	Sep-21	1250	3421	1529	122.3
Berlin-Chemie AG	Pangrol	40K	92019	Apr-22	1500	3502	1735	115.7
Cheplapharm	Cotazym	20K	507401	Apr-21	850	3236	1041	122.5

Effective Date: 3/31/2021 4:45:06 AM

Effective ver 1.0

Part 2 - Enzyme Activity and Enzyme Release Kinetics

Supplier	Product	Strength (Lipase)	Batch	Expiry	Labelled Protease Activity / Dose Unit	Protease Assay / Actual Activity per gram	Protease Assay / Unit Dose	Protease % of Label Claim
Cheplapharm	Cotazym	30K	507701	May-21	1275	3704	1717	134.6
Cheplapharm	Cotazym	40K	659101	May-21	1500	3309	1601	106.7
Nordmark	Pankreatin	10K	012501	Apr-21	425	3214	567	133.5
Nordmark	Pankreatin	20K	319301	May-21	900	2874	987	109.7
Nordmark	Pankreatin	40K	672401	Jul-22	1500	3689	1742	116.1
Nordmark	Pankreatan	10K	321301	Apr-22	450	2872	504	112.0
Nordmark	Pankreatan	25K	323101	Jul-22	1125	2773	1207	107.3
Ratiopharm	Pankreatin	20K	321401	Feb-22	900	2834	997	110.8

Effective Date: 3/31/2021 4:45:06 AM

Effective ver 1.0

Part 2 - Enzyme Activity and Enzyme Release Kinetics

Supplier	Product	Strength (Lipase)	Batch	Expiry	Labelled Protease Activity / Dose Unit	Protease Assay / Actual Activity per gram	Protease Assay / Unit Dose	Protease % of Label Claim
StadaPharma/Aliud Pharma	Pankreatin	20K	92238	May-22	900	2937	1057	117.5
Trommsdorff	Ozym	20K	N001	Dec-21	900	2942	1060	117.8
Trommsdorff	Ozym	40K	N002	Feb-22	1500	3283	1667	111.1

Effective Date: 3/31/2021 4:45:06 AM

**Part 2 - Enzyme Activity and Enzyme Release Kinetics**

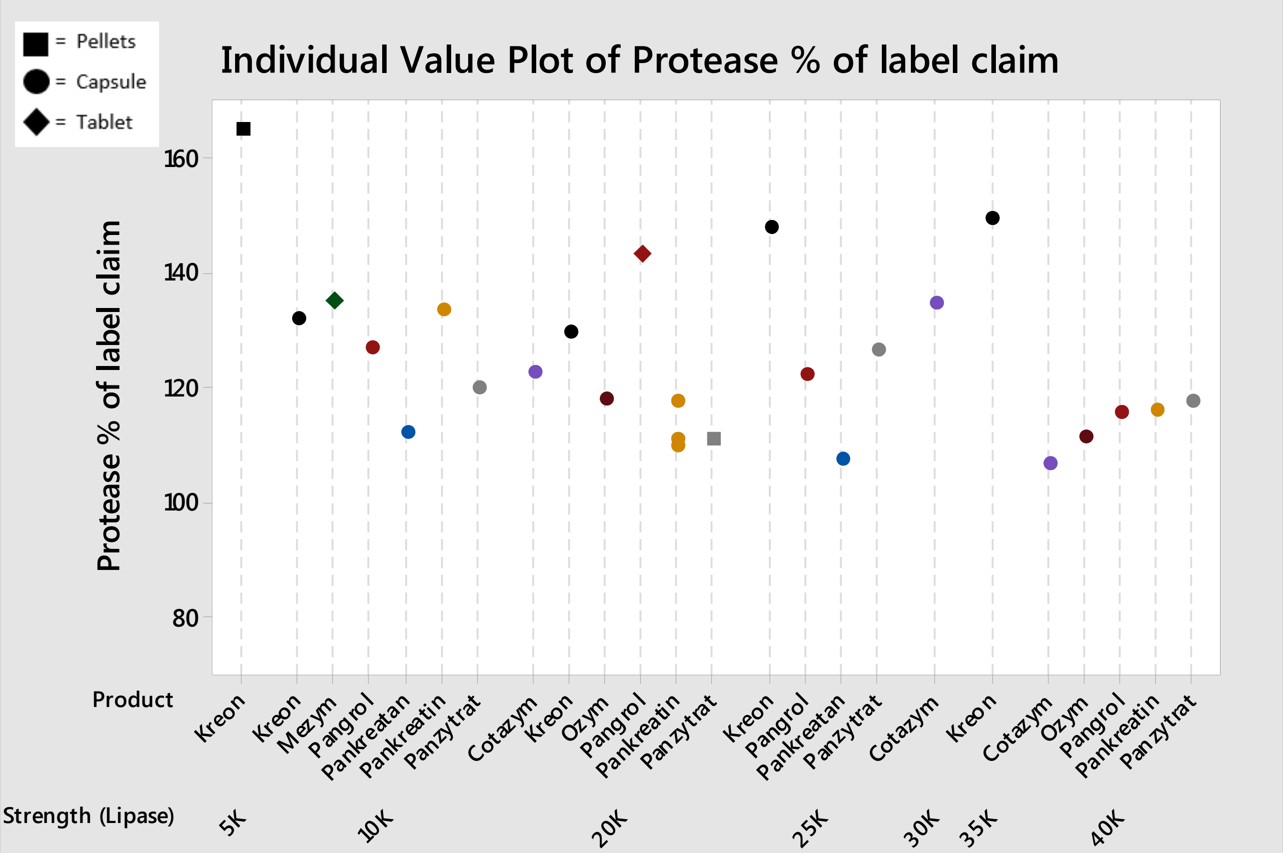

Figure S3: Protease Activity as % Label Claim

Effective Date: 3/31/2021 4:45:06 AM

Part 2 - Enzyme Activity and Enzyme Release Kinetics

Table S10: Enzyme Release Kinetics Reported Results (Residual Lipase Activity / Ph.Eur.-units)

Supplier	Product	Strength (Lipase)	Batch	Expiry	pH Test	Residual Lipase Activity / Ph.Eur.-units							
						15 mins	30 mins	45 mins	60 mins	75 mins	90 mins	105 mins	120 mins
Abbott	Kreon	5K	59042	Nov-22	1 to 6	0	0	0	0	9713	47794	44672	42760
Abbott	Kreon	5K	59042	Nov-22	4 to 6	1438	1508	1340	470	47249	43984	41989	38873
Abbott	Kreon	5K	59042	Nov-22	5 to 6	577	491	651	646	43921	47615	44749	43099
Abbott	Kreon	10K	58519	Oct-21	1 to 6	0	0	0	0	25416	37053	34684	34511
Abbott	Kreon	10K	58519	Oct-21	4 to 6	1784	1835	1131	1998	16605	36935	34964	35879
Abbott	Kreon	10K	58519	Oct-21	5 to 6	1978	2035	2509	2561	32937	37434	36157	34422
Abbott	Kreon	20K	58845	Dec-21	1 to 6	0	0	0	0	6599	36467	35084	34031

Effective Date: 3/31/2021 4:45:06 AM

Effective ver 1.0

Part 2 - Enzyme Activity and Enzyme Release Kinetics

Supplier	Product	Strength (Lipase)	Batch	Expiry	pH Test	Residual Lipase Activity / Ph.Eur.-units							
						15 mins	30 mins	45 mins	60 mins	75 mins	90 mins	105 mins	120 mins
Abbott	Kreon	20K	58845	Dec-21	4 to 6	773	1216	954	1073	26917	37852	36661	35298
Abbott	Kreon	20K	58845	Dec-21	5 to 6	348	1266	858	738	12833	37450	33122	31065
Abbott	Kreon	25K	58888	Jan-22	1 to 6	0	0	0	0	13972	46111	43165	41302
Abbott	Kreon	25K	58888	Jan-22	4 to 6	379	1205	1047	966	34856	47260	44015	43565
Abbott	Kreon	25K	58888	Jan-22	5 to 6	1078	1285	1348	1268	13465	45969	41640	41077
Abbott	Kreon	35K	59016	Dec-21	1 to 6	0	0	0	0	10162	40699	42351	41268
Abbott	Kreon	35K	59016	Dec-21	4 to 6	1436	1426	596	1185	28912	46002	44595	43347
Abbott	Kreon	35K	59016	Dec-21	5 to 6	1359	1350	1341	1776	26364	48125	45615	44866

Effective Date: 3/31/2021 4:45:06 AM

Effective ver 1.0

Part 2 - Enzyme Activity and Enzyme Release Kinetics

Supplier	Product	Strength (Lipase)	Batch	Expiry	pH Test	Residual Lipase Activity / Ph.Eur.-units							
						15 mins	30 mins	45 mins	60 mins	75 mins	90 mins	105 mins	120 mins
Allergan	Panzytrat	10K	337801	Jan-21	1 to 6	0	0	0	0	44824	46227	45916	43181
Allergan	Panzytrat	10K	337801	Jan-21	4 to 6	36867	23680	15739	11898	8911	9882	8619	7881
Allergan	Panzytrat	10K	337801	Jan-21	5 to 6	45055	37531	30453	26162	22070	22865	21256	21024
Allergan	Panzytrat	20K	358001	Oct-22	1 to 6	0	0	0	0	50599	48573	46471	41551
Allergan	Panzytrat	20K	358001	Oct-22	4 to 6	0	1246	4510	4830	48361	48205	44940	43944
Allergan	Panzytrat	20K	358001	Oct-22	5 to 6	29329	47376	40838	33399	30215	27884	27252	23921
Allergan	Panzytrat	25K	413201	Aug-21	1 to 6	0	0	0	0	66715	62449	59895	51951
Allergan	Panzytrat	25K	413201	Aug-21	4 to 6	41070	28083	22959	17901	15776	14428	13097	12228

Effective Date: 3/31/2021 4:45:06 AM

Effective ver 1.0

Part 2 - Enzyme Activity and Enzyme Release Kinetics

Supplier	Product	Strength (Lipase)	Batch	Expiry	pH Test	Residual Lipase Activity / Ph.Eur.-units							
						15 mins	30 mins	45 mins	60 mins	75 mins	90 mins	105 mins	120 mins
Allergan	Panzytrat	25K	413201	Aug-21	5 to 6	59558	47036	40179	34752	33572	26409	27442	26146
Allergan	Panzytrat	40K	670501	May-22	1 to 6	0	0	0	0	6558	31138	36882	44088
Allergan	Panzytrat	40K	670501	May-22	4 to 6	955	1541	2237	2923	17349	30645	38474	36952
Allergan	Panzytrat	40K	670501	May-22	5 to 6	3999	26397	47475	45511	41520	37487	32638	31302
Berlin-Chemie AG	Mezym	10K	98013	Mar-22	1 to 6	0	0	0	0	14638	28281	27538	26529
Berlin-Chemie AG	Mezym	10K	98013	Mar-22	4 to 6	15069	17720	12062	8651	7064	7016	6363	6496
Berlin-Chemie AG	Mezym	10K	98013	Mar-22	5 to 6	29854	26842	25491	24456	22772	21133	20362	19672
Berlin-Chemie AG	Pangrol	10K	94166E	Nov-21	1 to 6	0	0	0	0	43120	45142	46288	44897

Effective Date: 3/31/2021 4:45:06 AM

Part 2 - Enzyme Activity and Enzyme Release Kinetics

Supplier	Product	Strength (Lipase)	Batch	Expiry	pH Test	Residual Lipase Activity / Ph.Eur.-units							
						15 mins	30 mins	45 mins	60 mins	75 mins	90 mins	105 mins	120 mins
Berlin-Chemie AG	Pangrol	10K	94166E	Nov-21	4 to 6	4381	12330	13868	16100	33209	34398	33868	31888
Berlin-Chemie AG	Pangrol	10K	94166E	Nov-21	5 to 6	17455	34803	32511	37807	37153	34386	34454	33973
Berlin-Chemie AG	Pangrol	20K	92027A	May-22	1 to 6	0	0	0	95	10171	35361	35649	32940
Berlin-Chemie AG	Pangrol	20K	92027A	May-22	4 to 6	600	6158	11445	16561	21419	19770	18864	17443
Berlin-Chemie AG	Pangrol	20K	92027A	May-22	5 to 6	7185	19331	29687	30758	24528	26777	26449	24502
Berlin-Chemie AG	Pangrol	25K	93255H	Sep-21	1 to 6	0	0	0	0	52187	54949	52390	49960
Berlin-Chemie AG	Pangrol	25K	93255H	Sep-21	4 to 6	31177	36712	29030	25495	19453	18119	17596	16092
Berlin-Chemie AG	Pangrol	25K	93255H	Sep-21	5 to 6	47969	48494	39773	37524	31869	31246	30000	26742

Effective Date: 3/31/2021 4:45:06 AM

Effective ver 1.0

Part 2 - Enzyme Activity and Enzyme Release Kinetics

Supplier	Product	Strength (Lipase)	Batch	Expiry	pH Test	Residual Lipase Activity / Ph.Eur.-units							
						15 mins	30 mins	45 mins	60 mins	75 mins	90 mins	105 mins	120 mins
Berlin-Chemie AG	Pangrol	40K	92019	Apr-22	1 to 6	0	0	0	0	8017	39820	47331	51595
Berlin-Chemie AG	Pangrol	40K	92019	Apr-22	4 to 6	0	0	2210	1964	30034	44344	48011	50609
Berlin-Chemie AG	Pangrol	40K	92019	Apr-22	5 to 6	7814	28063	36059	31574	39638	39134	36551	33656
Cheplapharm	Cotazym	20K	507401	Apr-21	1 to 6	0	0	0	0	6392	23524	30189	42103
Cheplapharm	Cotazym	20K	507401	Apr-21	4 to 6	836	1453	4845	2048	18460	38896	44161	41458
Cheplapharm	Cotazym	20K	507401	Apr-21	5 to 6	6416	21667	31650	33217	31819	30876	26856	25240
Cheplapharm	Cotazym	30K	507701	May-21	1 to 6	0	0	0	0	3487	29835	43731	44317
Cheplapharm	Cotazym	30K	507701	May-21	4 to 6	0	0	0	668	19940	54021	49279	43838

Effective Date: 3/31/2021 4:45:06 AM

Effective ver 1.0

Part 2 - Enzyme Activity and Enzyme Release Kinetics

Supplier	Product	Strength (Lipase)	Batch	Expiry	pH Test	Residual Lipase Activity / Ph.Eur.-units							
						15 mins	30 mins	45 mins	60 mins	75 mins	90 mins	105 mins	120 mins
Cheplapharm	Cotazym	30K	507701	May-21	5 to 6	4585	26214	34203	31013	30865	29571	27539	25105
Cheplapharm	Cotazym	40K	659101	May-21	1 to 6	0	0	0	0	8219	35428	49237	62425
Cheplapharm	Cotazym	40K	659101	May-21	4 to 6	0	0	1882	3307	28920	55170	52390	51466
Cheplapharm	Cotazym	40K	659101	May-21	5 to 6	5565	39129	46235	44488	37135	35746	32542	30637
Nordmark	Pankreatin	10K	012501	Apr-21	1 to 6	0	0	0	0	3060	31997	44696	43144
Nordmark	Pankreatin	10K	012501	Apr-21	4 to 6	1712	3873	5255	7363	20432	34128	41863	41300
Nordmark	Pankreatin	10K	012501	Apr-21	5 to 6	4571	11497	15258	19921	35787	34882	31834	30869
Nordmark	Pankreatin	20K	319301	May-21	1 to 6	0	0	0	0	50658	50320	48070	44810

Effective Date: 3/31/2021 4:45:06 AM

Effective ver 1.0

Part 2 - Enzyme Activity and Enzyme Release Kinetics

Supplier	Product	Strength (Lipase)	Batch	Expiry	pH Test	Residual Lipase Activity / Ph.Eur.-units							
						15 mins	30 mins	45 mins	60 mins	75 mins	90 mins	105 mins	120 mins
Nordmark	Pankreatin	20K	319301	May-21	4 to 6	29369	29681	23602	18354	15285	14191	12946	12043
Nordmark	Pankreatin	20K	319301	May-21	5 to 6	43959	43927	36707	31815	29530	28223	25749	25059
Nordmark	Pankreatin	40K	672401	Jul-22	1 to 6	0	0	0	0	9055	34586	47819	51495
Nordmark	Pankreatin	40K	672401	Jul-22	4 to 6	0	0	880	874	22020	55108	58778	59224
Nordmark	Pankreatin	40K	672401	Jul-22	5 to 6	1542	7656	19358	28840	51008	47264	42758	45274
Nordmark	Pankreatan	10K	321301	Apr-22	1 to 6	0	0	0	0	34056	46841	42003	40527
Nordmark	Pankreatan	10K	321301	Apr-22	4 to 6	15703	28445	20416	16114	11812	11194	10584	10246
Nordmark	Pankreatan	10K	321301	Apr-22	5 to 6	19839	27386	35013	27840	27076	24274	22583	21448

Effective Date: 3/31/2021 4:45:06 AM

Effective ver 1.0

Part 2 - Enzyme Activity and Enzyme Release Kinetics

Supplier	Product	Strength (Lipase)	Batch	Expiry	pH Test	Residual Lipase Activity / Ph.Eur.-units							
						15 mins	30 mins	45 mins	60 mins	75 mins	90 mins	105 mins	120 mins
Nordmark	Pankreatan	25K	323101	Jul-22	1 to 6	0	0	0	0	53115	47556	43672	41695
Nordmark	Pankreatan	25K	323101	Jul-22	4 to 6	23975	29243	23046	18571	15829	14525	13576	12218
Nordmark	Pankreatan	25K	323101	Jul-22	5 to 6	45778	38633	32774	27355	26594	23798	23634	21959
Ratiopharm	Pankreatin	20K	321401	Feb-22	1 to 6	0	0	0	0	31249	47089	44864	41716
Ratiopharm	Pankreatin	20K	321401	Feb-22	4 to 6	38218	23860	19286	15439	11678	10279	10208	9393
Ratiopharm	Pankreatin	20K	321401	Feb-22	5 to 6	32773	37487	31748	26960	23841	20886	20360	18890
StadaPharma/Aliud Pharma	Pankreatin	20K	92238	May-22	1 to 6	0	0	0	0	49869	45197	43019	39749
StadaPharma/Aliud Pharma	Pankreatin	20K	92238	May-22	4 to 6	31064	24806	18458	12365	12486	11388	10389	10566

Effective Date: 3/31/2021 4:45:06 AM

Effective ver 1.0

Part 2 - Enzyme Activity and Enzyme Release Kinetics

Supplier	Product	Strength (Lipase)	Batch	Expiry	pH Test	Residual Lipase Activity / Ph.Eur.-units							
						15 mins	30 mins	45 mins	60 mins	75 mins	90 mins	105 mins	120 mins
StadaPharma/Aliud Pharma	Pankreatin	20K	92238	May-22	5 to 6	40165	34969	33229	27675	25966	24494	23775	20602
Trommsdorff	Ozym	20K	N001	Dec-21	1 to 6	0	0	0	0	47700	45553	41202	39037
Trommsdorff	Ozym	20K	N001	Dec-21	4 to 6	32475	25370	19108	14504	14610	13444	13189	11804
Trommsdorff	Ozym	20K	N001	Dec-21	5 to 6	43476	34295	29739	25779	24506	22569	20744	20687
Trommsdorff	Ozym	40K	N002	Feb-22	1 to 6	0	0	0	0	10665	44893	49327	55265
Trommsdorff	Ozym	40K	N002	Feb-22	4 to 6	2271	1858	4217	4843	28155	49064	51170	44304
Trommsdorff	Ozym	40K	N002	Feb-22	5 to 6	6024	22471	36848	36861	36425	31897	32192	28389

Effective Date: 3/31/2021 4:45:06 AM

Effective ver 1.0

Part 2 - Enzyme Activity and Enzyme Release Kinetics

Table S11: Enzyme Release Kinetics Reported Results (Residual Lipase Activity as % of Lipase Activity)

Supplier	Product	Strength (Lipase)	Batch	Expiry	pH Test	Residual Lipase Activity as % of Lipase Activity							
						15 mins	30 mins	45 mins	60 mins	75 mins	90 mins	105 mins	120 mins
Abbott	Kreon	5K	59042	Nov-22	1 to 6	0	0	0	0	18	90	84	81
Abbott	Kreon	5K	59042	Nov-22	4 to 6	3	3	3	1	89	83	79	74
Abbott	Kreon	5K	59042	Nov-22	5 to 6	1	1	1	1	83	90	85	82
Abbott	Kreon	10K	58519	Oct-21	1 to 6	0	0	0	0	58	84	79	79
Abbott	Kreon	10K	58519	Oct-21	4 to 6	4	4	3	5	38	84	80	82
Abbott	Kreon	10K	58519	Oct-21	5 to 6	5	5	6	6	75	85	82	78
Abbott	Kreon	20K	58845	Dec-21	1 to 6	0	0	0	0	15	85	82	80

Effective Date: 3/31/2021 4:45:06 AM

Effective ver 1.0

Part 2 - Enzyme Activity and Enzyme Release Kinetics

Supplier	Product	Strength (Lipase)	Batch	Expiry	pH Test	Residual Lipase Activity as % of Lipase Activity							
						15 mins	30 mins	45 mins	60 mins	75 mins	90 mins	105 mins	120 mins
Abbott	Kreon	20K	58845	Dec-21	4 to 6	2	3	2	3	63	88	86	82
Abbott	Kreon	20K	58845	Dec-21	5 to 6	1	3	2	2	30	88	77	73
Abbott	Kreon	25K	58888	Jan-22	1 to 6	0	0	0	0	28	91	85	81
Abbott	Kreon	25K	58888	Jan-22	4 to 6	1	2	2	2	69	93	87	86
Abbott	Kreon	25K	58888	Jan-22	5 to 6	2	3	3	2	27	90	82	81
Abbott	Kreon	35K	59016	Dec-21	1 to 6	0	0	0	0	19	77	80	78
Abbott	Kreon	35K	59016	Dec-21	4 to 6	3	3	1	2	55	87	85	82
Abbott	Kreon	35K	59016	Dec-21	5 to 6	3	3	3	3	50	91	87	85

Effective Date: 3/31/2021 4:45:06 AM

Effective ver 1.0

Part 2 - Enzyme Activity and Enzyme Release Kinetics

Supplier	Product	Strength (Lipase)	Batch	Expiry	pH Test	Residual Lipase Activity as % of Lipase Activity							
						15 mins	30 mins	45 mins	60 mins	75 mins	90 mins	105 mins	120 mins
Allergan	Panzytrat	10K	337801	Jan-21	1 to 6	0	0	0	0	72	75	74	70
Allergan	Panzytrat	10K	337801	Jan-21	4 to 6	60	38	25	19	14	16	14	13
Allergan	Panzytrat	10K	337801	Jan-21	5 to 6	73	61	49	42	36	37	34	34
Allergan	Panzytrat	20K	358001	Oct-22	1 to 6	0	0	0	0	76	73	70	62
Allergan	Panzytrat	20K	358001	Oct-22	4 to 6	0	2	7	7	72	72	67	66
Allergan	Panzytrat	20K	358001	Oct-22	5 to 6	44	71	61	50	45	42	41	36
Allergan	Panzytrat	25K	413201	Aug-21	1 to 6	0	0	0	0	88	82	79	68
Allergan	Panzytrat	25K	413201	Aug-21	4 to 6	54	37	30	24	21	19	17	16

Effective Date: 3/31/2021 4:45:06 AM

Part 2 - Enzyme Activity and Enzyme Release Kinetics

Supplier	Product	Strength (Lipase)	Batch	Expiry	pH Test	Residual Lipase Activity as % of Lipase Activity							
						15 mins	30 mins	45 mins	60 mins	75 mins	90 mins	105 mins	120 mins
Allergan	Panzytrat	25K	413201	Aug-21	5 to 6	78	62	53	46	44	35	36	34
Allergan	Panzytrat	40K	670501	May-22	1 to 6	0	0	0	0	7	35	42	50
Allergan	Panzytrat	40K	670501	May-22	4 to 6	1	2	3	3	20	35	44	42
Allergan	Panzytrat	40K	670501	May-22	5 to 6	5	30	54	52	47	43	37	36
Berlin-Chemie AG	Mezym	10K	98013	Mar-22	1 to 6	0	0	0	0	42	82	79	77
Berlin-Chemie AG	Mezym	10K	98013	Mar-22	4 to 6	44	51	35	25	20	20	18	19
Berlin-Chemie AG	Mezym	10K	98013	Mar-22	5 to 6	86	77	74	71	66	61	59	57
Berlin-Chemie AG	Pangrol	10K	94166E	Nov-21	1 to 6	0	0	0	0	69	72	74	72

Effective Date: 3/31/2021 4:45:06 AM

Effective ver 1.0

Part 2 - Enzyme Activity and Enzyme Release Kinetics

Supplier	Product	Strength (Lipase)	Batch	Expiry	pH Test	Residual Lipase Activity as % of Lipase Activity							
						15 mins	30 mins	45 mins	60 mins	75 mins	90 mins	105 mins	120 mins
Berlin-Chemie AG	Pangrol	10K	94166E	Nov-21	4 to 6	7	20	22	26	53	55	54	51
Berlin-Chemie AG	Pangrol	10K	94166E	Nov-21	5 to 6	28	55	52	60	59	55	55	54
Berlin-Chemie AG	Pangrol	20K	92027A	May-22	1 to 6	0	0	0	0	23	80	81	75
Berlin-Chemie AG	Pangrol	20K	92027A	May-22	4 to 6	1	14	26	38	49	45	43	40
Berlin-Chemie AG	Pangrol	20K	92027A	May-22	5 to 6	16	44	67	70	56	61	60	56
Berlin-Chemie AG	Pangrol	25K	93255H	Sep-21	1 to 6	0	0	0	0	79	83	79	76
Berlin-Chemie AG	Pangrol	25K	93255H	Sep-21	4 to 6	47	56	44	39	29	27	27	24
Berlin-Chemie AG	Pangrol	25K	93255H	Sep-21	5 to 6	73	73	60	57	48	47	45	40

Effective Date: 3/31/2021 4:45:06 AM

Effective ver 1.0

Part 2 - Enzyme Activity and Enzyme Release Kinetics

Supplier	Product	Strength (Lipase)	Batch	Expiry	pH Test	Residual Lipase Activity as % of Lipase Activity							
						15 mins	30 mins	45 mins	60 mins	75 mins	90 mins	105 mins	120 mins
Berlin-Chemie AG	Pangrol	40K	92019	Apr-22	1 to 6	0	0	0	0	9	47	55	60
Berlin-Chemie AG	Pangrol	40K	92019	Apr-22	4 to 6	0	0	3	2	35	52	56	59
Berlin-Chemie AG	Pangrol	40K	92019	Apr-22	5 to 6	9	33	42	37	46	46	43	39
Cheplapharm	Cotazym	20K	507401	Apr-21	1 to 6	0	0	0	0	9	34	44	61
Cheplapharm	Cotazym	20K	507401	Apr-21	4 to 6	1	2	7	3	27	56	64	60
Cheplapharm	Cotazym	20K	507401	Apr-21	5 to 6	9	31	46	48	46	45	39	36
Cheplapharm	Cotazym	30K	507701	May-21	1 to 6	0	0	0	0	5	41	60	61
Cheplapharm	Cotazym	30K	507701	May-21	4 to 6	0	0	0	1	28	75	68	61

Effective Date: 3/31/2021 4:45:06 AM

Effective ver 1.0

Part 2 - Enzyme Activity and Enzyme Release Kinetics

Supplier	Product	Strength (Lipase)	Batch	Expiry	pH Test	Residual Lipase Activity as % of Lipase Activity							
						15 mins	30 mins	45 mins	60 mins	75 mins	90 mins	105 mins	120 mins
Cheplapharm	Cotazym	30K	507701	May-21	5 to 6	6	36	47	43	43	41	38	35
Cheplapharm	Cotazym	40K	659101	May-21	1 to 6	0	0	0	0	9	37	52	66
Cheplapharm	Cotazym	40K	659101	May-21	4 to 6	0	0	2	3	31	58	55	54
Cheplapharm	Cotazym	40K	659101	May-21	5 to 6	6	41	49	47	39	38	34	32
Nordmark	Pankreatin	10K	012501	Apr-21	1 to 6	0	0	0	0	5	53	74	72
Nordmark	Pankreatin	10K	012501	Apr-21	4 to 6	3	6	9	12	34	57	70	69
Nordmark	Pankreatin	10K	012501	Apr-21	5 to 6	8	19	25	33	59	58	53	51
Nordmark	Pankreatin	20K	319301	May-21	1 to 6	0	0	0	0	83	83	79	74

Effective Date: 3/31/2021 4:45:06 AM

Effective ver 1.0

Part 2 - Enzyme Activity and Enzyme Release Kinetics

Supplier	Product	Strength (Lipase)	Batch	Expiry	pH Test	Residual Lipase Activity as % of Lipase Activity							
						15 mins	30 mins	45 mins	60 mins	75 mins	90 mins	105 mins	120 mins
Nordmark	Pankreatin	20K	319301	May-21	4 to 6	48	49	39	30	25	23	21	20
Nordmark	Pankreatin	20K	319301	May-21	5 to 6	72	72	60	52	48	46	42	41
Nordmark	Pankreatin	40K	672401	Jul-22	1 to 6	0	0	0	0	10	39	54	59
Nordmark	Pankreatin	40K	672401	Jul-22	4 to 6	0	0	1	1	25	63	67	67
Nordmark	Pankreatin	40K	672401	Jul-22	5 to 6	2	9	22	33	58	54	49	52
Nordmark	Pankreatan	10K	321301	Apr-22	1 to 6	0	0	0	0	58	80	71	69
Nordmark	Pankreatan	10K	321301	Apr-22	4 to 6	27	48	35	27	20	19	18	17
Nordmark	Pankreatan	10K	321301	Apr-22	5 to 6	34	47	60	47	46	41	38	36

Effective Date: 3/31/2021 4:45:06 AM

Effective ver 1.0

Part 2 - Enzyme Activity and Enzyme Release Kinetics

Supplier	Product	Strength (Lipase)	Batch	Expiry	pH Test	Residual Lipase Activity as % of Lipase Activity							
						15 mins	30 mins	45 mins	60 mins	75 mins	90 mins	105 mins	120 mins
Nordmark	Pankreatan	25K	323101	Jul-22	1 to 6	0	0	0	0	86	77	71	68
Nordmark	Pankreatan	25K	323101	Jul-22	4 to 6	39	48	37	30	26	24	22	20
Nordmark	Pankreatan	25K	323101	Jul-22	5 to 6	74	63	53	45	43	39	38	36
Ratiopharm	Pankreatin	20K	321401	Feb-22	1 to 6	0	0	0	0	50	76	72	67
Ratiopharm	Pankreatin	20K	321401	Feb-22	4 to 6	61	38	31	25	19	16	16	15
Ratiopharm	Pankreatin	20K	321401	Feb-22	5 to 6	53	60	51	43	38	34	33	30
StadaPharma/Aliud Pharma	Pankreatin	20K	92238	May-22	1 to 6	0	0	0	0	83	75	72	66
StadaPharma/Aliud Pharma	Pankreatin	20K	92238	May-22	4 to 6	52	41	31	21	21	19	17	18

Effective Date: 3/31/2021 4:45:06 AM

Effective ver 1.0

Part 2 - Enzyme Activity and Enzyme Release Kinetics

Supplier	Product	Strength (Lipase)	Batch	Expiry	pH Test	Residual Lipase Activity as % of Lipase Activity							
						15 mins	30 mins	45 mins	60 mins	75 mins	90 mins	105 mins	120 mins
StadaPharma/Aliud Pharma	Pankreatin	20K	92238	May-22	5 to 6	67	58	55	46	43	41	40	34
Trommsdorff	Ozym	20K	N001	Dec-21	1 to 6	0	0	0	0	81	77	70	66
Trommsdorff	Ozym	20K	N001	Dec-21	4 to 6	55	43	32	25	25	23	22	20
Trommsdorff	Ozym	20K	N001	Dec-21	5 to 6	74	58	50	44	41	38	35	35
Trommsdorff	Ozym	40K	N002	Feb-22	1 to 6	0	0	0	0	12	51	56	63
Trommsdorff	Ozym	40K	N002	Feb-22	4 to 6	3	2	5	6	32	56	58	51
Trommsdorff	Ozym	40K	N002	Feb-22	5 to 6	7	26	42	42	42	36	37	32

Effective Date: 3/31/2021 4:45:06 AM

**Part 2 - Enzyme Activity and Enzyme Release Kinetics**

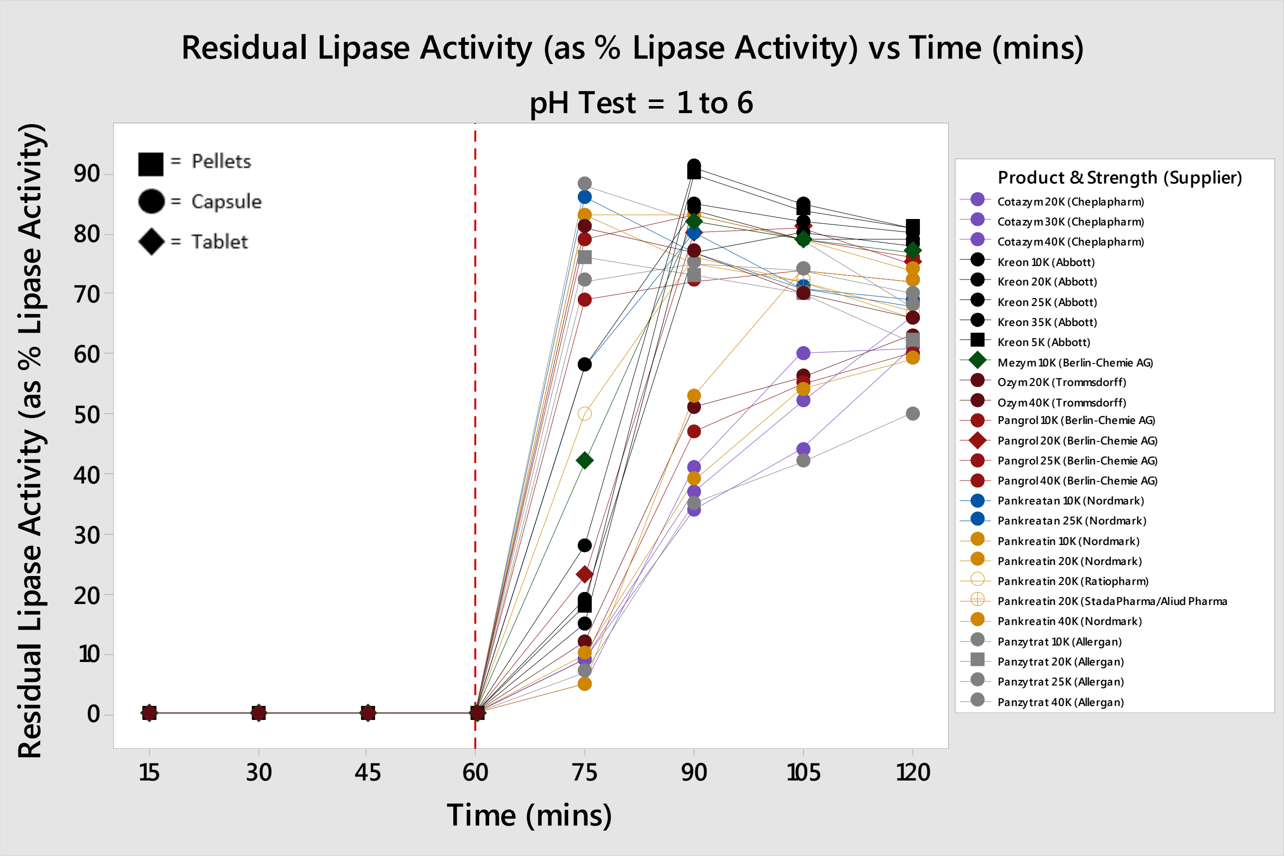

Figure S4: Residual Lipase Activity (as % Lipase Activity) vs Time-Point - pH Test = 1 to 6

Effective Date: 3/31/2021 4:45:06 AM

Part 2 - Enzyme Activity and Enzyme Release Kinetics

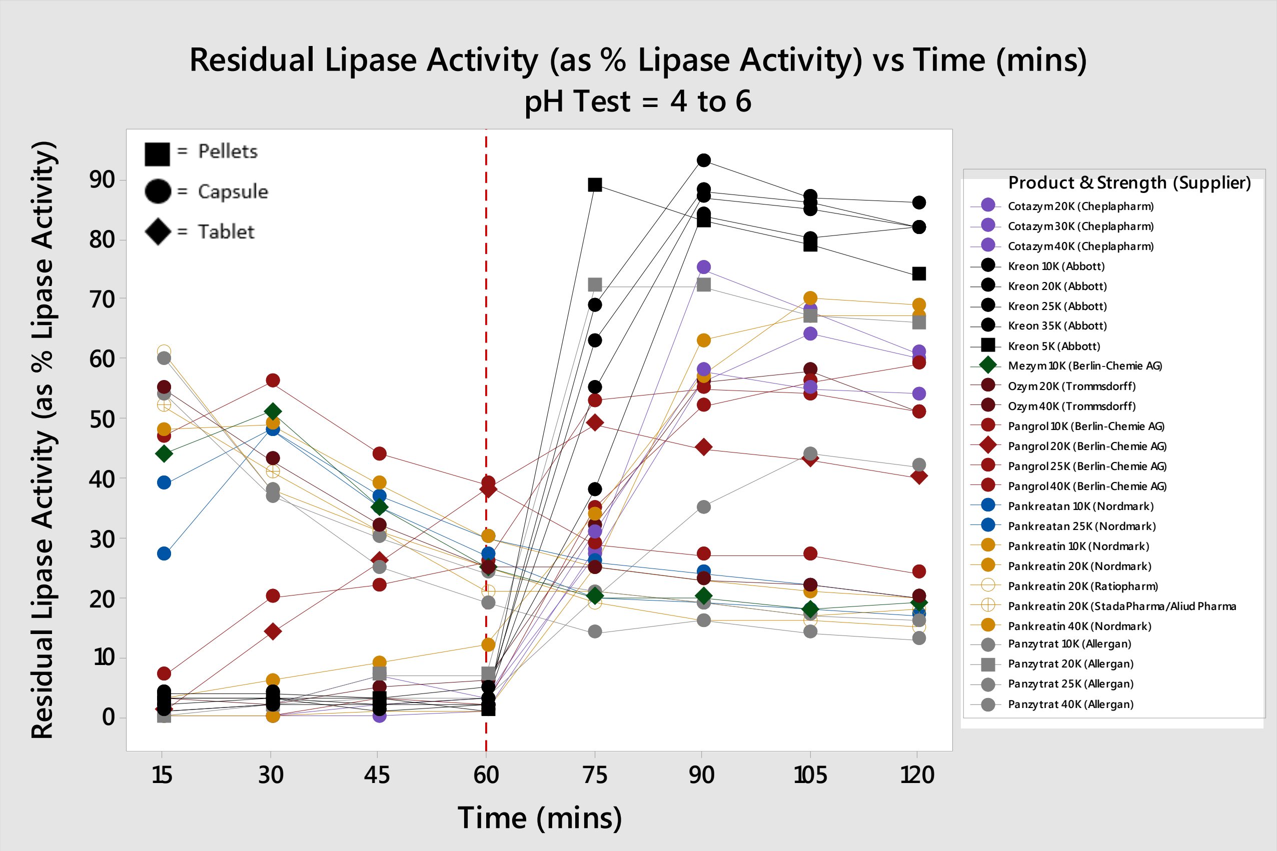

Figure S5: Residual Lipase Activity (as % Lipase Activity) vs Time-Point - pH Test = 4 to 6

Effective Date: 3/31/2021 4:45:06 AM

Effective ver 1.0

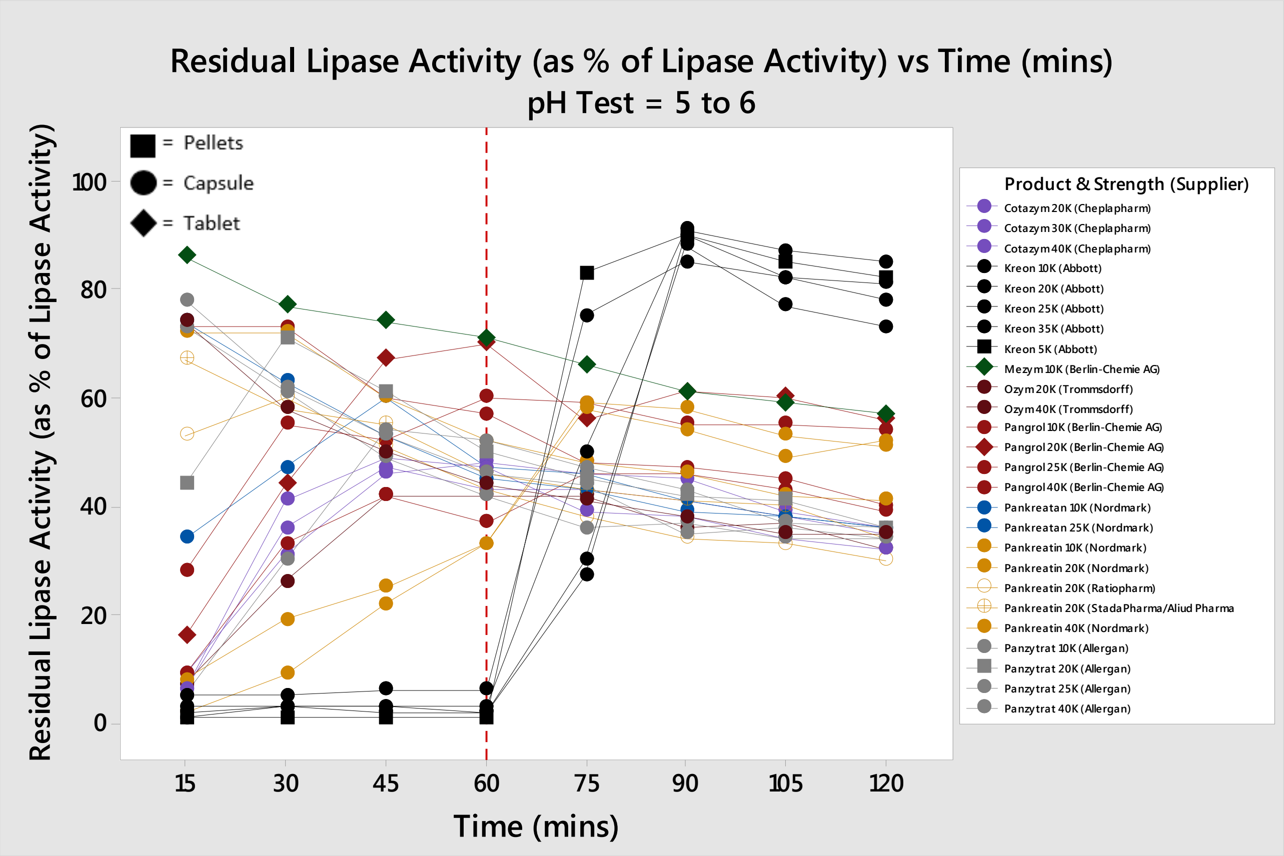

Figure S6: Residual Lipase Activity (as % Lipase Activity) vs Time-Point - pH Test = 5 to 6

Effective Date: 3/31/2021 4:45:06 AM

Effective ver 1.0

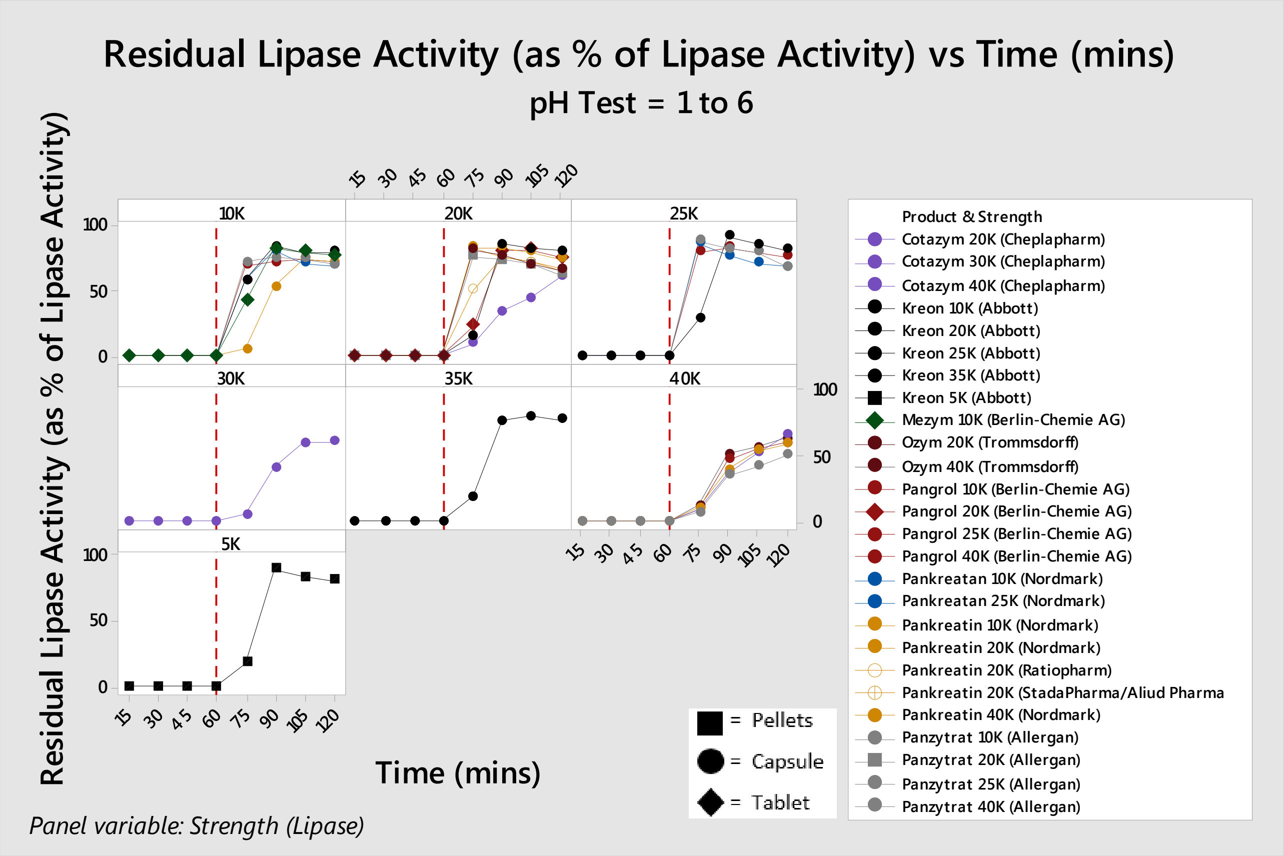

Figure S7: Residual Lipase Activity (as % of Lipase Activity) vs Time (mins) - pH Test = 1 to 6 Grouped by Individual Strength

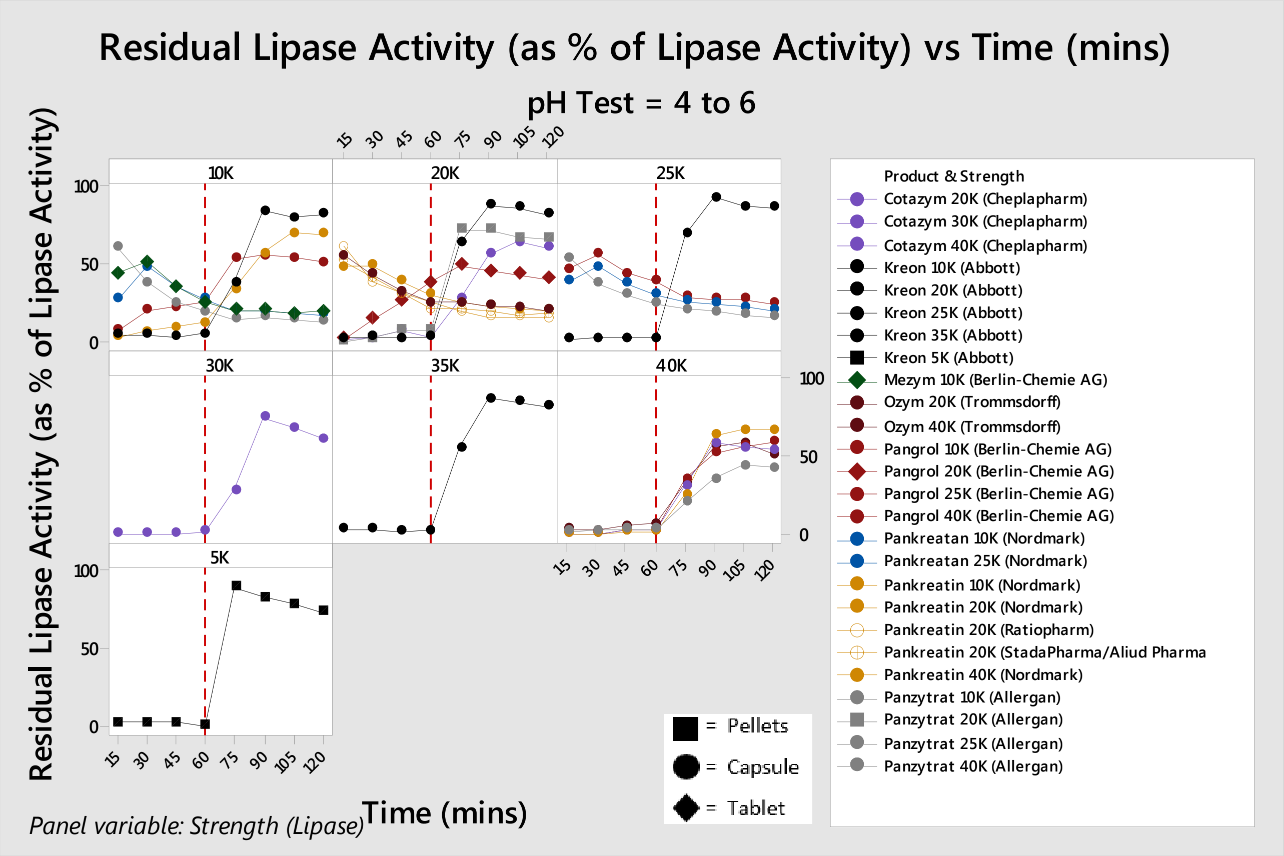

Figure S8: Residual Lipase Activity (as % of Lipase Activity) vs Time (mins) - pH Test = 4 to 6 Grouped by Individual Strength

Effective Date: 3/31/2021 4:45:06 AM

Effective ver 1.0

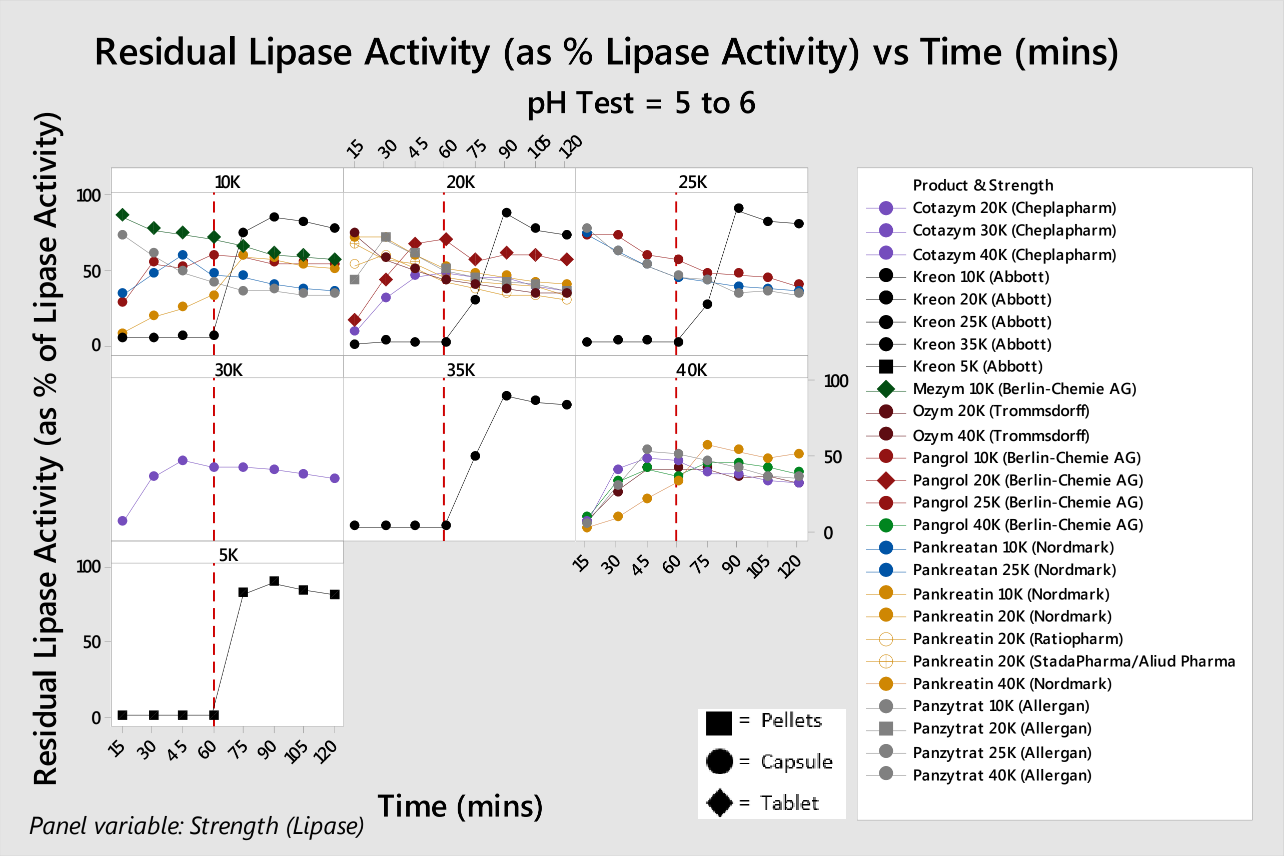

Figure S9: Residual Lipase Activity (as % of Lipase Activity) vs Time (mins) - pH Test = 5 to 6 Grouped by Individual Strength

Effective Date: 3/31/2021 4:45:06 AM

Effective ver 1.0

**Part 2 - Enzyme Activity and Enzyme Release Kinetics**

7. VERSION HISTORY

Version	Description of Change
1.0	Initial

Effective Date: 3/31/2021 4:45:06 AM

Effective ver 1.0

**Part 2 - Enzyme Activity and Enzyme Release Kinetics**

APPENDIX 1 – SAMPLE DETAILS

Supplier	Product	Strength (Lipase)	Batch	Expiry	Dosage Form
Abbott	Kreon	5K	59042	Nov-22	Pellets
Abbott	Kreon	10K	58519	Oct-21	Capsule
Abbott	Kreon	20K	58845	Dec-21	Capsule
Abbott	Kreon	25K	58888	Jan-22	Capsule
Abbott	Kreon	35K	59016	Dec-21	Capsule
Allergan	Panzytrat	10K	337801	Jan-21	Capsule
Allergan	Panzytrat	20K	358001	Oct-22	Pellets
Allergan	Panzytrat	25K	413201	Aug-21	Capsule
Allergan	Panzytrat	40K	670501	May-22	Capsule
Berlin-Chemie AG	Mezym	10K	98013	Mar-22	Tablet
Berlin-Chemie AG	Pangrol	10K	94166E	Nov-21	Capsule
Berlin-Chemie AG	Pangrol	20K	92027A	May-22	Tablet
Berlin-Chemie AG	Pangrol	25K	93255H	Sep-21	Capsule
Berlin-Chemie AG	Pangrol	40K	92019	Apr-22	Capsule
Cheplapharm	Cotazym	20K	507401	Apr-21	Capsule
Cheplapharm	Cotazym	30K	507701	May-21	Capsule
Cheplapharm	Cotazym	40K	659101	May-21	Capsule
Nordmark	Pankreatin	10K	012501	Apr-21	Capsule
Nordmark	Pankreatin	20K	319301	May-21	Capsule
Nordmark	Pankreatin	40K	672401	Jul-22	Capsule
Nordmark	Pankreatan	10K	321301	Apr-22	Capsule
Nordmark	Pankreatan	25K	323101	Jul-22	Capsule
Ratiopharm	Pankreatin	20K	321401	Feb-22	Capsule
StadaPharma/Aliud Pharma	Pankreatin	20K	92238	May-22	Capsule
Trommsdorff	Ozym	20K	N001	Dec-21	Capsule
Trommsdorff	Ozym	40K	N002	Feb-22	Capsule

Effective Date: 3/31/2021 4:45:06 AM

Effective ver 1.0
